# Associations between statins and adverse events in secondary prevention of cardiovascular disease: Pairwise, network, and dose-response meta-analyses of 47 randomized controlled trials

**DOI:** 10.3389/fcvm.2022.929020

**Published:** 2022-08-25

**Authors:** Xinyi Wang, Jingen Li, Tongxin Wang, Zihao Zhang, Qiuyi Li, Dan Ma, Zhuo Chen, Jianqing Ju, Hao Xu, Keji Chen

**Affiliations:** ^1^National Clinical Research Center for Chinese Medicine Cardiology, Xiyuan Hospital, China Academy of Chinese Medical Sciences, Beijing, China; ^2^Dongzhimen Hospital, Beijing University of Chinese Medicine, Beijing, China; ^3^Graduate School, Beijing University of Chinese Medicine, Beijing, China

**Keywords:** statin, prevention, adverse events, meta-analysis, randomized controlled trials

## Abstract

**Objectives:**

To explore the associations between different types and doses of statins and adverse events in secondary prevention of cardiovascular disease.

**Methods:**

We searched PubMed, Embase, and Cochrane databases for randomized controlled trials that compared statins with non-statin controls or different types or doses of statins. The primary outcomes included muscle condition, transaminase elevations, renal insufficiency, gastrointestinal discomfort, cancer, new onset or exacerbation of diabetes, cognitive impairment, and eye condition. We also analyzed myocardial infarction (MI), stroke, death from cardiovascular diseases (CVD), and all-cause death as the secondary outcomes to compare the potential harms with the benefits of statins. We conducted pairwise meta-analyses to calculate the odds ratio (OR) and 95% confidence intervals (CIs) for each outcome. Network meta-analyses were performed to compare the adverse effects of different statins. An Emax model was used to examine the dose-response relationships of the adverse effects of each statin.

**Results:**

Forty-seven trials involving 107,752 participants were enrolled and followed up for 4.05 years. Compared with non-statin control, statins were associated with an increased risk of transaminase elevations [OR 1.62 (95% CI 1.20 to 2.18)]. Statins decreased the risk of MI [OR 0.66 (95% CI 0.61 to 0.71), *P* < 0.001], stroke [OR 0.78 (95% CI 0.72 to 0.84), *P* < 0.001], death from CVD [OR 0.77 (95% CI 0.72 to 0.83), *P* < 0.001] and all-cause death [OR 0.83 (95% CI 0.79 to 0.88), *P* < 0.001]. Atorvastatin showed a higher risk of transaminase elevations than non-statin control [OR 4.0 (95% CI 2.2 to 7.6)], pravastatin [OR 3.49 (95% CI 1.77 to 6.92)] and simvastatin [OR 2.77 (95% CI 1.31 to 5.09)], respectively. Compared with atorvastatin, simvastatin was associated with a lower risk of muscle problems [OR 0.70 (95% CI 0.55 to 0.90)], while rosuvastatin showed a higher risk [OR 1.75 (95% CI 1.17 to 2.61)]. An Emax dose-response relationship was identified for the effect of atorvastatin on transaminase elevations.

**Conclusion:**

Statins were associated with increased risks of transaminases elevations in secondary prevention. Our study provides the ranking probabilities of statins that can help clinicians make optimal decisions when there is not enough literature to refer to.

**Systematic review registration:**

[https://www.crd.york.ac.uk/prospero/], identifier [CRD42021285161].

## Introduction

Statins are widely used in clinical practice and recommended as first-line treatment for atherosclerotic cardiovascular diseases (ASCVD) (1). However, various adverse events documented in clinical trials were considered statin-related, such as muscle problems and elevated hepatic transaminase (2, 3). Although other types of lipid-lowering agents are available (e.g., ezetimibe, niacin, PCSK9), so far there are no published trials with any of these new drugs in patients who are intolerant to statins. Physicians often face the dilemma of choosing optimal statins with the best efficacy and least adverse effects. Although previous study has reported comparative effectiveness and safety of statins as a class and of specific statins (4) in primary prevention in which usually a lower-intensity or dose was use, current guideline recommends that patients should be treated with the maximum-appropriate intensity of a statin that does not cause adverse effects for patients with ASCVD (5). In addition, although high-intensity statin therapy has been shown to reduce ASCVD events better than moderate or lower-intensity statin therapy (5), it is also associated with a greater risk of statin-induced adverse events (6). Therefore, it is of great necessity to assess the association of adverse events with the types and doses of statins in patients with ASCVD. The hypothesis suggested that the types and doses of statins may be related to different adverse reactions (7). Current suggestions on the type and dose of statins are based on their lipid-lowering effect, without considering the varying adverse reactions of different schemes. Understanding the relationship between the types and doses of statins and specific adverse events can help clinicians make appropriate choices. Therefore, we systematically reviewed randomized controlled trials (RCTs) in secondary prevention to evaluate the associations between statins and adverse events, and to explore how the associations vary by type and dose of statin.

## Methods

The study was conducted according to the Preferred Reporting Items for Systematic Reviews and Meta-Analyses (PRISMA) (8). The study protocol was registered on PROSPERO (CRD42021285161).

### Search methods and resources

Studies were searched from PubMed, Embase and the Cochrane Database from their inception to October 2021. Also, we checked previous systematic reviews of clinical trials of statins to avoid omission. Supplementary Table 1 shows the detailed search strategies.

### Selection of studies

Eligible studies were RCTs in patients with established ASCVD [i.e., coronary heart disease (CHD), peripheral artery disease, or cerebrovascular disease (5)], which compared statins with non-statin controls or compared different types or dosages of statins and reported at least one primary outcome of interest. Statin treatments were monotherapy or add-on treatment to routine care or non-drug treatments (e.g., diet or exercise). Non-statin controls included placebo, no treatment, and routine care. We also included studies involving > 60% of patients with established ASCVD to avoid the loss of large trials with a small proportion of patients without ASCVD. In instances where subgroup data for ASCVD patients was unpublished, the authors were contacted to request the data. Studies that enrolled < 100 patients (to exclude small studies with unreliable hazard ratios) or lasted for < 4 weeks of intervention were excluded (9). The eligibility criteria were detailly described in Supplementary Table 2. Two reviewers (XW and JL) independently screened titles and abstracts of all items and identified eligible trials. Discrepancies were resolved by consensus.

### Study outcomes

The primary outcomes were reported adverse events in previous clinical trials, including muscle condition, transaminase elevations, renal insufficiency, gastrointestinal discomfort, cancer, new-onset or exacerbation of diabetes, cognitive impairment, and eye condition. Muscle condition included self-reported muscle symptoms (i.e., myalgia, muscle weakness, and other non-specified muscle discomforts) and clinically confirmed muscle disorders (i.e., myopathy and rhabdomyolysis) (10). Transaminase elevations referred to incidence of elevations in serum aspartate aminotransferase (AST) and alanine aminotransferase (ALT). Renal insufficiency included any decline in renal function, the presence of proteinuria, and other diagnosed renal disorders. Gastrointestinal discomfort included nausea, vomiting, dyspepsia, constipation, abdominal pain and other symptoms related to the digestive system. Cancer referred to the incidence of any cancer, excluding non-melanoma skin cancer. New onset or exacerbation of diabetes (type 2 diabetes), cognitive impairment, and eye conditions were defined as the diagnoses in the original trials. We also analyzed myocardial infarction (MI), stroke, death from cardiovascular diseases (CVD), and all-cause death as the secondary outcomes to compare the potential harms with the benefits of statins.

### Data extraction and quality assessment

Two reviewers (XW and JL) independently extracted the information on study design, characteristics of participants, interventions, controls, outcome measurements, details relevant to the risk of bias and the quality of the evidence. The risk of bias in individual studies was evaluated by the Cochrane risk of bias tool (11, 12). The quality of evidence for each outcome in the pairwise meta-analyses and significant results in the network meta-analyses were evaluated based on the GRADE (Grading of Recommendations Assessment, Development, and Evaluation) process (12, 13). Any discrepancy that appeared during the data extraction or evaluation process was resolved through discussion.

### Statistical analysis

Pairwise meta-analyses were conducted to compare the effect of statins and non-statin controls for each outcome. Heterogeneity among individual studies was assessed with the Q test and quantified with the I^2^ statistic (14). When no significant heterogeneity was detected (*P* > 0.05 for the Q test and I^2^ < 50%), a fixed-effects model was employed to calculate pooled odds ratios (OR) with 95% confidence intervals (CIs); otherwise, a random-effects model was used (15). Publication bias was examined by the Harbord test of the symmetry of funnel plots. The robustness of the pooled results was tested by leave-one-out influence analysis (16). For sensitivity analyses, studies with a non-double-blind design were excluded to examine the effect of placebo. Because the evidence showed that Asians were less tolerant to statins (17), we further excluded studies on Asian populations to investigate the influence of race/ethnicity. For further sensitivity analysis, we excluded studies or individuals with transaminase elevation < 3 times the upper limit of normal (ULN) for the outcome of transaminase elevations because the rise in serum concentration of transaminase to more than three times the ULN was often considered to be a mark of liver dysfunction. In addition, the random-effects model was used for all outcomes as the further sensitivity analysis.

We performed a network meta-analysis to compare the adverse effects between different types of statins and non-statin controls by the Bayesian method (18). A random-effects model was employed to calculate the pooled OR and 95% CI instead of a fixed-effects model because the former measure provided more conservative results (19). As an alternative method for inconsistency assessment in network meta-analysis, node-splitting analysis was used to evaluate the consistency of data (20). *P* value of node-splitting analysis > 0.05 indicates no significant inconsistency. We calculated the ranking probabilities for each treatment’s efficacy. Probability values were reported using surface under the cumulative ranking (SUCRA) values. A higher SUCRA value indicated a better outcome for that intervention (21).

A model-based meta-analysis method that fitted the dose-specific effects from a network meta-analysis to an Emax dose-response model was employed to examine the dose-response relationship of the adverse effects of individual statins (22, 23). The key parameter Emax represents the asymptotic maximum drug effect, and ED50 means the dose that produces half of the maximum effect (24). Posterior means and 95% CI of the model parameters were estimated by the Bayesian approach (22). We analyzed outcomes using the non-statin control as a reference and ranked different statins with SUCRA probabilities based on the dose-response relationship.

All statistical tests had a two-tailed significance level of *P* ≤ 0.05. Analyses were performed in R version 4.0.1 with meta, metafor, gemtc, rjags, and MBNMAdose packages.

## Results

**FIGURE 1:**
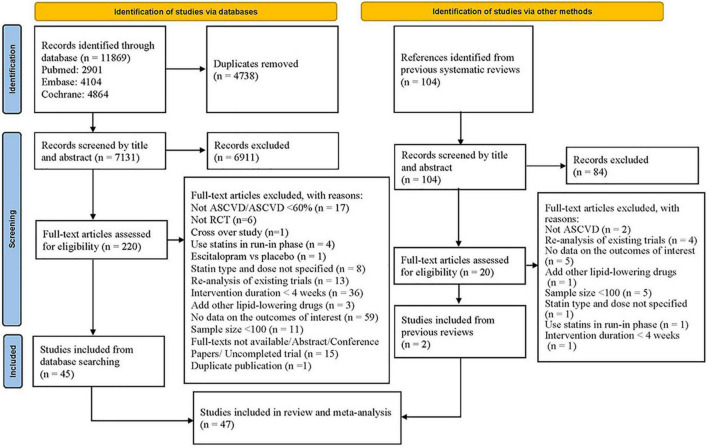
Flowchart of study selection.

Our searches identified 11,973 citations (11,869 from database searches and 104 from previous meta-analyses). Finally, after assessing the full text, forty-seven eligible studies (25–71) were included (Figure 1). Supplementary Table 3 presents the list of studies excluded after assessing the full text and reasons for exclusion.

### Study characteristics

A total of 107,752 participants were enrolled and followed up for a mean of 4.05 years. The mean age of all participants was 62 years old, and 77% were men. The target populations in the included studies were various. Thirty-five trials enrolled patients with CHD, and other trials enrolled patients with cerebrovascular disease (4 studies) and ASCVD (2 studies), or ≥ 60% ASCVD (6 studies), respectively. Eighteen studies compared statins with non-statin controls that included placebo (14 studies) and no treatment (4 studies). Seven types of statins were evaluated: atorvastatin (29 studies), fluvastatin (3 studies), lovastatin (1 study), pitavastatin (2 studies), pravastatin (10 studies), rosuvastatin (8 studies), and simvastatin (12 studies). The characteristics of the included studies are shown in Table 1.

**TABLE 1:** Characteristics of included studies.

Author, year	No of participants	Country	Study duration	Study population	Mean age	Proportion of men (%)	Statin treatment (dose, mg/day)	Comparator
MARS, (25)	247	United States	2.2 years	CHD	58	91	Lovastatin (80)	Placebo
Oxford Cholesterol, (26)	621	United Kingdom	44 months	ASCVD > 60%	63	85	Simvastatin (20/40)	Placebo
4S, (27)	4,444	Scandinavia	5.4 years	CHD	51% ≥ 60 year	82	Simvastatin 20	Placebo
PLAC I, (28)	408	United Kingdom, United States, Canada	3 years	CHD	57	38	Pravastatin (40)	Placebo
CARE, (29)	4,159	United States, Canada	5 years	MI	59	86	Pravastatin (40)	Placebo
LIPID, (30)	9,014	Australia, New Zealand	6.1 years	MI/unstable angina	62	83	Pravastatin (40)	Placebo
TARGET TANGIBLE, (31)	2,856	Germany	3.5 months	CHD	61	63	Atorvastatin (10–40), Simvastatin (10–40)	Different statin types
FLARE, (32)	834	Europe	10 months	PTCA	61	83	Fluvastatin (80)	Placebo
MIRACL, (33)	3,086	Europe, North America, South Africa and Australasia	4 months	MI/unstable angina	65	65	Atorvastatin (80)	Placebo
Karalis et al., (34)	1,595	United States	1.5 months	ASCVD > 60%	61.5	62	Atorvastatin (10/80), Simvastatin (20/80)	Different statin types and doses
LIPS, (35)	1,677	Europe, Canada and Brazil	3.9 years	Stable or unstable angina	60	84	Fluvastatin (80)	Placebo
HPS, (36)	20,536	United Kingdom	5 years	ASCVD > 60%	Not mentioned	75	Simvastatin (40)	Placebo
3T, (37)	1,093	Denmark, Finland, Iceland, Norway, and Sweden	13 months	CHD	63	75	Atorvastatin (20–40), Simvastatin (20–40)	Different statin types
REVERSAL, (38)	654	United States	4.5 months	CHD	56	72	Pravastatin (40), Atorvastatin (80)	Different statin types
Schwartz et al., (39)	383	United States and Canada	4.5 months	ASCVD	62	61	Rosuvastatin (5–80), Atorvastatin (10–80)	Different statin types
PROVE IT–TIMI 22, (40)	4,162	349 sites in eight countries	2 years	ACS	58	78	Pravastatin (40), Atorvastatin (80)	Different statin types
JUST, (41)	299	Japan	2 years	CHD	59	77	Simvastatin (10)	No treatment
IDEAL, (42)	8,888	Northern Europe	4.8 years	MI	62	81	Atorvastatin (80), Simvastatin (20)	Different statin types
TNT, (43)	10,001	14 countries worldwide	4.9 years	CHD	61	81	Atorvastatin (10/80)	Different statin doses
ATHEROMA, (44)	361	Japan	3 years	CHD	59	83	Pravastatin (10–20)	No treatment
SPARCL, (45)	4,731	205 centers worldwide	4.9 years	Stroke or TIA	63	60	Atorvastatin (80)	Placebo
SOLAR, (46)	1,621	United States	3 months	ASCVD > 60%	62	58	Rosuvastatin (10–20), Atorvastatin (10–20), Simvastatin (20–40)	Different statin types
ARIANE, (47)	844	France	3 months	ASCVD > 60%	63	76	Atorvastatin (10), Rosuvastatin (10)	Different statin types
Yun et al., (48)	155	South Korea	10 months	ACS/stroke	63	60	Rosuvastatin (10), Atorvastatin (20)	Different statin types
Yu et al., (49)	112	China	6.5 months	CHD	66	82	Atorvastatin (10/80)	Different statin doses
SAGE, (50)	891	192 sites worldwide in 16 countries	1 year	CHD	72	70	Atorvastatin (80), Pravastatin (40)	Different statin types
CAP, (51)	340	Canada and Europe	6.5 months	CHD	63	83	Atorvastatin (10/80)	Different statin doses
JAPAN-ACS, (52)	296	Japan	1 year	ACS + PCI	63	82	Pitavastatin (4), Atorvastatin (20)	Different statin types
Zhao et al., (53)	164	China	2 months	Unstable angina	71	65	Atorvastatin (20/80)	Different statin doses
SPACE ROCKET, (54)	1,263	United Kingdom	3 months	MI	62	79	Simvastatin (40), Rosuvastatin (10)	Different statin types
Mok et al., (55)	227	United Kingdom	2 years	MCA stenosis	63	34	Simvastatin (20)	Placebo
CENTAURUS, (56)	829	Belgium, Canada, Estonia, France, Greece, Hungary, Ireland, Italy, Portugal, Spain and Tunisia	3 months	NSTEACS	60	75	Rosuvastatin (20); Atorvastatin (80)	Different statin types
FACS, (57)	156	Czechia	1 month	ACS	62	68	Fluvastatin (80)	Placebo
SEARCH, (58)	12,064	United Kingdom	6.7 years	MI	64	83	Simvastatin (20/80)	Different statin doses
LUNAR, (59)	799	United States, Costa Rica and Panama	3 months	ACS	53	76	Rosuvastatin (20/40), Atorvastatin (80)	Different statin types and doses
TRUTH, (60)	154	Japan	8 months	Stable/unstable angina + PCI	67	83	Pitavastatin (4), Pravastatin (20)	Different statin types
CURE-ACS, (61)	173	India	3 months	ACS	56	82	Atorvastatin (40/80)	Different statin doses
PACT, (62)	3,408	Australia, Poland, Southeast Asian	1 month	MI/unstable angina	Not mentioned	76	Pravastatin (20–40)	Placebo
Zhou et al., (63)	112	China	13 months	AICAS	63	68	Atorvastatin (10/20/40)	Different statin doses
Khurana et al., (64)	100	India	1 month	ACS	Not mentioned	Not mentioned	Atorvastatin (40), Rosuvastatin (20)	Different statin types
J-STARS, (65)	1,565	Japan	4.9 years	Non-cardioembolic ischemic stroke	66	69	Pravastatin (10)	No treatment
Liu et al., (66)	591	China	1 year	ACS + PCI	62	49	Atorvastatin (20/40)	Different statin doses
Priti et al., (67)	1,027	India	1 month	STEMI	57	74	Atorvastatin (10/80)	Different statin doses
ACTIVE, (68)	173	United States, Canada	1 year	CABG	69	82	Atorvastatin (10/80)	Different statin doses
Liu et al., (69)	265	China	1 year	STEMI + PCI	59	72	Atorvastatin (20/40)	Different statin doses
Wang et al., (70)	162	China	1 year	MI	57	72	Atorvastatin (20)	No treatment
Kim et al., (71)	249	South Korea	3 months	CHD, PAD, TIA, stroke	63	81	Atorvastatin (10/20)	Different statin doses

ACS, acute coronary syndrome; AICAS, atherosclerotic intracranial arterial stenosis; ASCVD, atherosclerotic cardiovascular disease; CABG, coronary artery bypass graft; CHD, coronary heart disease; MCA, middle cerebral artery; MI, myocardial infarction; NSTEACS, non-ST-elevation acute coronary syndrome; PAD, peripheral artery disease; PCI, percutaneous coronary intervention; PTCA, Percutaneous transluminal coronary angioplasty; STEMI, ST segment elevation myocardial infarction; TIA, transient ischemic attack.

### Risk of bias and quality of evidence

**FIGURE 2:**
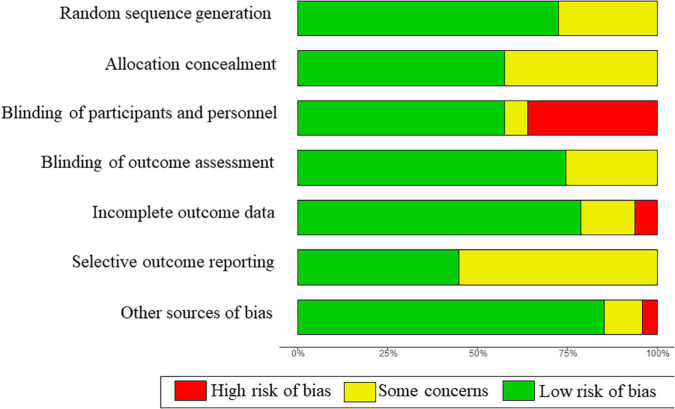
Summary of risk of bias across all included studies.

The overall risk of bias was rated as low or unclear in most studies (Figure 2). Twenty-six trials were double-blinded, and three did not state the blinding of participants and personnel. Seventeen trials were graded high risk regarding blind owing to the open-label design. The risk of bias in individual studies was described in Supplementary Table 4.

In pairwise meta-analyses, the quality of evidence for comparisons between statins and non-statin controls for cancer, MI, stroke, death from CVD, and all-cause death was rated as high, with evidence for the muscle condition, transaminase elevations, and gastrointestinal discomfort rated as moderate (Supplementary Table 5). In network meta-analyses, the quality of evidence for significant results was rated as high or moderate for transaminase elevations, and low for cancer (Supplementary Table 6).

### Results from systematic reviews

The rare number of studies prevented us from performing meta-analyses for the outcomes of new-onset or exacerbation of diabetes, cognitive impairment, and eye conditions. Only three of the included studies reported the new onset or exacerbation of diabetes. The Oxford Cholesterol study (26) reported 0, 1, 0 case of instability of control of diabetes in the simvastatin 40 mg (*n* = 206), 20 mg (*n* = 208) and placebo group (*n* = 207), respectively. The ATHEROMA study (44) documented 9 and 5 cases in the pravastatin group (*n* = 182) and no-treatment group (*n* = 179), respectively. Only the SEARCH study (58) reported the new-onset diabetes, of which 625 and 587 cases occurred in 80 mg (*n* = 6,031) and 20 mg (*n* = 6,033) simvastatin group, respectively. Only the HPS study (36) reported 2,434 and 2,484 cases of cognitive impairments in the simvastatin group (*n* = 10,269) and the placebo group (*n* = 10,267), respectively. Only two studies reported the eye conditions. The J-STARS study (64) reported 9 and 7 cases of colon polyp in the pravastatin group (*n* = 780) and no-treatment group (*n* = 785), respectively. The Oxford Cholesterol study (26) reported 2 and 0 cases of visual deterioration or eye-watering in the simvastatin group (*n* = 414) and placebo group (*n* = 207), respectively.

### Pairwise meta-analyses for primary and secondary outcomes

**FIGURE 3:**
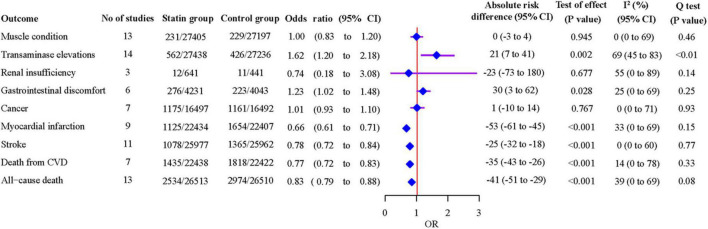
Associations of statins with safety and efficacy outcomes from pairwise meta-analyses.

Eighteen studies that compared statins with non-statin controls were included in pairwise meta-analyses. We found no significant heterogeneity between individual studies and used a fixed-effects model for most outcomes, except for transaminase elevations and renal insufficiency where random-effects models were adopted due to the significant heterogeneity (*P* < 0.01; *I*^2^ = 69%; 95% CI 45% to 83%, and *P* = 0.14; *I*^2^ = 55%; 95% CI 0% to 89%, respectively) (Figure 3 and Supplementary Figure 1).

As shown in Figure 3, statins were associated with an increased risk of transaminase elevations [14 studies, OR 1.62 (95% CI 1.20 to 2.18), *P* = 0.002], and gastrointestinal discomfort [6 studies, OR 1.23 (95% CI 1.02 to 1.48), *P* = 0.028]. Statins decreased the risk of MI [9 studies, OR 0.66 (95% CI 0.61 to 0.71), *P* < 0.001], stroke [11 studies, OR 0.78 (95% CI 0.72 to 0.84), *P* < 0.001], death from CVD [7 studies, OR 0.77 (95% CI 0.72 to 0.83), *P* < 0.001] and all-cause death [13 studies, OR 0.83 (95% CI 0.79 to 0.88), *P* < 0.001]. The leave-one-out influence analyses showed that the associations between statins and muscle condition, transaminase elevations, renal insufficiency, cancer, MI, stroke, death from CVD and all-cause death were not determined by any individual study (Supplementary Figure 2). The association with gastrointestinal discomfort was determined by the SPARCL trial (45), which reported diarrhea.

Compared with non-statin control, statins were estimated to induce 21 (7–41) more events of transaminase elevations, 30 (3–62) more events of gastrointestinal discomfort per 10,000 patients treated for a year (Figure 3). On the other hand, statins were estimated to prevent 53 (45–61) myocardial infarctions, 25 (18–32) strokes, 35 (26–43) deaths from CVD, and 41 (29–51) all-cause deaths per 10,000 patients treated for a year. The event rate per 10,000 patients throughout the duration of the studies is shown in Table 2. The absolute excess risk of the observed adverse effects of statins is smaller than the beneficial effects of statins on major cardiovascular events and all-cause death.

**TABLE 2:** Estimated maximum adverse effects of individual statins from Emax dose-response models*.

Statin	Muscle condition	Transaminase elevations	Renal insufficiency	Gastrointestinal discomfort	Cancer
Atorvastatin	1.02 (0.58 to 1.88)	19.72 (5.54 to 164.95)	0.87 (0.28 to 3.81)	1.34 (0.82 to 2.94)	0.65 (0.25 to 2.80)
Fluvastatin	1.21 (0.45 to 4.71)	3.70 (1.15 to 669.11)	/	1.26 (0.52 to 3.14)	0.92 (0.15 to 3.60)
Lovastatin	/	1.48 (0.24 to 640.74)	/	/	1.09 (0.30 to 2.72)
Pravastatin	0.85 (0.33 to 1.58)	1.40 (0.82 to 19,174.20)	/	1.49 (0.62 to 3.89)	1.09 (0.38 to 4.44)
Pitavastatin	/	3.57 (0.63 to 1,314.21)	/	/	1.29 (0.37 to 5.58)
Rosuvastatin	0.93 (0.38 to 2.31)	2.80 (0.98 to 287.30)	0.86 (0.20 to 3.46)	1.21 (0.35 to 6.09)	/
Simvastatin	/	7.42 (0.85 to 1,671.29)	0.93 (0.32 to 3.76)	0.41 (0.22 to 0.89)	0.98 (0.07 to 2.03)

Emax, asymptotic maximum drug effect. *The maximum odds ratio (OR_max_) with 95% credible interval (CI) in each cell is the maximum effect of each statin on the adverse event compared with non-statin controls (that is, the dose of the statin is 0), which is the natural exponential form of the estimated parameter, Emax, in each model.

We did not detect significant publication bias in each outcome (Supplementary Figure 3). In sensitivity analyses, the results were not influenced after excluding non-double-blind studies, Asian populations, or studies and individuals that did not reach a threefold elevation for transaminase (Supplementary Table 7). The results were also unchanged using the random-effects model, except for gastrointestinal discomfort [OR 1.07 (95% CI 0.73–1.57), *P* = 0.724].

### Results from network meta-analyses

Thirty-five studies were included in the networks of treatment comparisons, but due to the inconsistency between direct and indirect treatment comparisons in analysis for muscle conditions, only direct comparisons were performed for this outcome. The evidence network plots are shown in Supplementary Figure 4. We found no significant inconsistencies between direct and indirect treatment comparisons in other safety outcomes analyses (Supplementary Table 8).

**FIGURE 4:**
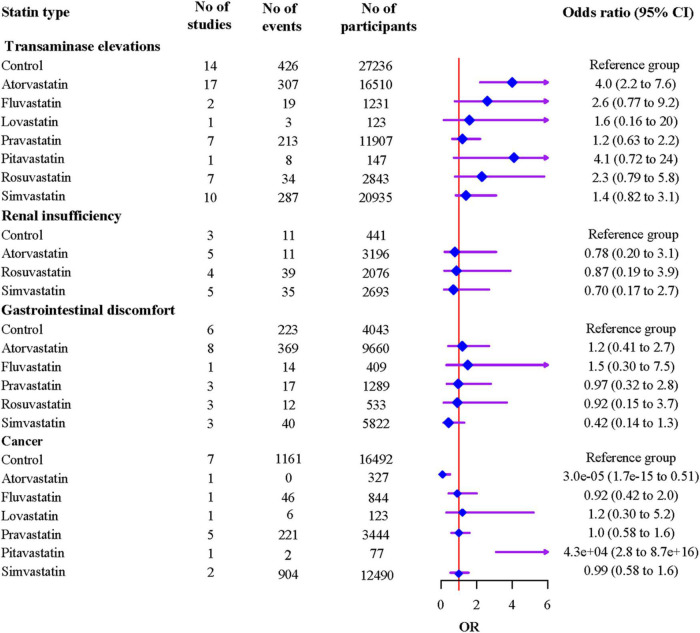
Associations of individual statins with adverse events from network meta-analyses.

The results indicated that compared to atorvastatin, simvastatin was associated with a lower risk of muscle problems [OR 0.70 (95% CI 0.55 to 0.90)], while rosuvastatin showed a higher risk [OR 1.75 (95% CI 1.17 to 2.61)] (Supplementary Table 9). Compared to the non-statin control, atorvastatin was associated with an increased risk of transaminase elevations [OR 4.0 (95% CI 2.2 to 7.6)] (Figure 4). Atorvastatin showed a higher risk of transaminase elevations than pravastatin [OR 3.49 (95% CI 1.77 to 6.92)] and simvastatin [OR 2.77 (95% CI 1.31 to 5.09)]. Atorvastatin showed a lower risk, and pitavastatin showed a higher risk of cancer than other statins and controls with wide 95% CIs, which indicated poor precision, because each of the two statins was reported in only one study (Figure 4 and Supplementary Table 10); this result should be treated with caution.

The ranking probabilities and cumulative probabilities plots of different statin types were shown in Supplementary Table 11 and Supplementary Figure 5, respectively. The ranking results based on SUCRA values showed that control had the highest rank for transaminase elevations (87.2%), and followed by pravastatin (76.3%), simvastatin (62.0%), lovastatin (56.2%), rosuvastatin (43.0%), fluvastatin (37.2%), pitavastatin (22.8%), and atorvastatin (15.3%); Simvastatin had the highest rank for renal insufficiency (65.9%), and followed by atorvastatin (54.8%), rosuvastatin (43.3%), and control (36.1%); Simvastatin had the highest rank for gastrointestinal discomfort (91.4%), and followed by rosuvastatin (52.7%), pravastatin (48.7%), control (48.2%), atorvastatin (32.8%), and fluvastatin (26.1%). Atorvastatin had the highest rank for cancer (99.0%), and followed by fluvastatin (58.7%), simvastatin (52.3%), control (51.6%), pravastatin (45.9%), lovastatin (41.7%), pitavastatin (0.7%).

### Dose-response relationships in adverse effects of statins

Forty-three studies were included in the dose-response meta-analyses. A significant Emax dose-response relationship was detected for the effect of atorvastatin and fluvastatin on transaminase elevations, with a maximum effect that increased the risk with non-statin controls [maximum OR (OR_*max*_) = 19.72, 95% CI (5.54 to 164.95); OR_*max*_ = 3.70, 95% CI (1.15 to 669.11), respectively] (Table 2). We detected no significant dose-response relationships for other statins on adverse effects.

The ranking probabilities in the dose-response meta-analysis showed similar results with network meta-analysis (Supplementary Table 12 and Supplementary Figure 6). The predicted dose-response curves, which had low precision with wide 95% CIs, were available for few doses of some statins, and only atorvastatin showed a precise shape (Supplementary Figure 7).

## Discussion

The salient findings of the meta-analyses of 47 RCTs, including 107,752 patients, can be summarized as follows. First, pairwise meta-analyses show that statins were associated with a higher incidence of transaminase elevations, but not with muscle condition, gastrointestinal discomfort, renal insufficiency and cancer. As expected, the benefit-to-harm balance of statins for secondary prevention of CVD is still favorable. Second, network meta-analyses indicated that atorvastatin could increase the risk of transaminase elevations compared to control, pravastatin and simvastatin. Compared to atorvastatin, simvastatin was associated with a lower risk of muscle problems, and rosuvastatin showed a higher risk. Third, a significant dose-response relationship was identified for the effect of atorvastatin on transaminase elevations. The dose-response relationships for the other statins and adverse effects were inconclusive.

Although statin-associated muscle symptoms (SAMS) have been reported, occurring in 7–29% of statin-treated patients and covering a wide range of severities (9), the mechanism remains unclear and whether SAMS is caused by statin use remained controversial. Our study did not find a significant association between statin and muscle condition and consistent with our findings. Many previous systematic reviews examining statins did not find an association between statins and muscle problems in the primary and secondary prevention population (4, 72–74). In contrast, some reviews showed associations between statins and increased risk of muscle symptoms and muscle diseases in the primary and general populations (75, 76). Nevertheless, we can find that the increased risk is slight in these reviews. These conflicting results may be attributed to the different populations and a wide range of conditions with varying types and severities of muscle problems. Some research indicated that most muscle symptoms reported by users of statins were due to “nocebo” effects rather than statins (75, 77). A recent meta-analysis of 176 studies with 4,143,517 patients showed that the prevalence of complete statin intolerance, mainly including SAMS, might often be overestimated (78). Previous reviews were underpowered for clinically confirmed muscle disorders to detect the associations between statins and myopathy or rhabdomyolysis because of the low incidences (75, 79, 80). Based on the current evidence, statins tend to have little effect on muscle problems in primary and secondary prevention.

The serum AST and ALT are commonly used tests to assess liver diseases. Our findings that statins use was associated with elevated transaminase were consistent with previous reviews, especially atorvastatin which was associated with a four times higher risk of transaminase elevation than non-statin controls. Furthermore, a significant Emax dose-response relationship was detected for the effect of atorvastatin on transaminase elevation. This adverse effect was similar in primary and secondary prevention (74, 80). A recent meta-analysis indicated that atorvastatin had the highest and dose-dependent risk of elevated transaminase (81). Another meta-analysis stated that compared to non-statin controls, patients treated with high dose atorvastatin (80 mg/day) had a higher risk of transaminase elevation, specifically in patients with CHD (82). Some clinical trials showed acceptable safety profiles of atorvastatin (83, 84), probably due to the small population scales of the studies and rare incidence rate of transaminase elevation. Atorvastatin is one of the most commonly prescribed drugs in the United States, with more than 50 million prescriptions per year (85). Atorvastatin is significantly longer-acting than other statins and is primarily metabolized in liver, which may explain the higher risk of liver dysfunction (86, 87). Dujovne (88) hypothesized that atorvastatin had more pronounced activity in reducing serum low-density lipoprotein, which may affect cell membrane structure, resulting in greater leakage of cellular enzymes and increased incidence of liver dysfunction. According to the current evidence, clinicians should avoid using atorvastatin when transaminase elevation occurs, especially high-dose atorvastatin, and pravastatin may be a better choice.

Studies about the effect of statins on renal function are contradictory. Some reviews, which included studies in secondary prevention, showed that statins reduce the progression of kidney function decline and proteinuria (89, 90). The latest The Kidney Disease: Improving Global Outcomes (KDIGO) guideline and 2016 ESC/EAS guidelines both recommend the use of statins in all non-dialysis dependent chronic kidney diseases patients ≥ 50 years with an eGFR below 60 mL/min/1.73 m^2^ or at least 30 mg/g albuminuria (91, 92). In contrast, some reviews showed associations between statins and renal insufficiency in primary prevention (75, 89). We found in the present study no significant associations between any statins and renal insufficiency. Nevertheless, the current data that can be analyzed is limited, and there is no convincing indication that any statin at any currently marketed dose causes renal disease.

There is no consensus on whether statins have a causal relationship with common gastrointestinal conditions. Studies indicated that statins tended to increase the risk of gastrointestinal discomfort or hemorrhage than other chronic medication users (93–95). Conversely, some studies did not show significant associations (96). The inconsistencies may result from varieties symptoms and severities of gastrointestinal discomfort.

The current evidence on the link between statins and cancer is conflicting. Consistent with our findings of pairwise meta-analyses, numerous systematic reviews showed no association between statin use and cancer incidence (97–99). Conversely, some studies showed that statins could affect the risk or development of cancers. On one hand, some evidence shows that statin therapy may increase the risk of some cancer types (100, 101). The PROSPER study (102) found a 1.25 increased risk for cancer incidence for the statin-treated patients compared to the placebo group; however, the authors have extended their follow-up period by 10 years and found no increased risk of cancer incidence for participants treated with statins compared to placebo. On the other hand, various *in vitro* and *in vivo* studies have revealed the efficacy of statins against cancers (103, 104). The inconsistency in published studies regarding statin use and cancer prevalence or mortality may be due to marked differences in follow-up duration, as well as other inherent biases in different study designs. Scholars reviewed the current contradictory evidence and believed that there was no increased risk of incident cancer with statin treatment (105).

Trial data on diabetes, cognitive impairments and eye conditions are currently limited, and no significant result was found in our systematic review. The rare records also reflect a low incidence rate from the side. Though a few studies suggested possible relationships between statin use and these adverse events (106, 107), the results were contradictory as some studies showed no associations (75, 107). More research data are needed to draw convincing conclusions.

The present study has several strengths. First, to our best knowledge, this is the first study to comprehensively explore the association between different types and doses of statins and adverse events in secondary prevention. Second, we include only RCTs which are more likely to provide unbiased information. Third, we performed network meta-analyses to establish multiple treatments comparison and synthesize data with not only direct evidence but also indirect evidence. Fourth, we also used Emax model, a newly developed and reliable method to examine the dose-response relationship of adverse reactions of statins. Compared with other models, this model reflects the basic Emax pharmacodynamics of common inhibitors with clinically interpretable parameters (108). Our study also provides the ranking probabilities of interventions that may provide some references for clinicians to make optimal decisions. Finally, we evaluated the absolute risk difference in the number of events per 10,000 patients treated for a year, which indicated that the benefit-to-harm balance of statins was favorable. Though intolerance to statins was considered as one of the main causes of insufficient LDL-C response to statin treatment (109), our findings add strong evidence to the current view that the cardiovascular benefits of statins far outweigh non-cardiovascular harms in patients with cardiovascular risk (110).

While this study does provide helpful information for clinicians, several limitations should be noted. The first point is the inconsistent definition of outcome measures. As mentioned earlier, the muscle condition, renal insufficiency and gastrointestinal discomfort are not specific and involve various symptoms. We have emphasized this limitation in the GRADE profile for authors to evaluate the quality of evidence. Second, although atorvastatin and pitavastatin both show differences from other statins and controls for the cancer incidence, this result is affected by the rare incidence of adverse events and limited sample sizes. Therefore, the result should be treated with caution. Also, due to the insufficient data, a few analyses were underpowered to detect differences between groups, and estimates of Emax from the models in this study made it difficult to draw more specific conclusions about the dose-response relationships. Third, the node-splitting analysis showed inconsistency in network meta-analysis of muscle condition. Though we excluded the studies that caused inconsistency, the results still need to be interpreted with caution. Fourth, a few studies were open-labeled and may induce bias, although sensitivity analyses showed that excluding these trials does not influence the overall results. Finally, due to data limitations, we could not further analyze the association of statins with the severity of adverse effects. Nevertheless, none of these limitations affects the main conclusion of our analysis.

## Conclusion

Statins were not associated with muscle condition, gastrointestinal discomfort, renal insufficiency and cancer but with increased risk of transaminases elevations in secondary prevention of ACSVD. Our study provides the ranking probabilities of statins that can help clinicians make optimal decisions when there is not enough literature to refer to. Future studies should systematically and detailly report adverse events of statins.

## Data availability statement

The original contributions presented in this study are included in the article/Supplementary material, further inquiries can be directed to the corresponding author/s.

## Author contributions

XW, HX, and KC: conceptualization. XW and JL: data curation and writing—original draft. XW, TW, and ZZ: formal analysis. QL and DM: methodology. HX and KC: project administration and supervision. ZC, JJ, and HX: writing—review and editing. All authors contributed to the article and approved the submitted version.
