# Supplementary material for: Associations between statins and adverse events in secondary prevention of cardiovascular disease: Pairwise, network, and dose-response meta-analyses of 47 randomized controlled trials
Source: Front Cardiovasc Med. 2022 Aug 25;9:929020. doi: 10.3389/fcvm.2022.929020 (PMC9452733; doi:10.3389/fcvm.2022.929020)
Supplement: Supplementary file 1 [file Data_Sheet_1.doc]

**Associations between statins and adverse events in secondary prevention of cardiovascular disease: pairwise, network, and dose-response meta-analyses of 47 randomised controlled trials**

**Contents**

**Table S1.** Search strategies in electronic bibliography databases

**Table S2.** Eligibility criteria of included studies

**Table S3.** List of studies excluded at full text review stage and reasons for exclusion

**Table S4.** Risk of bias in individual studies

**Table S5.** GRADE profile for pairwise meta-analyses

**Table S6.** GRADE profile for significant results from network meta-analyses

**Figure S1.** Forest plots of pair-wise meta-analyses (including results from fixed and random effects models)

**Figure S2.** Leave-one-out influence analyses for pair-wise meta-analyses

**Figure S3.** Funnel plots of publication bias in pair-wise meta-analyses

**Table S7.** Sensitivity analyses for pair-wise meta-analyses

**Figure S4.** Networks of treatment comparisons in network meta-analyses

**Table S8.** Node-splitting analyses of inconsistency between direct and indirect evidences in network meta-analyses

**Table S9.** Results of direct comparisons of different interventions for muscle condition

**Table S10.** Comparative adverse effects between different statin types from random-effect network meta-analyses

**Table S11.** Rank probabilities of different statin types in network meta-analyses

**Figure S5.** Cumulative ranking plot of different types of statins in network meta-analysis

**Table S12.** The SUCRA results in the dose-response meta-analyses

**Figure S6.** Cumulative ranking plot of Emax and ED50 parameters for different statins

**Figure S7.** E_max_ dose-response curves with dose-specific adverse effects of individual statins

**Table S1. Search strategies in electronic bibliography databases**

| **MEDLINE/PubMed** |
| --- |
| **1** hydroxymethylglutaryl coa inhibitors[MeSH Terms]  **2** statin[Title/Abstract] OR statins[Title/Abstract] OR atorvastatin[Title/Abstract] OR fluvastatin[Title/Abstract] OR lovastatin[Title/Abstract] OR pitavastatin[Title/Abstract] OR pravastatin[Title/Abstract] OR rosuvastatin[Title/Abstract] OR simvastatin[Title/Abstract]  **3** lipitor[Title/Abstract] OR lescol[Title/Abstract] OR mevacor[Title/Abstract] OR livalo[Title/Abstract] OR pravachol[Title/Abstract] OR crestor[Title/Abstract] OR zocor[Title/Abstract]  **4** #1 OR #2 OR #3  **5** randomized controlled trial[Publication Type] OR controlled clinical trial[Publication Type]  **6** randomized controlled trial[MeSH Terms] OR controlled clinical trials as topic[MeSH Terms]  **7** trial[Title/Abstract] AND random*[Title/Abstract] OR control*[Title/Abstract] OR palcebo[Title/Abstract]  **8** #5 OR #6 OR #7  **9** animals[MeSH Terms] NOT humans[MeSH Terms]  **10** #8 NOT #9  **11** #4 AND #10  **12** niacin[Title/Abstract] OR niaspan[Title/Abstract] OR bile acid[Title/Abstract] OR cholestyramine[Title/Abstract] OR colesevelam[Title/Abstract] OR colestipol[Title/Abstract] OR ezetimibe[Title/Abstract] OR fibrate[Title/Abstract] OR fibrates[Title/Abstract] OR fenofibrate[Title/Abstract] OR gemfibrozil[Title/Abstract] OR PCSK9[Title/Abstract] OR alirocumab[Title/Abstract] OR evolocumab[Title/Abstract]  **13** #11 NOT #12  **14** comment[Publication Type] OR congress[Publication Type] OR duplicate publication[Publication Type] OR editorial[Publication Type] OR letter[Publication Type] OR meta analysis[Publication Type] OR news[Publication Type] OR published erratum[Publication Type] OR review[Publication Type] OR systematic review[Publication Type]  **15** protocol[Title]  **16** #14 OR #15  **17** #13 NOT #16  **18** coronary[Title/Abstract] OR ischemic[Title/Abstract] OR atherosclerotic[Title/Abstract] OR cardiovascular[Title/Abstract] OR heart[Title/Abstract]  **19** disease*[Title/Abstract] OR syndrome*[Title/Abstract] OR condition*[Title/Abstract] OR stenosis[Title/Abstract]  **20** angina pectoris[Title/Abstract] OR angina pectoris[MeSH Terms]  **21** myocardial ischemia[MeSH Terms] OR myocardial ischemia[Title/Abstract]  **22** myocardial infarct*[Title/Abstract] OR myocardial infarction[MeSH Terms]  **23** acute coronary syndrome*[Title/Abstract] OR acute coronary syndrome[MeSH Terms]  **24** #18 OR #19 OR #20 OR #21 OR #22 OR #23  **25** #17 AND #24 |
| **Embase** |
| **#1** statin?:ab,ti OR atorvastatin:ab,ti OR fluvastatin:ab,ti OR lovastatin:ab,ti OR pitavastatin:ab,ti OR pravastatin:ab,ti OR simvastatin:ab,ti OR rosuvastatin:ab,ti  **#2** 'hydroxymethylglutaryl coenzyme a reductase inhibitor'/exp  **#3** lipitor:ab,ti OR lescol:ab,ti OR mevacor:ab,ti OR livalo:ab,ti OR pravachol:ab,ti OR crestor:ab,ti OR zocor:ab,ti  **#4** #1 OR #2 OR #3  **#5** #4 AND ('controlled clinical trial'/de OR 'randomized controlled trial'/de)  **#6** 'animals'/exp NOT 'humans'/exp  **#7** #5 NOT #6  **#8** niacin:ab,ti OR niaspan:ab,ti OR 'bile acid':ab,ti OR cholestyramine:ab,ti OR colesevelam:ab,ti OR colestipol:ab,ti OR ezetimibe:ab,ti OR fibrate?:ab,ti OR fenofibrate:ab,ti OR gemfibrozil:ab,ti OR pcsk9:ab,ti OR alirocumab:ab,ti OR evolocumab:ab,ti OR 'n-3 polyunsaturated fatty acid':ab,ti OR fenofibric:ab,ti OR coenzyme:ab,ti OR 'omega-3 fatty acids'  **#9** #7 NOT #8  **#10** acute AND coronary AND syndrome  **#11** myocardial AND infarction  **#12** angina AND pectoris  **#13** coronary AND ('artery'/exp OR artery) AND ('disease'/exp OR disease)  **#14** #10 OR #11 OR #12 OR #13  **#15** #9 AND #14 |
| **CENTRAL (Cochrane Library)** |
| **#1** (statin):ti OR (statins):ti OR (atorvastatin) :ti OR (fluvastatin):ti OR (lovastatin):ti OR (pitavastatin):ti OR (pravastatin):ti OR (simvastatin):ti OR (rousuvastatin):ti  **#2** (lipitor):ti OR (lescol):ti OR (mevacor):ti OR (livalo):ti OR (pravachol):ti OR (crestor):ti OR (zocor)  **#3** MeSH descriptor: [hydroxymethylglutaryl-CoA Reductase inhibitors] explode all trees  **#4** #1 OR #2 OR #3  **#5 (**niacin):ti,ab,kw OR (niaspan):ti,ab,kw OR (bile acid):ti,ab,kw OR (cholestyramine):ti,ab,kw OR (colesevelam):ti,ab,kw OR (colestipol):ti,ab,kw OR (ezetimibe):ti,ab,kw OR (fibrate):ti,ab,kw OR (fibrates):ti,ab,kw OR (fenofibrate):ti,ab,kw OR (gemfibrozil):ti,ab,kw OR (PCSK9):ti,ab,kw OR (alirocumab):ti,ab,kw OR (evolocumab):ti,ab,kw):ti,ab,kw OR (coenzyme):ti,ab,kw OR (N-3 polyunsaturated fatty acid):ti,ab,kw OR (omega-3 fatty acids):ti,ab,kw OR (fenofibric)  **#6** #4 NOT #5  **#7** MeSH descriptor: [Coronary Artery Disease] explode all trees  **#8** MeSH descriptor: [Myocardial Ischemia] explode all trees  **#9** MeSH descriptor: [Angina pectoris] explode all trees  **#10** MeSH descriptor: [Stroke] explode all trees  **#11** (coronary):ti,ab,kw OR (ischemic):ti,ab,kw OR (atherosclerotic):ti,ab,kw OR (cardiovascular):ti,ab,kw OR (heart):ti,ab,kw  **#12 (CAD**):ti,ab,kw OR (ACS):ti,ab,kw OR (NSTEMI):ti,ab,kw  **#13** #7 OR #8 OR #9 OR #10 OR #11 OR #12  **#14 #6 AND #13** |

*** All searches were updated by 16 October 2021.**

**Table S2. Eligibility criteria of included studies**

|  | **Inclusion Criteria** | **Exclusion Criteria** |
| --- | --- | --- |
| **Study design/ Settings** | Randomized controlled clinical trial | (1) Cross-over or self-controlled study designs;  (2) Re-analysis of existing trials (e.g. post hoc analysis /subgroup analysis);  (3) Examination of the pleiotropy of statins for non-CVD conditions;  (4) Intervention duration < four weeks |
| **Participants** | (1) Secondary-prevention patients (defined by a history of known ASCVD, i.e. coronary heart disease, peripheral artery disease, or cerebrovascular disease)  (2) If a small proportion of non-ASCVD patients was involved, yje proportion should be > 60% | Number of participants < 100 |
| **Interventions** | (1) Any of the seven types of statins in clinical use, including Atorvastatin, Fluvastatin, Lovastatin, Pitavastatin, Pravastatin, Rosuvastatin, and Simvastatin;  (2) No limitation on doses or formulations (e.g. oral, topical) | (1) Statins were used in combination with other lipid-lowering medications;  (2) Multiple types of statins were used in the same group;  (3) Statins were used in the run-in phase |
| **Comparators** | (1) Non-statin controls (e.g. placebo, routine care, dietary management, no treatment);  (2) Different types or doses of statins |  |
| **Outcomes** | Primary outcomes:  (1) Muscle condition (including self-reported muscle symptoms, clinically-confirmed muscle disorders);  (2) Transaminase elevations;  (3) Renal insufficiency;  (4) Gastrointestinal discomfort;  (5) Cancer;  (6) New onset or exacerbation of diabetes;  (7) Cognitive impairment;  (8) Eye condition;  Secondary outcomes:  (1) Myocardial infarction;  (2) Stroke;  (3) Death from cardiovascular diseases;  (4) All-cause death | No desired outcome data available, either effect estimate with standard error for compared groups or number/rate of events in each group |

**Table S3. List of studies excluded at full text review stage and reasons for exclusion**

| **ID** | **Citation** | **Reason for exclusion** |
| --- | --- | --- |
| **Studies identified from previous reviews** | | |
| 1 | Shepherd J, Cobbe SM, Ford I, et al. Prevention of coronary heart disease with pravastatin in men with hypercholesterolemia [J]. N Engl Med. 1995;333:1301-7. | Not ASCVD populations |
| 2 | Downs JR, Clearfield M, Weis S, et al. Primary prevention of acute coronary events with lovastatin in men and women with average cholesterol levels: results of AFCAPS/TexCAPS [J]. JAMA. 1998;279:1615-22. |
| 3 | Wenger NK, Lewis SJ, Herrington DM, et al. Outcomes of using high- or low-dose atorvastatin in patients 65 years of age or older with stable coronary heart disease [J]. Ann Intern Med, 2007,147(1):1-9. | Re-analysis of existing trials |
| 4 | Koren MJ, Feldman T, Mendes RA. Impact of high-dose atorvastatin in coronary heart disease patients age 65 to 78 years [J]. Clin Cardiol. 2009,32(5):256-63. |
| 5 | Suh JW, Cha MJ, Lee SP, et al. Relationship between statin type and responsiveness to clopidogrel in patients treated with percutaneous coronary intervention: a subgroup analysis of the CILON-T trial [J]. J Atheroscler Thromb, 2014, 21:140-50 |
| 6 | Athyros VG, Tziomalos K, Gossios TD, et al. Safety and efficacy of long-term statin treatment for cardiovascular events in patients with coronary heart disease and abnormal liver tests in the Greek Atorvastatin and Coronary Heart Disease Evaluation (GREACE) Study: a post-hoc analysis [J]. Lancet, 2010, 376(9756):1916-22. |
| 7 | Lemos JA, Blazing MA, Wiviott SD, et al. Early intensive vs a delayed conservative simvastatin strategy in patients with acute coronary syndromes: phase Z of the A to Z trial [J]. JAMA, 2004,292(11):1307-16. | No report on the outcomes of interest/No specific outcome data |
| 8 | Stone PH, Lloyd-Jones DM, Kinlay S, et al. Effect of intensive lipid lowering, with or without antioxidant vitamins, compared with moderate lipid lowering on myocardial ischemia in patients with stable coronary artery disease: the Vascular Basis for the Treatment of Myocardial Ischemia Study [J]. Circulation, 2005, 111(14):1747-55. |
| 9 | Im E, Cho YH, Suh Y, et al. High-intensity Statin Treatments in Clinically Stable Patients on Aspirin Monotherapy 12 Months After Drug-eluting Stent Implantation: A Randomized Study [J]. Rev Esp Cardiol (Engl Ed). 2018,71(6):423-31. |
| 10 | Schartl M, Bocksch W, Koschyk DH, et al. Use of intravascular ultrasound to compare effects of different strategies of lipid-lowering therapy on plaque volume and composition in patients with coronary artery disease [J]. Circulation, 2001, 104(4):387-92 |
| 11 | Teo KK, et al. Long-term effects of cholesterol lowering and angiotensin-converting enzyme inhibition on coronary atherosclerosis: The Simvastatin/Enalapril Coronary Atherosclerosis Trial (SCAT) [J]. Circulation, 2000, 102(15):1748-54. |
| 12 | Post Coronary Artery Bypass Graft Trial Investigators. The effect of aggressive lowering of low-density lipoprotein cholesterol levels and low-dose anticoagulation on obstructive changes in saphenous-vein coronary-artery bypass grafts [J]. N Engl J Med, 1997;336(3):153-62. | Combined with other lipid-lowering drugs |
| 13 | Shehata M, Fayez G, Nassar A. Intensive Statin Therapy in NSTE-ACS Patients Undergoing PCI: Clinical and Biochemical Effects [J]. ex Heart Inst J, 2015, 42(6):528-36. | Use statins in the run-in phase |
| 14 | Colivicchi F, Guido V, Tubaro M, et al. Effects of atorvastatin 80 mg daily early after onset of unstable angina pectoris or non-Q-wave myocardial infarction [J]. Am J Cardiol, 2002, 90(8):872-4. | Sample size <100 |
| 15 | Kesteloot H, Claeys G, Blanckaert N et al. Time course of serum lipids and apolipoproteins after acute myocardial infarction: modification by pravastatin [J]. Acta Cardiol, 1997, 52: 107-16. |
| 16 | Kayikçioğlu M, Can L, Kültürsay A, et al. Early use of pravastatin in patients with acute myocardial infarction undergoing coronary angioplasty [J]. Acta Cardiol, 2002, 57(4):295-302. |
| 17 | Dupuis J, Tardif JC, Rouleau JL, et al. Intensity of lipid lowering with statins and brachial artery vascular endothelium reactivity after acute coronary syndromes (from the BRAVER trial) [J]. Am J Cardiol, 2005, 96(9):1207-13. |
| 18 | Macin SM, Perna ER, Farías EF, et al. Atorvastatin has an important acute anti-inflammatory effect in patients with acute coronary syndrome: results of a randomized, double-blind, placebo-controlled study [J]. Am Heart J, 2005, 149(3):451-7. |
| 19 | Liu Z, Joerg H, Hao H, et al. Efficacy of High-Intensity Atorvastatin for Asian Patients Undergoing Percutaneous Coronary Intervention [J]. Ann Pharmacother. 2016, 50(9):725-33. | Statin type and dose not specified |
| 20 | Den Hartog FR, Van Kalmthout PM, Van Loenhout TT, et al. Pravastatin in acute ischaemic syndromes: results of a randomised placebo-controlled trial [J]. Int J Clin Pract, 2001, 55(5):300-4. | Intervention duration < 4 weeks |
| **Studies identified from database searches** | | |
| 1 | Laks T, Keba E, Leiner M, et al. Achieving lipid goals with rosuvastatin compared with simvastatin in high risk patients in real clinical practice: a randomized, open-label, parallel-group, multi-center study: the DISCOVERY-Beta study [J]. Vasc Health Risk Manag, 2008, 4: 1407-16. | ASCVD patietnts <60% |
| 2 | Taylor AJ, Kent SM, Flaherty PJ, et al. ARBITER: Arterial Biology for the Investigation of the Treatment Effects of Reducing Cholesterol: a randomized trial comparing the effects of atorvastatin and pravastatin on carotid intima medial thickness [J]. Circulation, 2002,106(16): 2055-60. |
| 3 | Gonbert S, Malinsky S, Sposito AC, et al. Atorvastatin lowers lipoprotein(a) but not apolipoprotein(a) fragment levels in hypercholesterolemic subjects at high cardiovascular risk [J]. Atherosclerosis, 2002,164: 305-11. |
| 4 | Arshad AR. Comparison of low-dose rosuvastatin with atorvastatin in lipid-lowering efficacy and safety in a high-risk Pakistani cohort: An open-label randomized trial [J]. J Lipids, 2014, 2014: 875907. |
| 5 | Deedwania PC, Gupta M, Stein M, et al. Comparison of rosuvastatin versus atorvastatin in South-Asian patients at risk of coronary heart disease (from the IRIS Trial).[J] .Am J Cardiol, 2007, 99: 1538-43. |
| 6 | Zhu JR, Tomlinson B, Ro YM, et al. A randomised study comparing the efficacy and safety of rosuvastatin with atorvastatin for achieving lipid goals in clinical practice in Asian patients at high risk of cardiovascular disease (DISCOVERY-Asia study) [J] .Curr Med Res Opin, 2007, 23: 3055-68. |
| 7 | Binbrek AS, Elis A, Al-Zaibag M, et al. Rosuvastatin versus atorvastatin in achieving lipid goals in patients at high risk for cardiovascular disease in clinical practice: A randomized, open-label, parallel-group, multicenter study (DISCOVERY Alpha study) [J] .Curr Ther Res Clin Exp, 2006, 67: 21-43. |
| 8 | Kim JW, Yun KH, Kim EK, et al. Effect of High Dose Rosuvastatin Loading before Primary Percutaneous Coronary Intervention on Infarct Size in Patients with ST-Segment Elevation Myocardial Infarction [J] .Korean Circ J, 2014, 44: 76-81. | Not RCT |
| 9 | Zbinden S, Brunner N, Wustmann K, et al. Effect of statin treatment on coronary collateral flow in patients with coronary artery disease [J]. Heart, 2004, 90: 448-9. |
| 10 | Amirov NB, Potapova MV, Ishkineev FI, et al. Dyslipidemia correction with atorvastatin in patients with coronary heart disease and arterial hypertension[J]. Cardiovascular Therapy & Prevention, 2007, 6(7):55-58. |
| 11 | Son JW, Koh KK, Ahn JY, et al. Effects of statin on plaque stability and thrombogenicity in hypercholesterolemic patients with coronary artery disease [J]. Int J Cardiol, 2003, 88: 77-82. |
| 12 | Clearfield M. Aggressive low-density lipoprotein cholesterol lowering in secondary prevention of coronary heart disease [J]. Curr Atheroscler Rep. 2006, 8(1):7-8. |
| 13 | Semenova AE, Sergienko IV, Masenko VP, et al. Effect of rosuvastatin therapy and myocardial revascularization on angiogenesis in coronary artery disease patients [J] .Kardiologiia, 2007, 47: 4-8. |
| 14 | Pelliccia F, Rosano G, Marazzi G, et al. Pharmacodynamic comparison of pitavastatin versus atorvastatin on platelet reactivity in patients with coronary artery disease treated with dual antiplatelet therapy [J] .Circ J, 2014, 78: 679-84. | cross over study |
| 15 | Riegger G, Abletshauser C, Ludwig M, et al. The effect of fluvastatin on cardiac events in patients with symptomatic coronary artery disease during one year of treatment.[J]. Atherosclerosis. 1999 May;144(1):263-70. | Statins were used in the run-in phase |
| 16 | Schmermund A, Achenbach S, Budde T, et al. Effect of intensive versus standard lipid-lowering treatment with atorvastatin on the progression of calcified coronary atherosclerosis over 12 months: a multicenter, randomized, double-blind trial.[J] .Circulation, 2006, 113: 427-37. |
| 17 | Nicholls S J, Ballantyne CM, Barter P J, et al. Effect of two intensive statin regimens on progression of coronary disease.[J] .N Engl J Med, 2011, 365: 2078-87. |
| 18 | Shehata M,Samir A,Dardiri M. Prognostic impact of intensive statin therapy on N-terminal pro-BNP level in non-ST-segment elevation acute myocardial infarction patients.[J] .J Interv Cardiol, 2017, 30: 514-521. |
| 19 | Kim S W,Bae K Y,Kim J M et al. The use of statins for the treatment of depression in patients with acute coronary syndrome.[J] .Transl Psychiatry, 2015, 5: e620. | Escitalopram vs placebo |
| 20 | Sakamoto T,Kojima S,Ogawa H et al. Effects of early statin treatment on symptomatic heart failure and ischemic events after acute myocardial infarction in Japanese.[J] .Am J Cardiol, 2006, 97: 1165-71. | Statin type and dose were not specified |
| 21 | Colivicchi F,Tubaro M,Mocini D et al. Full-dose atorvastatin versus conventional medical therapy after non-ST-elevation acute myocardial infarction in patients with advanced non-revascularisable coronary artery disease.[J] .Curr Med Res Opin, 2010, 26: 1277-84. |
| 22 | Zhang X,Wang H R,Liu Sh S et al. Intensive-dose atorvastatin regimen halts progression of atherosclerotic plaques in new-onset unstable angina with borderline vulnerable plaque lesions.[J] .J Cardiovasc Pharmacol Ther, 2013, 18: 119-25. |
| 23 | Spring S,Simon R,van der L B et al. High-dose atorvastatin in peripheral arterial disease (PAD): effect on endothelial function, intima-media-thickness and local progression of PAD. An open randomized controlled pilot trial.[J] .Thromb Haemost, 2008, 99: 182-9. |
| 24 | van der Loo B, Spring S, Koppensteiner R. High-dose atorvastatin treatment in patients with peripheral arterial disease: effects on platelet aggregation, blood rheology and plasma homocysteine.[J]. Clin Hemorheol Microcirc. 2011;47(4):241-51. |
| 25 | Xu H,Zou J,Ye X l et al. Impacts of Clinical Pharmacist Intervention on the Secondary Prevention of Coronary Heart Disease: A Randomized Controlled Clinical Study.[J] .Front Pharmacol, 2019, 10: 1112. |
| 26 | Tentzeris I,Rohla M,Jarai R et al. Influence of high-dose highly efficient statins on short-term mortality in patients undergoing percutaneous coronary intervention with stenting for acute coronary syndromes.[J] .Am J Cardiol, 2014, 113: 1099-104. |
| 27 | Collard C D,Body S C,Shernan S K et al. Preoperative statin therapy is associated with reduced cardiac mortality after coronary artery bypass graft surgery.[J] .J Thorac Cardiovasc Surg, 2006, 132: 392-400. |
| 28 | Chiodini BD, Franzosi MG, Barlera S et al. Apolipoprotein E polymorphisms influence effect of pravastatin on survival after myocardial infarction in a Mediterranean population: the GISSI-Prevenzione study[J]. Eur Heart J. 2007 Aug;28(16):1977-83. | Re-analysis of existing trials (post-trial data/subgroup analysis/additional non-interested outcomes) |
| 29 | Chaturvedi S, Zivin J, Breazna A et al. Effect of atorvastatin in elderly patients with a recent stroke or transient ischemic attack.[J]. Neurology. 2009 Feb 24;72(8):688-94. |
| 30 | Collins R,Armitage J,Parish S et al. Effects of cholesterol-lowering with simvastatin on stroke and other major vascular events in 20536 people with cerebrovascular disease or other high-risk conditions.[J] .Lancet, 2004, 363: 757-67. |
| 31 | MRC/BHF Heart Protection Study Collaborative Group, Armitage J, Bowman L et al. Effects of simvastatin 40 mg daily on muscle and liver adverse effects in a 5-year randomized placebo-controlled trial in 20,536 high-risk people.[J]. BMC Clin Pharmacol. 2009 Mar 31;9:6. |
| 32 | Hong Y J,Jeong M H,Bae J H et al. Efficacy and safety of pitavastatins in patients with acute myocardial infarction: Livalo in Acute Myocardial Infarction Study (LAMIS) II.[J] .Korean J Intern Med, 2017, 32: 656-667. |
| 33 | Lemos P A,de Feyter P J,Serruys P W et al. Fluvastatin reduces the 4-year cardiac risk in patients with multivessel disease.[J] .Int J Cardiol, 2005, 98: 479-86. |
| 34 | Koren M J,Davidson M H,Wilson D J et al. Focused atorvastatin therapy in managed-care patients with coronary heart disease and CKD.[J] .Am J Kidney Dis, 2009, 53: 741-50. |
| 35 | Dohi T,Miyauchi K,Okazaki S et al. Higher baseline LDL-C levels amplify the short-term benefit of early intensive statin treatment in acute coronary syndrome.[J] .J Atheroscler Thromb, 2011, 18: 42-8. |
| 36 | Kulik A,Abreu A M,Boronat V et al. Impact of lipid levels and high-intensity statins on vein graft patency after CABG: Midterm results of the ACTIVE trial.[J] .J Card Surg, 2020, 35: 3286-3293. |
| 37 | Briguori C, Quintavalle C, D'Alessio F et al. Impact of statin therapy intensity on endothelial progenitor cells after percutaneous coronary intervention in diabetic patients. The REMEDY-EPC late study.[J]. Int J Cardiol. 2017 Oct 1;244:112-118. |
| 38 | Ray K K,Cannon C P,Cairns R et al. Relationship between uncontrolled risk factors and C-reactive protein levels in patients receiving standard or intensive statin therapy for acute coronary syndromes in the PROVE IT-TIMI 22 trial.[J] .J Am Coll Cardiol, 2005, 46: 1417-24. |
| 39 | Herrington D M,Vittinghoff E,Lin F et al. Statin therapy, cardiovascular events, and total mortality in the Heart and Estrogen/Progestin Replacement Study (HERS).[J] .Circulation, 2002, 105: 2962-7. |
| 40 | Pyŏrälä K,Pedersen TR, Kjekshuset J, al. Cholesterol lowering with simvastatin improves prognosis of diabetic patients with coronary heart disease. A subgroup analysis of the Scandinavian Simvastatin Survival Study (4S) [J]. Diabetes Care, 1997, 20(4):614-20. |
| 41 | Liang D,Zhang Q,Yang H et al. Anti-oxidative stress effect of loading-dose rosuvastatin prior to percutaneous coronary intervention in patients with acute coronary syndrome: a prospective randomized controlled clinical trial.[J] .Clin Drug Investig, 2014, 34: 773-81. | Intervention duration < 4 weeks |
| 42 | Chen J H,Li M C,Zhu X et al. Atorvastatin reduces cerebral vasospasm and infarction after aneurysmal subarachnoid hemorrhage in elderly Chinese adults.[J] .Aging (Albany NY), 2020, 12: 2939-2951. |
| 43 | Pedersen T R,Jahnsen K E,Vatn S et al. Benefits of early lipid-lowering intervention in high-risk patients: the lipid intervention strategies for coronary patients study.[J] .Clin Ther, 2000, 22: 949-60. |
| 44 | Liu W B,Zou Z P,Jiang H P et al. Clinical effect of preoperative high-dose atorvastatin against no-reflow after PCI.[J] .Exp Ther Med, 2017, 13: 97-102. |
| 45 | Qiao L Y,Wang S B,Jia et al. Clinical efficacy and safety of statin treatment after carotid artery stenting.[J] .Artif Cells Nanomed Biotechnol, 2019, 47: 3110-3115. |
| 46 | Sardella G,Lucisano L,Mancone M et al. Comparison of high reloading ROsuvastatin and Atorvastatin pretreatment in patients undergoing elective PCI to reduce the incidence of MyocArdial periprocedural necrosis. The ROMA II trial.[J] .Int J Cardiol, 2013, 168: 3715-20. |
| 47 | Barbarash O,Gruzdeva O,Uchasova E et al. Dose-dependent effects of atorvastatin on myocardial infarction.[J] .Drug Des Devel Ther, 2015, 9: 3361-8. |
| 48 | Zhang D,Wang S Q,Guan Y Q et al. Effect of oral atorvastatin on CD4+CD25+ regulatory T cells, FoxP3 expression, and prognosis in patients with ST-segment elevated myocardial infarction before primary percutaneous coronary intervention.[J] .J Cardiovasc Pharmacol, 2011, 57: 536-41. |
| 49 | Ji Q,Mei Y Q,Wang X S et al. Effect of preoperative atorvastatin therapy on atrial fibrillation following off-pump coronary artery bypass grafting.[J] .Circ J, 2009, 73: 2244-9. |
| 50 | Mannacio V A,Iorio D,De A V et al. Effect of rosuvastatin pretreatment on myocardial damage after coronary surgery: a randomized trial.[J] .J Thorac Cardiovasc Surg, 2008, 136: 1541-8. |
| 51 | Song Y B,On Y K,Kim J H et al. The effects of atorvastatin on the occurrence of postoperative atrial fibrillation after off-pump coronary artery bypass grafting surgery.[J] .Am Heart J, 2008, 156: 373.e9-16. |
| 52 | Li Q,Zhao Y G,Wang Z et al. Effects of First High-Dose Atorvastatin Loading in Patients With ST-Segment Elevation Myocardial Infarction Undergoing Percutaneous Coronary Intervention.[J] .Am J Ther, 2018, 25: e291-e298. |
| 53 | Kleemann A,Eckert S,von Eckardstein A et al. Effects of lovastatin on progression of non-dilated and dilated coronary segments and on restenosis in patients after PTCA. The cholesterol lowering atherosclerosis PTCA trial (CLAPT).[J] .Eur Heart J, 1999, 20: 1393-406. |
| 54 | Ko Y G,Won H,Shin D H et al. Efficacy of early intensive rosuvastatin therapy in patients with ST-segment elevation myocardial infarction undergoing primary percutaneous coronary intervention (ROSEMARY Study).[J] .Am J Cardiol, 2014, 114: 29-35. |
| 55 | Kim J S,Kim J,Choi D et al. Efficacy of high-dose atorvastatin loading before primary percutaneous coronary intervention in ST-segment elevation myocardial infarction: the STATIN STEMI trial.[J] .JACC Cardiovasc Interv, 2010, 3: 332-9. |
| 56 | Jo S H,Hahn J Y,Lee S Y et al. High-dose atorvastatin for preventing contrast-induced nephropathy in primary percutaneous coronary intervention.[J] .J Cardiovasc Med (Hagerstown), 2015, 16: 213-9. |
| 57 | Taguchi I,Iimuro S,Iwata H et al. High-Dose Versus Low-Dose Pitavastatin in Japanese Patients With Stable Coronary Artery Disease (REAL-CAD): A Randomized Superiority Trial.[J] .Circulation, 2018, 137: 1997-2009. |
| 58 | Youn Y N,Park S Y,Hwang Y et al. Impact of High-Dose Statin Pretreatment in Patients with Stable Angina during Off-Pump Coronary Artery Bypass.[J] .Korean J Thorac Cardiovasc Surg, 2011, 44: 208-14. |
| 59 | Li Q, Deng SB, Xia S, Du JL et al. Impact of intensive statin use on the level of inflammation and platelet activation in stable angina after percutaneous coronary intervention: a clinical study.[J]. Med Clin (Barc). 2013 Jun 18;140(12):532-6. |
| 60 | Xu X R,Liu Y,Li K B et al. Intensive atorvastatin improves endothelial function and decreases ADP-induced platelet aggregation in patients with STEMI undergoing primary PCI: A single-center randomized controlled trial.[J] .Int J Cardiol, 2016, 222: 467-472. |
| 61 | Jia X W,Fu X H,Zhang J et al. Intensive cholesterol lowering with statin improves the outcomes of percutaneous coronary intervention in patients with acute coronary syndrome.[J] .Chin Med J (Engl), 2009, 122: 659-64. |
| 62 | Naraoka M,Matsuda N,Shimamura No et al. Long-acting statin for aneurysmal subarachnoid hemorrhage: A randomized, double-blind, placebo-controlled trial.[J] .J Cereb Blood Flow Metab, 2018, 38: 1190-1198. |
| 63 | Xia J G,Qu Y,Shen H et al. Patients with stable coronary artery disease receiving chronic statin treatment who are undergoing noncardiac emergency surgery benefit from acute atorvastatin reload.[J] .Cardiology, 2014, 128: 285-92. |
| 64 | Zheng Z,Jayaram R,Jiang L X et al. Perioperative Rosuvastatin in Cardiac Surgery.[J] .N Engl J Med, 2016, 374: 1744-53. |
| 65 | Godino C,Pavon A G,Mangieri A et al. Platelet reactivity in response to loading dose of atorvastatin or rosuvastatin in patients with stable coronary disease before percutaneous coronary intervention: The STATIPLAT randomized study.[J] .Clin Cardiol, 2017, 40: 605-611. |
| 66 | Xia J G,Qu Y,Yin C L et al. Preoperative rosuvastatin protects patients with coronary artery disease undergoing noncardiac surgery.[J] .Cardiology, 2015, 131: 30-7. |
| 67 | Pierri M D,Crescenzi G,Zingaro C et al. Prevention of atrial fibrillation and inflammatory response after on-pump coronary artery bypass using different statin dosages: a randomized, controlled trial.[J] .Gen Thorac Cardiovasc Surg, 2016, 64: 395-402. |
| 68 | Acikel S, Muderrisoglu H, Yildirir A et al. Prevention of contrast-induced impairment of renal function by short-term or long-term statin therapy in patients undergoing elective coronary angiography.[J]. Blood Coagul Fibrinolysis. 2010 Dec;21(8):750-7. |
| 69 | Pasceri V,Patti G,Nusca A et al. Randomized trial of atorvastatin for reduction of myocardial damage during coronary intervention: results from the ARMYDA (Atorvastatin for Reduction of MYocardial Damage during Angioplasty) study.[J] .Circulation, 2004, 110: 674-8. |
| 70 | Martins K B A,Mattos L A P,Sousa A G M R et al. A Randomized Trial of Creatine-kinase Leak After Rosuvastatin in Elective Percutaneous Coronary Intervention (CLEAR-PCI).[J] .J Interv Cardiol, 2015, 28: 339-47. |
| 71 | García-Méndez R C,Almeida-Gutierrez E,Serrano-Cuevas L et al. Reduction of No Reflow with a Loading Dose of Atorvastatin before Primary Angioplasty in Patients with Acute ST Myocardial Infarction.[J] .Arch Med Res, 2018, 49: 620-629. |
| 72 | Kaya A, Kurt M, Tanboğa IH et al. Rosuvastatin versus atorvastatin to prevent contrast induced nephropathy in patients undergoing primary percutaneous coronary intervention (ROSA-cIN trial).[J]. Acta Cardiol. 2013 Oct;68(5):489-94. |
| 73 | Kirkpatrick P J,Turner C L,Smith C et al. Simvastatin in aneurysmal subarachnoid haemorrhage (STASH): a multicentre randomised phase 3 trial.[J] .Lancet Neurol, 2014, 13: 666-75. |
| 74 | Takano H,Ohba T,Yamamoto E et al. Usefulness of rosuvastatin to prevent periprocedural myocardial injury in patients undergoing elective coronary intervention.[J] .Am J Cardiol, 2013, 111: 1688-93. |
| 75 | Wu H, Han Y L, Wang X Z et al. Effects of short-term rosuvastatin therapy on heart and kidney function in patients with acute coronary syndrome combining diabetes mellitus and concomitant chronic kidney disease[J]. Medical Journal of Chinese People's Liberation Army, 2014, 39(7) : 546-552. |
| 76 | Wang J. Protective effect of an acute oral intensive-dose of atorvastatin on myocardial injury following percutaneous coronary intervention by up-regulated expression of phosphatase and tension homolog deleted on chromosome ten (PTEN) in CD4+T lymphocytes. Experimental and Clinical Cardiology,2014. |
| 77 | Izawa A,Kashima Y,Miura T et al. Assessment of lipophilic vs. hydrophilic statin therapy in acute myocardial infarction – ALPS-AMI study.[J] .Circ J, 2015, 79: 161-8. | Combined with other lipid-lowering drugs |
| 78 | Nohara R,Daida H,Hata M et al. Effect of intensive lipid-lowering therapy with rosuvastatin on progression of carotid intima-media thickness in Japanese patients: Justification for Atherosclerosis Regression Treatment (JART) study.[J] .Circ J, 2012, 76: 221-9. |
| 79 | Bestehorn H P,Rensing U F,Roskamm H et al. The effect of simvastatin on progression of coronary artery disease. The Multicenter coronary Intervention Study (CIS).[J] .Eur Heart J, 1997, 18: 226-34. |
| 80 | Sdringola S, Gould KL, Zamarka LG et al. A 6 month randomized, double blind, placebo controlled, multi-center trial of high dose atorvastatin on myocardial perfusion abnormalities by positron emission tomography in coronary artery disease[J]. Am Heart J. 2008 Feb;155(2):245-53. | No report on the outcomes of interest/No specific outcome data |
| 81 | Guzmán E C,Hirata M H,Quintão E C et al. Association of the apolipoprotein B gene polymorphisms with cholesterol levels and response to fluvastatin in Brazilian individuals with high risk for coronary heart disease.[J] .Clin Chem Lab Med, 2000, 38: 731-6. |
| 82 | Wang Z X,Wang C Q,Li X Y et al. Changes of naturally occurring CD4(+)CD25(+) FOXP3(+) regulatory T cells in patients with acute coronary syndrome and the beneficial effects of atorvastatin treatment.[J] .Int Heart J, 2015, 56: 163-9. |
| 83 | Mohler E R,Hiatt W R,Creager M A. Cholesterol reduction with atorvastatin improves walking distance in patients with peripheral arterial disease.[J] .Circulation, 2003, 108: 1481-6. |
| 84 | Aydin MU, Aygul N, Altunkeser BB et al. Comparative effects of high-dose atorvastatin versus moderate-dose rosuvastatin on lipid parameters, oxidized-LDL and inflammatory markers in ST elevation myocardial infarction.[J]. Atherosclerosis. 2015 Apr;239(2):439-43. |
| 85 | Lee CW, Kang SJ, Ahn JM et al. Comparison of effects of atorvastatin (20 mg) versus rosuvastatin (10 mg) therapy on mild coronary atherosclerotic plaques (from the ARTMAP trial).[J]. Am J Cardiol. 2012 Jun 15;109(12):1700-4. |
| 86 | Meredith KG, Horne BD, Pearson RR et al. Comparison of effects of high (80 mg) versus low (20 mg) dose of simvastatin on C-reactive protein and lipoproteins in patients with angiographic evidence of coronary arterial narrowing.[J]. Am J Cardiol. 2007 Jan 15;99(2):149-53. |
| 87 | Hong Y J,Jeong M H,Hachinohe D et al. Comparison of effects of rosuvastatin and atorvastatin on plaque regression in Korean patients with untreated intermediate coronary stenosis.[J] .Circ J, 2011, 75: 398-406. |
| 88 | Li J,Sun Y M,Wang L F et al. Comparison of effects of simvastatin versus atorvastatin on oxidative stress in patients with coronary heart disease.[J] .Clin Cardiol, 2010, 33: 222-7. |
| 89 | Xie J, Wang YK, Shao Y, et al. Comparison of efficacy of two statins for peripheral artery atherosclerosis [J]. Chinese Journal of New Drugs 2015,24(7):808-812. |
| 90 | Hong S J,Park J Y,Kim K A et al. Comparison of low vs moderate dose of atorvastatin in clopidogrel resistance after coronary stenting in Korean patients with acute coronary syndrome.[J] .Circ J, 2009, 73: 1111-8. |
| 91 | Wang C, Pan X, Chen H, et al. The contribution of aggressive lipid lowering by lipitor to cardiovascular events after percutaneous coronary intervention [J]. Chinese Journal of Evidence-Based Medicine, 2007,7(4):244-250. |
| 92 | Shirakawa T,Fujisue K,Nakamura S et al. Dose-Dependent Inhibitory Effect of Rosuvastatin in Japanese Patients with Acute Myocardial Infarction on Serum Concentration of Matrix Metalloproteinases-INVITATION Trial.[J] .J Atheroscler Thromb, 2021, undefined: undefined. |
| 93 | Mulder Douwe J,van Haelst Paul L,Wobbes Martgriet H et al. The effect of aggressive versus conventional lipid-lowering therapy on markers of inflammatory and oxidative stress.[J] .Cardiovasc Drugs Ther, 2007, 21: 91-7. |
| 94 | Oleynikov V E, Lukianova MV, Dushina EV, et al. Effect of atorvastatin on the most important mechanisms of arrhythmogenesis in patients with ST-elevated myocardial infarction [J]. Journal of global pharma technology, 2019;24(7):83–90. |
| 95 | Soedamah-Muthu SS, Colhoun HM, Thomason MJ et al. The effect of atorvastatin on serum lipids, lipoproteins and NMR spectroscopy defined lipoprotein subclasses in type 2 diabetic patients with ischaemic heart disease.[J]. Atherosclerosis. 2003 Apr;167(2):243-55. |
| 96 | Chen Q Q,Shang X C,Yuan M et al. Effect of atorvastatin on serum omentin-1 in patients with coronary artery disease.[J] .Coron Artery Dis, 2017, 28: 44-51. |
| 97 | Liem A H,van Boven A J,Veeger N J G M et al. Effect of fluvastatin on ischaemia following acute myocardial infarction: a randomized trial.[J] .Eur Heart J, 2002, 23: 1931-7. |
| 98 | van der H P,Wagenaar L J,Buikema H et al. Effect of intensive versus moderate lipid lowering on endothelial function and vascular responsiveness to angiotensin II in stable coronary artery disease.[J] .Am J Cardiol, 2005, 96: 1361-4. |
| 99 | Shu X,Qiu YW,Gang G et al. The effect of lipid regulation with atorvastatin on the blood lipid levels and carotid artery plaques in patients with atherosclerotic cerebral infarction[J]. Chinese Journal of Contemporary Neurology and Neurosurgery, 2015, 15(11) : 904-908. |
| 100 | Bertrand M E,McFadden E P,Fruchart J C et al. Effect of pravastatin on angiographic restenosis after coronary balloon angioplasty. The PREDICT Trial Investigators. Prevention of Restenosis by Elisor after Transluminal Coronary Angioplasty.[J] .J Am Coll Cardiol, 1997, 30: 863-9. |
| 101 | Makuuchi H,Furuse A,Endo M et al. Effect of pravastatin on progression of coronary atherosclerosis in patients after coronary artery bypass surgery.[J] .Circ J, 2005, 69: 636-43. |
| 102 | Effect of simvastatin on coronary atheroma: the Multicentre Anti-Atheroma Study (MAAS)[J] .Lancet, 1994, 344: 633-8. |
| 103 | V V Yakusevich,A Yu Malygin, A V Kabanov. EFFECT OF SIMVASTATIN ON THE PROGNOSIS AND THE CHANGES OF THE CLINICAL STATUS IN PATIENTS WITH ACUTE ISCHEMIC STROKE. THE RESULTS OF THE 12 MONTH RANDOMIZED, OPEN COMPARATIVE STUDY[J]. Racionalʹnaâ Farmakoterapiâ v Kardiologii, 2013, 9(4) : 379-385. |
| 104 | Sasmazel A,Baysal A,Fedekar A et al. The effect of statin therapy on stimulation of endothelium-derived nitric oxide before and after coronary artery bypass surgery.[J] .Heart Surg Forum, 2010, 13: E243-6. |
| 105 | Cao G, Zhang X, Zheng K. Effects of atorvastatin and rosuvastatin on blood lipids, platelet aggregation rate and inflammatory factors in patients with cerebral infarction [J]. Tropical Journal of Pharmaceutical Research,2017, 16 (10): 2507-13. |
| 106 | Guo S X,Wang R X,Yang Z Y et al. Effects of atorvastatin on serum lipids, serum inflammation and plaque morphology in patients with stable atherosclerotic plaques.[J] .Exp Ther Med, 2012, 4: 1069-1074. |
| 107 | Sadeghi R,Asadpour P M,Asadollahi M et al. The effects of different doses of atorvastatin on serum lipid profile, glycemic control, and liver enzymes in patients with ischemic cerebrovascular accident.[J] .ARYA Atheroscler, 2014, 10: 298-304. |
| 108 | He W F,Cao M L,Li Z F. Effects of different doses of atorvastatin, rosuvastatin, and simvastatin on elderly patients with ST-elevation acute myocardial infarction (AMI) after percutaneous coronary intervention (PCI).[J] .Drug Dev Res, 2020, 81: 551-556. |
| 109 | Wang Y B,Fu X H,Gu X S et al. Effects of intensive pitavastatin therapy on glucose control in patients with non-ST elevation acute coronary syndrome.[J] .Am J Cardiovasc Dis, 2017, 7: 89-96. |
| 110 | Bae J H,Bassenge E ,Kim K Y et al. Effects of low-dose atorvastatin on vascular responses in patients undergoing percutaneous coronary intervention with stenting.[J] .J Cardiovasc Pharmacol Ther, 2004, 9: 185-92. |
| 111 | MacMahon S,Sharpe N,Gamble G et al. Effects of lowering average of below-average cholesterol levels on the progression of carotid atherosclerosis: results of the LIPID Atherosclerosis Substudy. LIPID Trial Research Group.[J] .Circulation, 1998, 97: 1784-90. |
| 112 | Li Y H,Wang L H,Li Q et al. Effects of rosuvastatin on pentraxin 3 level and platelet aggregation rate in elderly patients with acute myocardial infarction undergoing elective interventional therapy: a double-blind controlled study.[J] .Eur Rev Med Pharmacol Sci, 2017, 21: 3730-3735. |
| 113 | Kjekshus J,Pedersen T R,Olsson A G et al. The effects of simvastatin on the incidence of heart failure in patients with coronary heart disease.[J] .J Card Fail, 1997, 3: 249-54. |
| 114 | Hong M K,Park D W,Lee C W et al. Effects of statin treatments on coronary plaques assessed by volumetric virtual histology intravascular ultrasound analysis.[J] .JACC Cardiovasc Interv, 2009, 2: 679-88. |
| 115 | O. A. Rubanenko. Efficacy of atorvastatin therapy in prevention of postoperative atrial fibrillation in patients with ischemic heart disease[J]. Racionalʹnaâ Farmakoterapiâ v Kardiologii, 2015, 11(5) : 464-469. |
| 116 | Jin Y, Qiu C, Zheng Q, et al. Efficacy of different doses of atorvastatin treatment on serum levels of 8-hydroxy-guanin (8-OHdG) and cardiac function in patients with ischemic cardiomyopathy [J]. Pak J Med Sci, 2015,31(1):37-42. |
| 117 | Yakusevich VV, Malygin AY, Lychenko SV, et al. The efficacy of high-dose simvastatin in acute period of ischemic stroke [J]. Rational pharmacotherapy in cardiology, 2012, 8(1) : 4-16. |
| 118 | Zhang H L,Jiang M,Hou H et al. Efficacy of simvastatin on carotid atherosclerotic plaque and its effects on serum inflammatory factors and cardiocerebrovascular events in elderly patients.[J] .Exp Ther Med, 2021, 22: 819. |
| 119 | Bailey KM, Romaine SPR, Jackson BM, et al. Hepatic metabolism and transporter gene variants enhance response to rosuvastatin in patients with acute myocardial infarction: the GEOSTAT-1 Study [J] .Circ Cardiovasc Genet, 2010, 3: 276-85. |
| 120 | Yang Q, Guo SL, Chen Y, et al.Intervention of pr avastatin for carotid atherosclerotic plaques and C-r eactive protein in young and middle-aged patients with cer ebr al infar ction [J]. 中国组织工程研究与临床复,2007(08):1525-1528. |
| 121 | Weintraub WS, Boccuzzi SJ,Klein JL, et al. Lack of effect of lovastatin on restenosis after coronary angioplasty. Lovastatin Restenosis Trial Study Group.[J] .N Engl J Med, 1994, 331: 1331-7. |
| 122 | Zou Y C,Hu D Y,Yang X C et al. Lipid-lowering efficacy and safety of varying doses of Simvastatin in patients with early stage acute coronary syndromes: one-year follow-up study.[J] .Chin Med J (Engl), 2003, 116: 853-6. |
| 123 | Xu K,Han Y L,Jing Q M et al. Lipid-modifying therapy in diabetic patients with high plasma non-high-density lipoprotein cholesterol after percutaneous coronary intervention.[J] .Exp Clin Cardiol, 2007, 12: 48-50. |
| 124 | Hong S J,Choi S C,Kim J S et al. Low-dose versus moderate-dose atorvastatin after acute myocardial infarction: 8-month effects on coronary flow reserve and angiogenic cell mobilisation.[J] .Heart, 2010, 96: 756-64. |
| 125 | Altaf A,Qu P,Zhao Y et al. NLRP3 inflammasome in peripheral blood monocytes of acute coronary syndrome patients and its relationship with statins.[J] .Coron Artery Dis, 2015, 26: 409-21. |
| 126 | Byington R P,Furberg C D,Crouse J R et al. Pravastatin, Lipids, and Atherosclerosis in the Carotid Arteries (PLAC-II).[J] .Am J Cardiol, 1995, 76: 54C-59C. |
| 127 | Iwasaki K,Kusachi S,Hina K et al. [Preventive effect of pravastatin on restenosis following coronary angioplasty: prospective randomized trial].[J] .J Cardiol, 1995, 25: 15-21. |
| 128 | Nakagawa T,Kobayashi T,Awata N et al. Randomized, controlled trial of secondary prevention of coronary sclerosis in normocholesterolemic patients using pravastatin: final 5-year angiographic follow-up of the Prevention of Coronary Sclerosis (PCS) study.[J] .Int J Cardiol, 2004, 97: 107-14. |
| 129 | Hodis H N,Mack W J,LaBree L et al. Reduction in carotid arterial wall thickness using lovastatin and dietary therapy: a randomized controlled clinical trial.[J] .Ann Intern Med, 1996, 124: 548-56. |
| 130 | van Boven AJ, Jukema JW, Zwinderman AH et al. Reduction of transient myocardial ischemia with pravastatin in addition to the conventional treatment in patients with angina pectoris. REGRESS Study Group.[J]. Circulation. 1996 Oct 1;94(7):1503-5. |
| 131 | Jiang F Y,Yang J,Zhang L C et al. Rosuvastatin reduces ischemia-reperfusion injury in patients with acute coronary syndrome treated with percutaneous coronary intervention.[J] .Clin Cardiol, 2014, 37: 530-5. |
| 132 | Mostafa S A, Elrabat KH, Mahrous M, Kamal M et al. Short Term Comparison Between Safety and Efficacy of Rosuvastatin 40 mg and Atorvastatin 80 mg in Patients with Acute Coronary Syndrome[J]. Rational Pharmacotherapy in Cardiology, 2018, 14(5) : 636-645. |
| 133 | Nissen SE, Tuzcu EM, Schoenhagen P et al. Statin therapy, LDL cholesterol, C-reactive protein, and coronary artery disease.[J]. N Engl J Med. 2005 Jan 6;352(1):29-38. |
| 134 | Guo J, Zhang WZ, Zhao Q et al. Study on the effect of different doses of rosuvastatin on ventricular remodeling in patients with acute coronary syndrome after emergency percutaneous coronary intervention.[J]. Eur Rev Med Pharmacol Sci. 2017 Oct;21(19):4457-4463. |
| 135 | Boekholdt S M,Agema W R P,Peters R J G et al. Variants of toll-like receptor 4 modify the efficacy of statin therapy and the risk of cardiovascular events.[J] .Circulation, 2003, 107: 2416-21. |
| 136 | Ma QL, Mo L, Yang TL et al. [Effect of different doses of atorvastatin on adhesion molecules of the patients undergoing percutaneous coronary intervention].[J]. Zhong Nan Da Xue Xue Bao Yi Xue Ban. 2006 Dec;31(6):914-6. |
| 137 | Oleynikov V E, Lukianova M V, Dushina E V et al. Effect of atorvastatin on the most important mechanisms of arrhythmogenesis in patients with ST-elevated myocardial infarction[J]. Russian Journal of Cardiology, 2019: 83-90. |
| 138 | Tian CF. Protective effects of statins on renal function in patients with carotid atherosclerotic plaques. Acta medica mediterranea, 2018. |
| 139 | Ballantyne C M,Bertolami M,Hernandez G et al. Achieving LDL cholesterol, non-HDL cholesterol, and apolipoprotein B target levels in high-risk patients: Measuring Effective Reductions in Cholesterol Using Rosuvastatin therapY (MERCURY) II.[J] .Am Heart J, 2006, 151: 975.e1-9. | Not ASCVD populations |
| 140 | Luijendijk P,Bouma B J,Vriend J W J et al. Beneficial effect of high dose statins on the vascular wall in patients with repaired aortic coarctation?[J] .Int J Cardiol, 2014, 176: 40-7. |
| 141 | Clearfield M B,Amerena J,Bassand J P et al. Comparison of the efficacy and safety of rosuvastatin 10 mg and atorvastatin 20 mg in high-risk patients with hypercholesterolemia--Prospective study to evaluate the Use of Low doses of the Statins Atorvastatin and Rosuvastatin (PULSAR).[J] .Trials, 2006, 7: 35. |
| 142 | van Dam M J,Penn H J,den Hartog F R et al. A comparison of the efficacy and tolerability of titrate-to-goal regimens of simvastatin and fluvastatin: a randomized, double-blind study in adult patients at moderate to high risk for cardiovascular disease.[J] .Clin Ther, 2001, 23: 467-78. |
| 143 | Schouten O,Boersma E,Hoeks S E et al. Fluvastatin and perioperative events in patients undergoing vascular surgery.[J] .N Engl J Med, 2009, 361: 980-9. |
| 144 | Kadoglou N P E,Vrabas I S,Kapelouzou A et al. Impact of atorvastatin on serum vaspin levels in hypercholesterolemic patients with moderate cardiovascular risk.[J] .Regul Pept, 2011, 170: 57-61. |
| 145 | Moroi M,Nagayama D,Hara F et al. Outcome of pitavastatin versus atorvastatin therapy in patients with hypercholesterolemia at high risk for atherosclerotic cardiovascular disease.[J] .Int J Cardiol, 2020, 305: 139-146. |
| 146 | Shepherd J, Blauw GJ, Murphy MB et al. Pravastatin in elderly individuals at risk of vascular disease (PROSPER): a randomised controlled trial.[J]. Lancet. 2002 Nov 23;360(9346):1623-30. |
| 147 | Heart Protection Study Collaborative Group,Randomized trial of the effects of cholesterol-lowering with simvastatin on peripheral vascular and other major vascular outcomes in 20,536 people with peripheral arterial disease and other high-risk conditions.[J] .J Vasc Surg, 2007, 45: 645-654; discussion 653-4. |
| 148 | Atique M. Comparative Effectiveness of Atorvastatin (Low Vs High Dose) in Lowering Low-Density Lipoprotein Cholesterol in Intermediate Risk Cardiovascular Patients [J]. Pakistan Journal of Medical and Health Sciences, 2020. |
| 149 | Lu Y, Zhao X, Qin Y. Clinial research on efficacy of statins in preventing contrast-induced nephropathy[J]. Pharmaceutical care and research, 2011,11(02):111-4. | Sample size <100 |
| 150 | Hou J, Xing L, Jia H et al. Comparison of Intensive Versus Moderate Lipid-Lowering Therapy on Fibrous Cap and Atheroma Volume of Coronary Lipid-Rich Plaque Using Serial Optical Coherence Tomography and Intravascular Ultrasound Imaging.[J]. Am J Cardiol. 2016 Mar 1;117(5):800-6. |
| 151 | Onaka H,Hirota Y,Kita Y et al. The effect of pravastatin on prevention of restenosis after successful percutaneous transluminal coronary angioplasty.[J] .Jpn Circ J, 1994, 58: 100-6. |
| 152 | Khan S , Abrar A , Rafique A , et al. Efficacy and Safety of Rosuvastatin Compared to Simvaststin in Coronary Artery Disease[J]. Gomal Journal of Medical Sciences, 2010, 8. |
| 153 | Suh Y,Kim B K,Shin D H et al. Impact of statin treatment on strut coverage after drug-eluting stent implantation.[J] .Yonsei Med J, 2015, 56: 45-52. |
| 154 | Miedema M D,Conover C A,MacDonald H et al. Pregnancy-associated plasma protein-A elevation in patients with acute coronary syndrome and subsequent atorvastatin therapy.[J] .Am J Cardiol, 2008, 101: 35-9. |
| 155 | Sahni R,Maniet A R,Voci G et al. Prevention of restenosis by lovastatin after successful coronary angioplasty.[J] .Am Heart J, 1991, 121: 1600-8. |
| 156 | Wenaweser P, Eshtehardi P, Abrecht L et al. A randomised determination of the Effect of Fluvastatin and Atorvastatin on top of dual antiplatelet treatment on platelet aggregation after implantation of coronary drug-eluting stents. The EFA-Tria.[J]l. Thromb Haemost. 2010 Sep;104(3):554-62. |
| 157 | Papathanasiou AI, Lourida ES, Tsironis LD et al. Short- and long-term elevation of autoantibody titers against oxidized LDL in patients with acute coronary syndromes. Role of the lipoprotein-associated phospholipase A2 and the effect of atorvastatin treatment.[J]. Atherosclerosis. 2008 Jan;196(1):289-297. |
| 158 | Mal'Gina M P , Ignatyeva O I , Moroshkina N V , et al. Effectiveness, safety, and endothelial function effects of atorvastatin lipid-lowering therapy in coronary heart disease patients undergoing percutaneous coronary intervention[J]. Cardiovascular Therapy & Prevention, 2007, 6(3):50-55. |
| 159 | Sergienko IV, Samoĭlenko EIu, Masenko VP, et al. Effect of therapy with rosuvastatin on lipid spectrum, factors of inflammation and endothelial function in patients with ischemic heart disease [J]. Kardiologiia. 2006;46(5):4-8. |
| 160 | Atorvastatin effectiveness in dyslipidemia correction among coronary heart disease and arterial hypertension patients with high risk of cardiovascular complications. https://www.cochranelibrary.com/central/doi/10.1002/central/CN-00871118/full | Full-texts not available |
| 161 | Comparative efficacy and safety profile of 5 MG rosuvastatin versus 10 MG rosuvastatin in patients with ischemic heart disease. https://www.embase.com/search/results?subaction=viewrecord&id=L369253811&from=export |
| 162 | Comparative evaluation of the efficacy and safety of rosuvastatin vs atorvastatin in patients of dyslipidemia with coronary heart disease in Indian Scenario. https://www.cochranelibrary.com/central/doi/10.1002/central/CN-01132974/full |
| 163 | Iakovenko EI, Evdakimova AA, Toguzova Z A, et al. Dose-dependent effect of atorvastatin on erectile function and androgen status in men with high cardiovascular risk [J] .Kardiologiia, 2014, 54: 37-42. |
| 164 | Shal'nev V I. [The effects of early application of simvastatin on C-reactive protein level, blood lipids, and the clinical course of acute coronary syndrome].[J] .Klin Med (Mosk), 2007, 85: 46-50. |
| 165 | Shukla A, Sharma MK, Jain A, et al. Prevention of atherosclerosis progression using atorvastatin in normolipidemic coronary artery disease patients--a controlled randomized trial [J] .Indian Heart J, 2005, 57: 675-80. |
| 166 | Atorvastatin is more effective than pravastatin in preventing recurrent cardiac events[J]. Evidence-Based Healthcare and Public Health, 2004, 8(5) : 296-7. | Abstract |
| 167 | Margarite J. Vale. Intensive lipid lowering with atorvastatin reduces progression of coronary atherosclerosis, as measured by intravascular ultrasound[J]. Evidence-Based Healthcare and Public Health, 2004, 8(5) : 298-301. |
| 168 | Scheen AJ. Clinical study of the month. The LIPID study: "long-term intervention with pravastatin in ischaemic disease" [J]. Rev Med Liege. 1999, 54(1):2-3. |
| 169 | Abletshauser C, Riegger G, Schwandt P, et al. The effect of fluvastatin on cardiac events in hyperlipidemic patients with symptomatic coronary heart disease[J]. Atherosclerosis, 1999, 144(S1) : 35-36. |
| 170 | Hunt D, Keech A, Thomson A, et al. Impact of cholesterol lowering treatment with pravastatin in women and elderly patients with coronary heart disease (CHD) and average cholesterol levels[J]. Australian and new zealand journal of medicine, 1999, 29(1):148. |
| 171 | Simvastatin reduces stroke and major vascular events in people at high-risk[J]. Evidence-Based Healthcare and Public Health, 2004, 8(5) : 294-295. |
| 172 | Furberg CD, Byington RP, Crouse JR et al. Pravastatin, lipids, and major coronary events.[J]. Am J Cardiol. 1994 Jun 1;73(15):1133-4. | Duplicate publication |
| 173 | Tseng M Y , Czosnyka M , Richards H , et al. Biological effects of acute pravastatin therapy on cerebral vasospasm, delayed ischemic deficits, and outcome in patients following aneurysmal subarachnoid hemorrhage: A randomised controlled trial[C]// Meeting of the American-association-of-neurological-surgeons. 2006. | Conference Papers |
| 174 | DUAAL: atorvastatin provided an unexpectedly potent heart benefit in patients with chronic stable angina.[J] .Cardiovasc J Afr, 2008, 19: 174. |
| 175 | Chen HS. The Effect of InTensive Statin in Ischemic Stroke With inTracranial Atherosclerotic Plaques (INSIST-HRMRI). | Uncompleted trial |

**Table S4. Risk of bias in the included trials as assessed by the Cochrane risk of bias assessment tool**

| **Studies** | **Random Sequence generation** | **Allocation concealment** | **Blinding of participants and personnel** | **Blinding of outcome assessment** | **Incomplete outcome data** | **Selective outcome reporting** | **Other bias** |
| --- | --- | --- | --- | --- | --- | --- | --- |
| MARS, 1993 | Low risk | Low risk | Low risk (double-blind) | Low risk | Low risk | Unclear | Low risk |
| Oxford Cholesterol, 1994 | Low risk | Unclear | Low risk (single-blind) | Low risk | Low risk | Unclear | Low risk |
| 4S, 1994 | Low risk | Low risk | Low risk (double-blind) | Low risk | Low risk | Low risk | Low risk |
| PLAC I, 1995 | Unclear | Unclear | Low risk (double-blind) | Low risk | Low risk | Unclear | Low risk |
| CARE, 1996 | Low risk | Low risk | Low risk (double-blind) | Low risk | Low risk | Unclear | Low risk |
| LIPID, 1998 | Unclear | Unclear | Low risk (double-blind) | Low risk | Low risk | Unclear | Low risk |
| TARGET TANGIBLE, 1999 | Unclear | Low risk | High risk  (open-label) | Low risk | Low risk | Unclear | Low risk |
| FLARE, 1999 | Low risk | Low risk | Low risk (double-blind) | Low risk | Low risk | Unclear | High risk |
| MIRACL, 2001 | Low risk | Low risk | Low risk (double-blind) | Low risk | Low risk | Low risk | Low risk |
| Karalis et al, 2002 | Unclear | Unclear | High risk  (open-label) | Low risk | Low risk | Unclear | Low risk |
| LIPS, 2002 | Low risk | Low risk | Low risk (double-blind) | Low risk | Low risk | Low risk | Low risk |
| HPS, 2002 | Low risk | Low risk | Low risk (double-blind) | Low risk | Low risk | Low risk | Low risk |
| 3T, 2003 | Low risk | Low risk | Low risk (double-blind) | Low risk | Low risk | Low risk | Low risk |
| REVERSAL, 2004 | Low risk | Low risk | Low risk (double-blind) | Low risk | High risk | Low risk | Low risk |
| Schwartz et al, 2004 | Unclear | Unclear | Low risk (double-blind) | Unclear | Low risk | Unclear | Low risk |
| PROVE IT–TIMI 22, 2004 | Low risk | Low risk | Low risk (double-blind) | Low risk | Low risk | Low risk | Low risk |
| JUST, 2004 | Unclear | Unclear | High risk  (open-label) | Low risk | Low risk | Unclear | Low risk |
| IDEAL, 2005 | Low risk | Unclear | High risk  (open-label) | Low risk | Low risk | Low risk | Low risk |
| TNT, 2005 | Low risk | Low risk | Low risk (double-blind) | Low risk | Low risk | Low risk | Low risk |
| ATHEROMA, 2005 | Low risk | Low risk | Unclear | Low risk | Low risk | Unclear | Low risk |
| SPARCL, 2006 | Low risk | Low risk | Low risk (double-blind) | Low risk | Low risk | Low risk | Low risk |
| SOLAR, 2007 | Unclear | Unclear | High risk  (open-label) | Low risk | Low risk | Unclear | Low risk |
| ARIANE, 2007 | Unclear | Unclear | High risk  (open-label) | Unclear | Unclear | Unclear | Unclear |
| Kyeong et al, 2007 | Unclear | Unclear | High risk  (open-label) | Unclear | Low risk | Unclear | Unclear |
| Yu et al, 2007 | Low risk | Low risk | Low risk (double-blind) | Low risk | Low risk | Unclear | Low risk |
| SAGE, 2007 | Low risk | Low risk | Low risk (double-blind) | Low risk | Low risk | Low risk | Low risk |
| CAP, 2008 | Low risk | Low risk | Low risk (double-blind) | Low risk | Low risk | Unclear | Low risk |
| JAPAN-ACS, 2009 | Low risk | Low risk | High risk  (open-label) | Low risk | Low risk | Low risk | Low risk |
| Zhao et al, 2009 | Low risk | Unclear | Low risk (double-blind) | Unclear | Unclear | Unclear | Low risk |
| SPACE ROCKET, 2009 | Low risk | Low risk | High risk  (open-label) | Low risk | Low risk | Unclear | Low risk |
| Mok et al, 2009 | Low risk | Low risk | Low risk (double-blind) | Low risk | Low risk | Unclear | Low risk |
| CENTAURUS, 2010 | Low risk | Low risk | Low risk (double-blind) | Low risk | Low risk | Low risk | Low risk |
| FACS, 2010 | Low risk | Low risk | Low risk (double-blind) | Low risk | Low risk | Low risk | Low risk |
| SEARCH, 2010 | Low risk | Low risk | Low risk (double-blind) | Low risk | Low risk | Low risk | Low risk |
| LUNAR, 2012 | Low risk | Low risk | High risk  (open-label) | Low risk | Unclear | Low risk | Low risk |
| TRUTH, 2012 | Low risk | Unclear | High risk  (open-label) | Low risk | Low risk | Low risk | Low risk |
| CURE-ACS, 2013 | Low risk | Low risk | High risk  (open-label) | Unclear | Unclear | Unclear | Low risk |
| PACT, 2014 | Unclear | Low risk | Low risk (double-blind) | Low risk | Low risk | Low risk | Low risk |
| Zhou et al, 2014 | Low risk | Unclear | Unclear | Low risk | Unclear | Unclear | Low risk |
| Khurana et al, 2015 | Unclear | Unclear | High risk  (open-label) | Unclear | Unclear | Unclear | High risk |
| J-STARS, 2015 | Low risk | Low risk | High risk  (open-label) | Low risk | High risk | Low risk | Unclear |
| Liu et al, 2016 | Low risk | Unclear | High risk  (open-label) | Unclear | Low risk | Unclear | Low risk |
| Priti et al, 2017 | Low risk | Unclear | Low risk (double-blind) | Unclear | Unclear | Unclear | Low risk |
| ACTIVE, 2018 | Low risk | Unclear | Low risk (double-blind) | Unclear | High risk | Low risk | Low risk |
| Liu et al, 2018 | Unclear | Unclear | High risk  (open-label) | Unclear | Low risk | Unclear | Unclear |
| Wang et al, 2020 | Unclear | Unclear | Unclear | Unclear | Low risk | Unclear | Unclear |
| Kim et al, 2021 | Low risk | Unclear | High risk  (open-label) | Unclear | Low risk | Low risk | Low risk |

**Table S5. GRADE profile for pairwise meta-analyses**

| **Certainty assessment** | | | | | | | **Summary of findings** | | | | |
| --- | --- | --- | --- | --- | --- | --- | --- | --- | --- | --- | --- |
| **No of participants,**  **No of studies; Mean follow-up** | **Risk of bias** | **Inconsistency** | **Indirectness** | **Imprecision** | **Publication bias** | **Overall certainty of evidence** | **Study event rates (%)** | | **Relative effect (95% CI)** | **Anticipated absolute effects (event rate per 10,000 people throughout mean follow-up)** | |
|  |  |  |  |  |  |  | **With Control** | **With Statins** |  | **Risk with Control** | **Risk difference with Statins** |
| **Muscle condition** | | | | | | | | | | | |
| 54602, 13; 4.5 years | not serious | serious ^a^ | not serious | not serious | none | ⨁⨁⨁◯ Moderate | 229/27197 (0.8%) | 231/27405 (0.8%) | OR 1.00 (0.83 to 1.20) | 84 | 0 (-14 to 17) |
| **Transaminase elevations** | | | | | | | | | | | |
| 54674, 14; 4.5 years | not serious | serious ^b^ | not serious | not serious | none | ⨁⨁⨁◯ Moderate | 426/27236 (1.6%) | 562/27438 (2.0%) | OR 1.62  (1.20 to 2.18) | 156 | 95 (31 to 178) |
| **Renal insufficiency** | | | | | | | | | | | |
| 1082, 3; 2.8 years | not serious | serious ^c^ | not serious | not serious | none | ⨁⨁⨁◯ Moderate | 11/441 (2.5%) | 12/641 (1.9%) | OR 0.74 (0.18 to 3.08) | 249 | -64 (-204 to 481) |
| **Gastrointestinal discomfort** | | | | | | | | | | | |
| 8274, 6; 4.2 years | not serious | serious ^d^ | not serious | not serious | none | ⨁⨁⨁◯ Moderate | 223/4043 (5.5%) | 276/4231 (6.5%) | OR 1.23 (1.02 to 1.48) | 552 | 118 (10 to 244) |
| **Cancer** | | | | | | | | | | | |
| 32989,7; 5.0 years | not serious | not serious | not serious | not serious | none | ⨁⨁⨁⨁ High | 1161/16492  (7.0%) | 1175/16497  (7.1%) | OR 1.01  (0.93 to 1.10) | 704 | 7 (-46 to 65) |
| **Myocardial Infarction** | | | | | | | | | | | |
| 44841, 9; 4.7 years | not serious | not serious | not serious | not serious | none | ⨁⨁⨁⨁ High | 1654/22407 (7.4%) | 1125/22434 (5.0%) | OR 0.66 (0.61 to 0.71) | 738 | -238 (-275 to -203) |
| **Stroke** | | | | | | | | | | | |
| 51939, 11; 4.6 years | not serious | not serious | not serious | not serious | none | ⨁⨁⨁⨁ High | 1365/25962 (5.3%) | 1078/25977 (4.1%) | OR 0.78 (0.72 to 0.84) | 526 | -111 (-142 to -80) |
| **Death from CVD** | | | | | | | | | | | |
| 44860, 7; 5.2 years | not serious | not serious | not serious | not serious | none | ⨁⨁⨁⨁ High | 1818/22422 (8.1%) | 1435/22438 (6.4%) | OR 0.77 (0.72 to 0.83) | 811 | -175 (-213 to -128) |
| **All-cause death** | | | | | | | | | | | |
| 53023, 13; 4.5 years | not serious | not serious | not serious | not serious | none | ⨁⨁⨁⨁ High | 2974/26510 (11.2%) | 2534/26513 (9.6%) | OR 0.83 (0.79 to 0.88) | 1122 | -173 (-214 to -121) |

RCT, randomized controlled trial; CI, confidence interval; CVD, cardiovascular diseases; OR, odds ratio; GRADE, Grading of Recommendations Assessment, Development, and Evaluation.

^a^ Four studies reported myalgia, 4 reported myopathy, 4 reported rhabdomyolysis, and 1 reported the above outcomes.

^b^ Ten studies reported the transaminase elevated >3 times ULN, 2 reported the transaminase elevated >2 times ULN, 2 reported non-specific transaminase elevation.

^c^ One studies reported the elevation of creatinine, 2 reported non-specific renal insufficiency.

^d^ One study reported diarrhea, 1 reported nausea, 3 reported non-specific gastrointestinal discomfort

**Table S6. GRADE profile for significant results from network meta-analyses**

| **Outcome** | **Treatment** | **Comparator** | **Direct Evidence** | | **Indirect Evidence** | | **Combined Evidence** | |
| --- | --- | --- | --- | --- | --- | --- | --- | --- |
|  |  |  | **OR (95% CI)** | **Quality** | **OR (95% CI)** | **Quality** | **OR (95% CI)** | **Quality** |
| **Transaminase elevations** | Atorvastatin | Control | 5.56 (2 to 16.67) | Moderate^a^ | 3.33 (1.39 to 8.17) | Moderate^a^ | 4.05 (2.18 to 7.60) | Moderate^a^ |
| **Transaminase elevations** | Atorvastatin | Pravastatin | 4.06 (1.59 to 11.03) | High | 3.00 (1.09 to 8.17) | Moderate^a^ | 3.49 (1.77, 6.92) | High* |
| **Transaminase elevations** | Atorvastatin | Simvastatin | 2.08 (0.73 to 5.56) | Moderate^a^ | 3.22 (1.10 to 9.97) | Moderate^a^ | 2.77 (1.31, 5.09) | Moderate^a^ |
| **Cancer** | Atorvastatin | Control | / | / | / | / | 6.61e-06 (6.71e-16, 0.47) | Low^b^ |
| **Cancer** | Atorvastatin | Fluvastatin | / | / | / | / | 7.22e-06 (7.18e-16, 0.54) | Low^b^ |
| **Cancer** | Atorvastatin | Lovastatin | / | / | / | / | 5.17e-06 (4.81e-16, 0.44) | Low^b^ |
| **Cancer** | Atorvastatin | Pravastatin | 6.46e-06 (6.67e-16, 0.46) | Low^b^ | / | / | 6.46e-06 (6.67e-16, 0.46) | Low^b^ |
| **Cancer** | Atorvastatin | Pitavastatin | / | / | 6.73e-12 (5.82e-25, 0.01) | Low^b^ | 6.73e-12 (5.82e-25, 0.01) | Low^b^ |
| **Cancer** | Atorvastatin | Simvastatin | / | / | 6.68e-06 (6.80e-16, 0.48) | Low^b^ | 6.68e-06 (6.80e-16, 0.48) | Low^b^ |
| **Cancer** | Pitavastatin | Control | / | / | 7.65e-06 (9.21e-17, 0.58) | Low^b^ | 7.65e-06 (9.21e-17, 0.58) | Low^c^ |
| **Cancer** | Pitavastatin | Fluvastatin | / | / | 7.06e-06 (8.35e-17, 0.55) | Low^b^ | 7.06e-06 (8.35e-17, 0.55) | Low^c^ |
| **Cancer** | Pitavastatin | Lovastatin | / | / | 1.01e-05 (8.99e-17, 0.83) | Low^b^ | 1.01e-05 (8.99e-17, 0.83) | Low^c^ |
| **Cancer** | Pitavastatin | Pravastatin | 7.76e-06 (9.50e-17, 0.58) | Low^c^ | / | / | 7.76e-06 (9.50e-17, 0.58) | Low^c^ |
| **Cancer** | Pitavastatin | Simvastatin | / | / | 7.58e-06 (9.05e-17, 0.58) | Low^b^ | 7.58e-06 (9.05e-17, 0.58) | Low^c^ |

OR: odds ratio, CI: confidence interval

* Combined evidence quality is determined by the higher level when the quality of direct and indirect evidence is not equal.

^a^ Inconsistency due to different specific conditions reported in individual studies for the outcome.

^b^ Only one study (REVERSAL, 2004) used atorvastatin and only 2 and 0 cases of cancers were reported in pravastatin and atorvastatin group, respectively.

^c^ Only one small scale study (TRUTH, 2012) used pitavastatin and only 2 and 0 cases of cancers were reported in pitavastatin and pravastatin group, respectively.

**Figure S1. Forest plots of pair-wise meta-analyses (including results from fixed and random effects models)**

A

Muscle condition


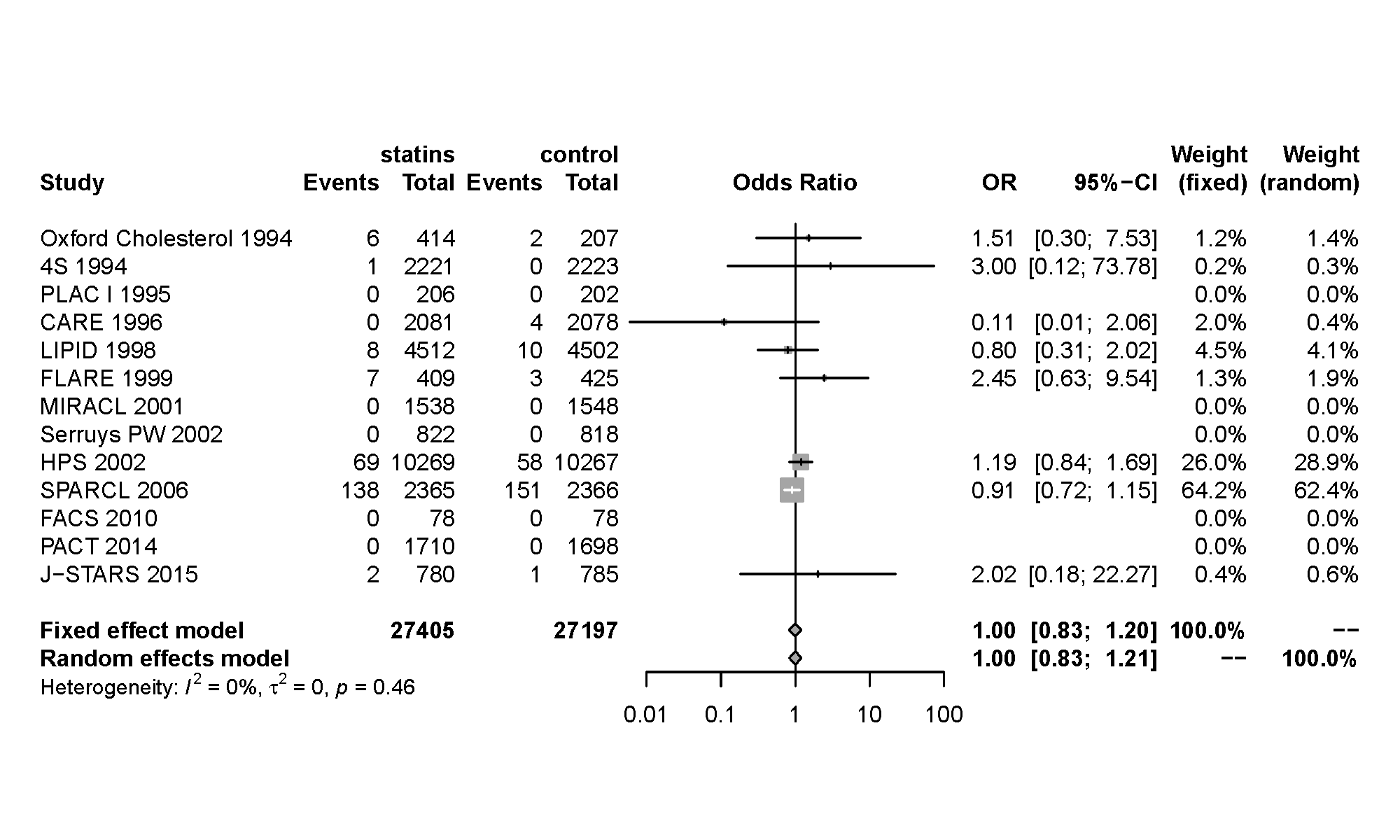


B

Transaminase elevations


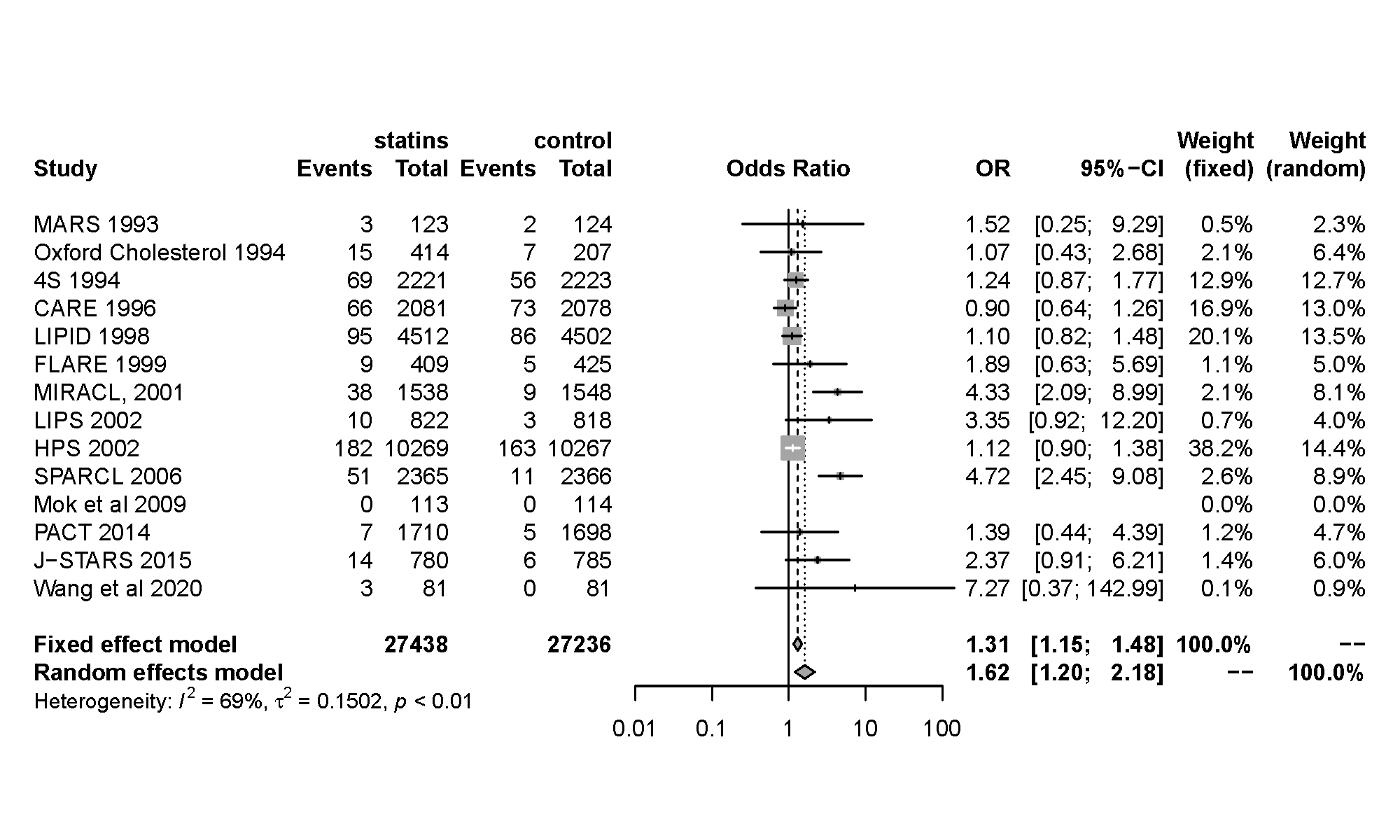


C

Renal Insufficiency


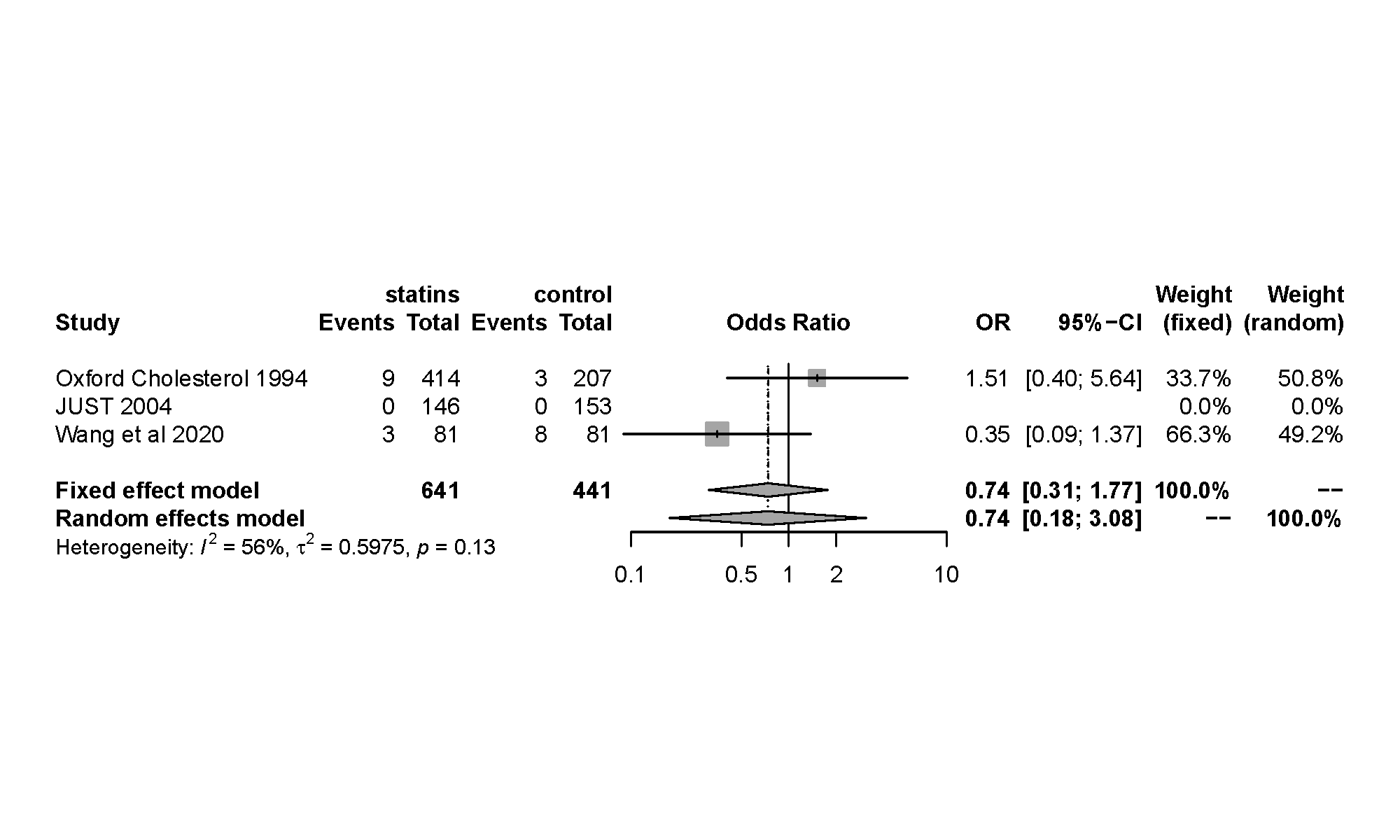


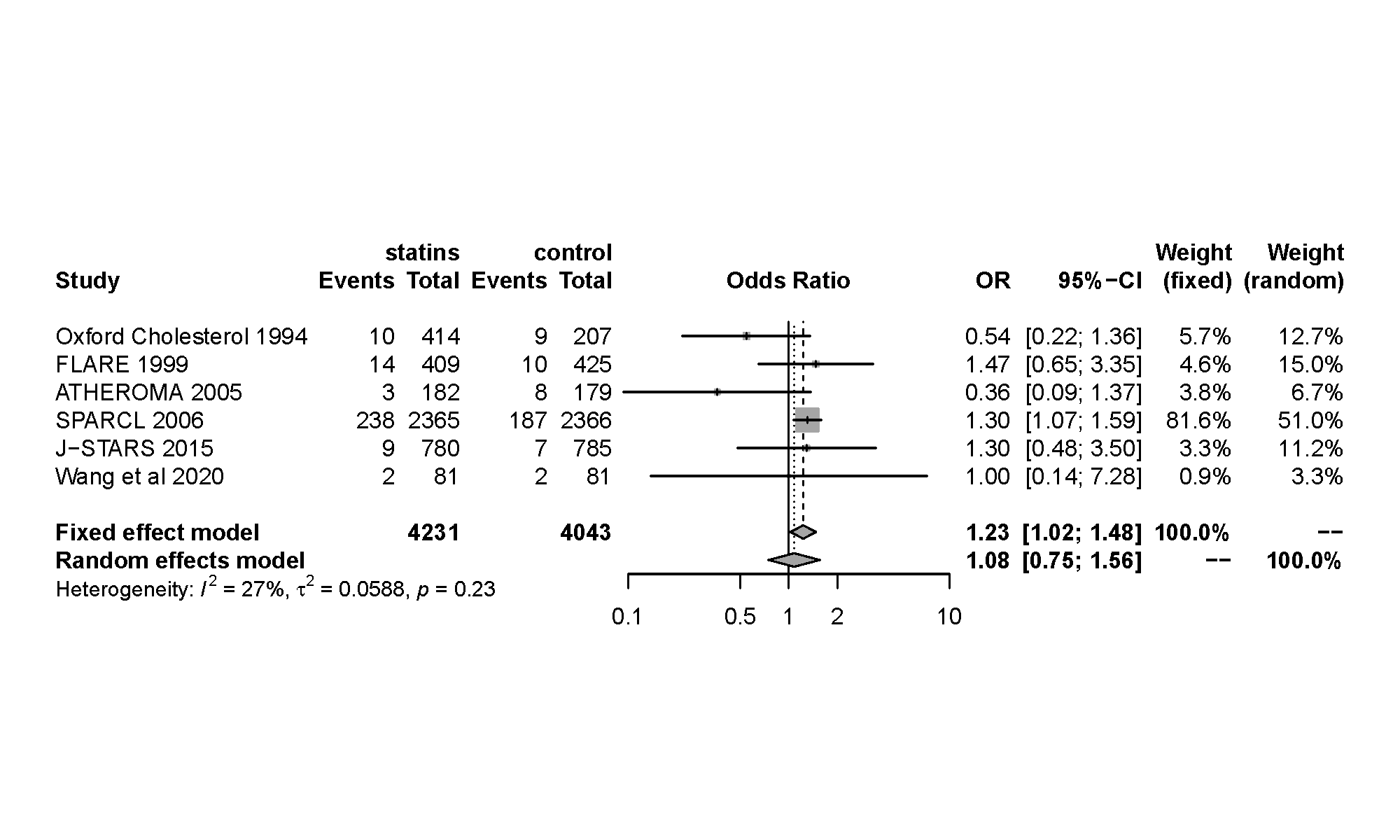


D

Gastrointestinal discomfort

E

Cancer


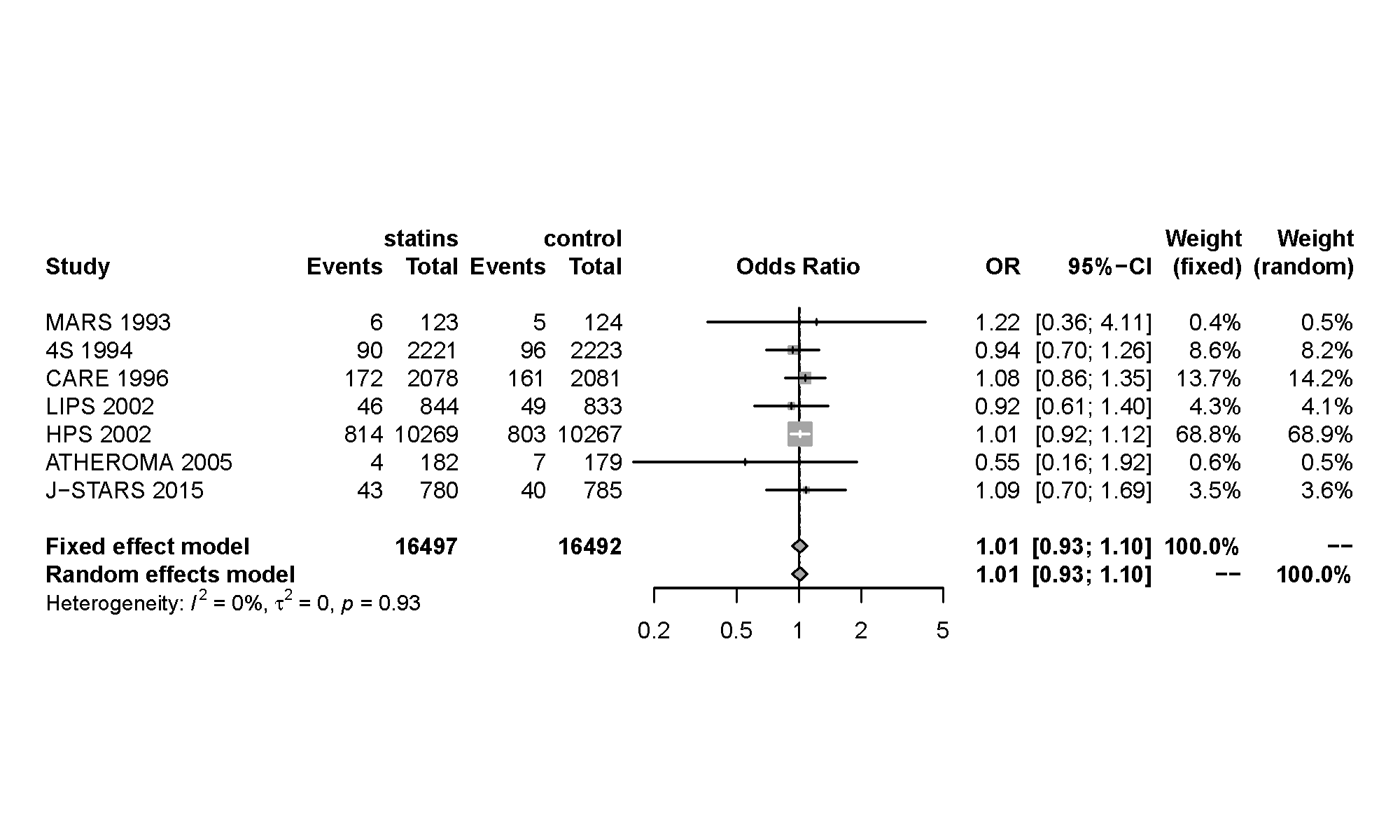


F

Myocardial Infarction


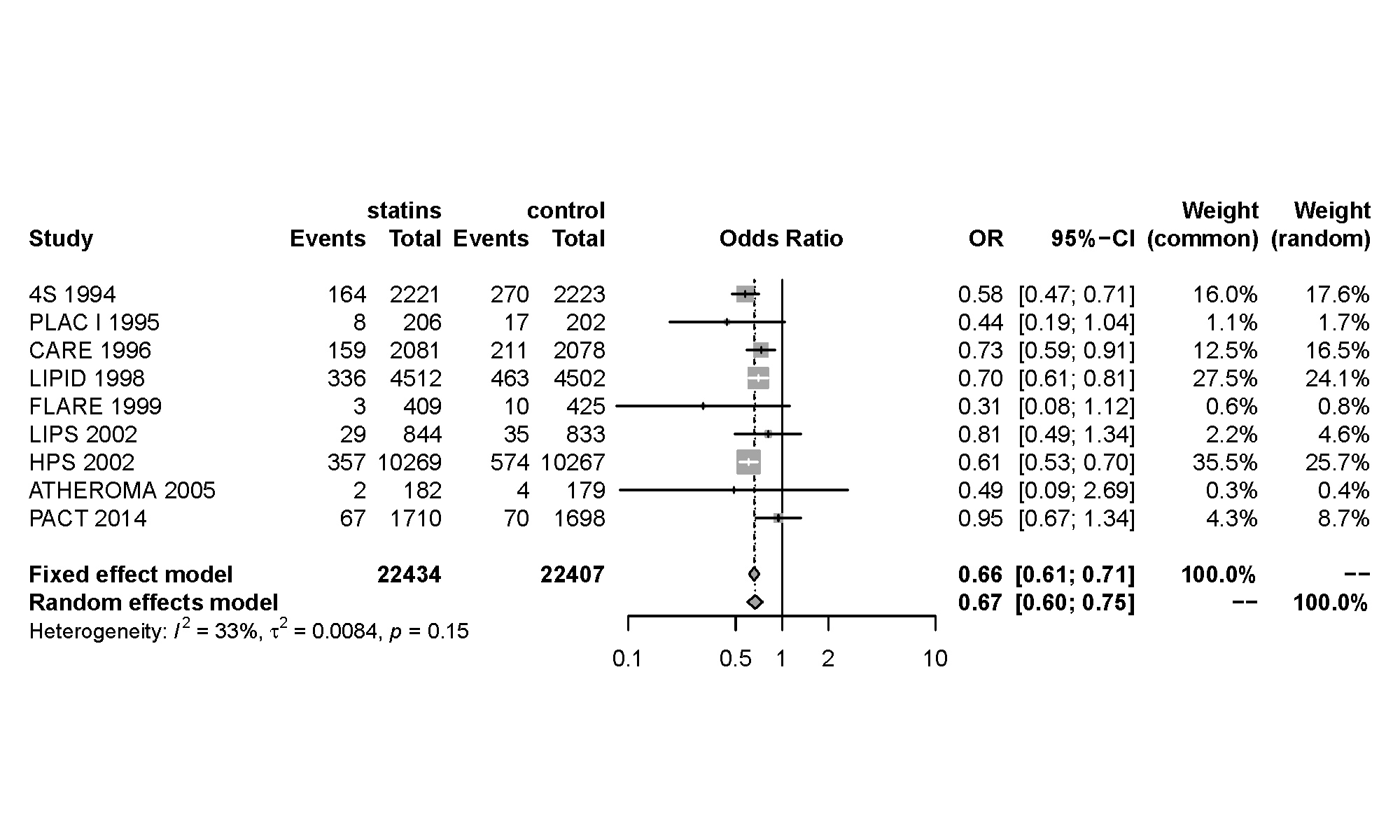


G

Stroke


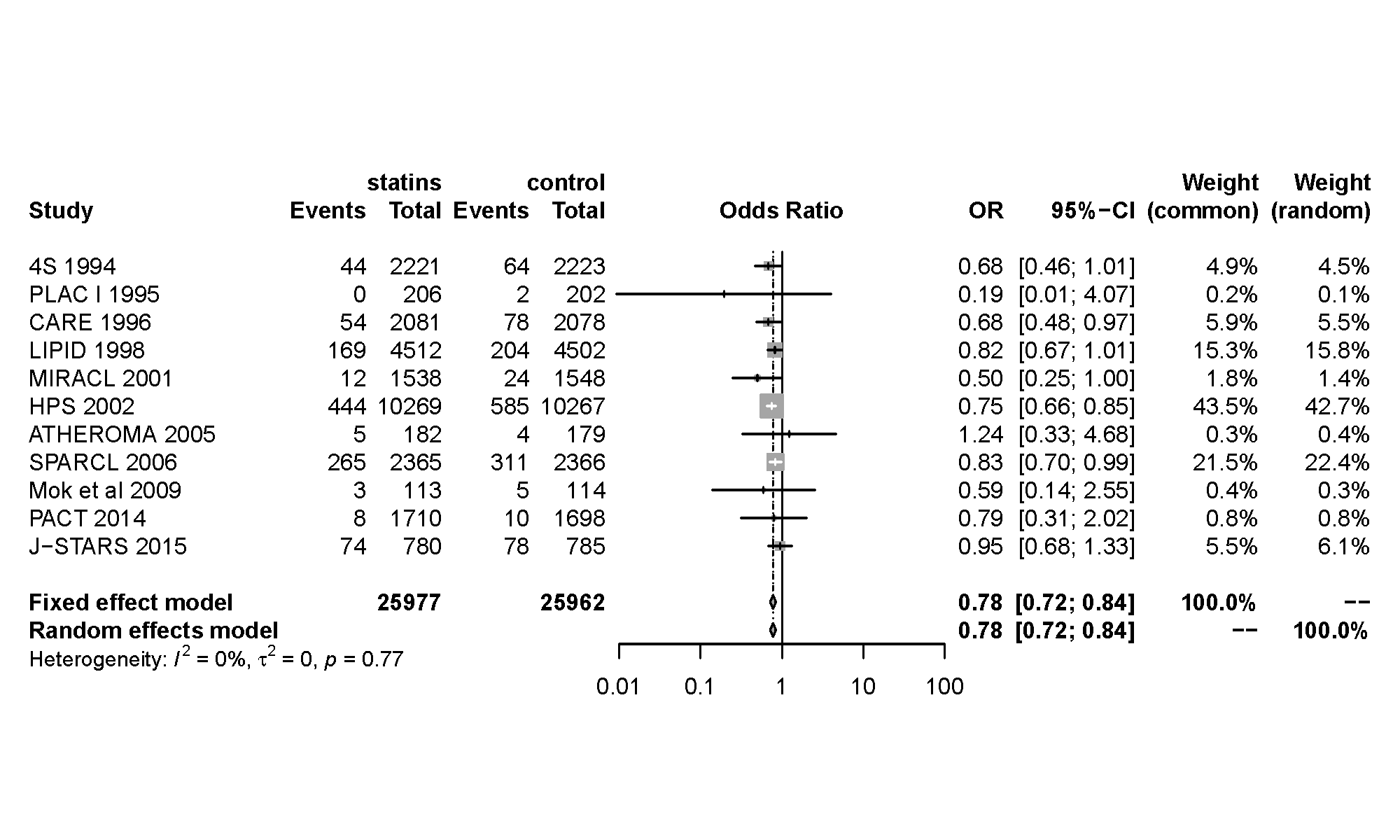


H

Death from cardiovascular diseases


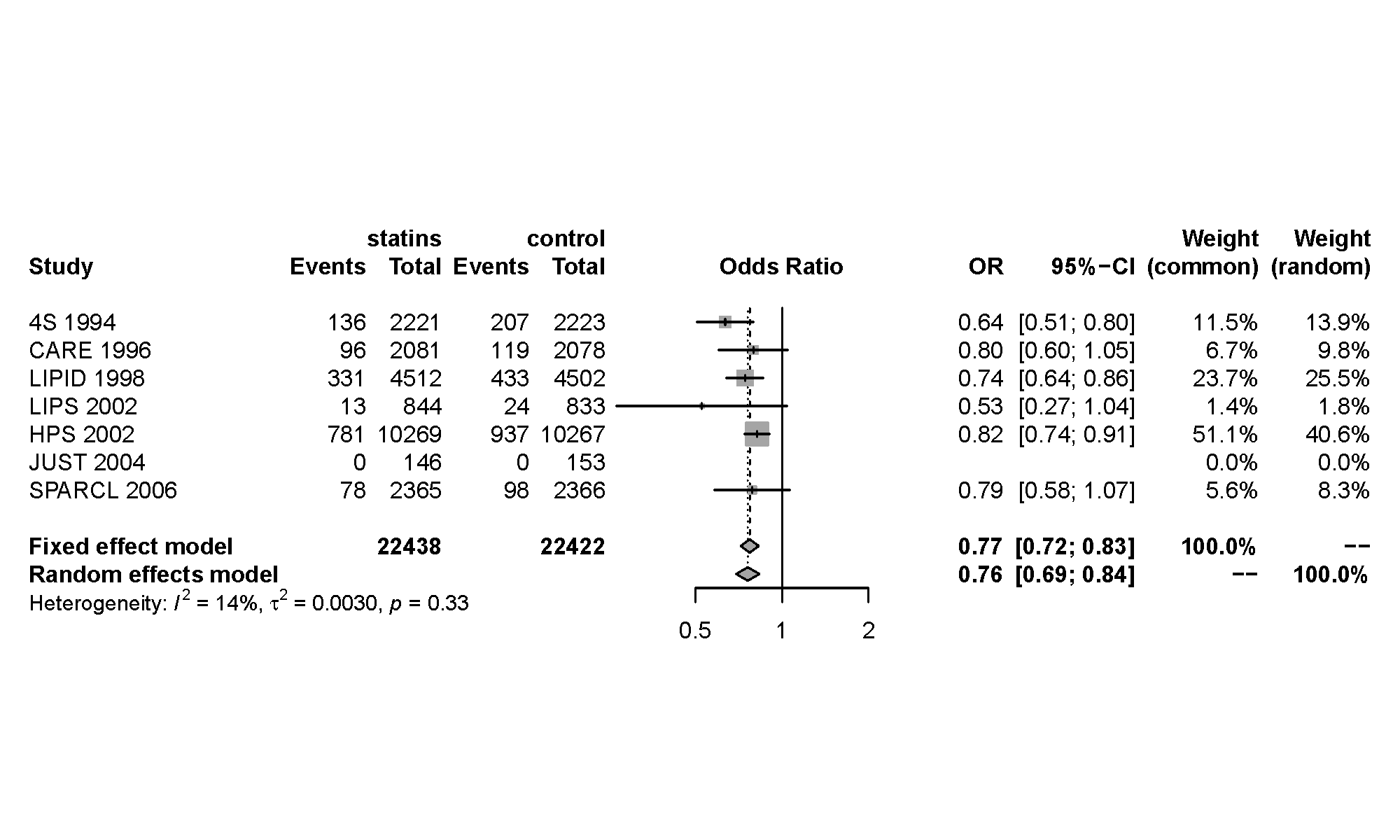


I

All cause death


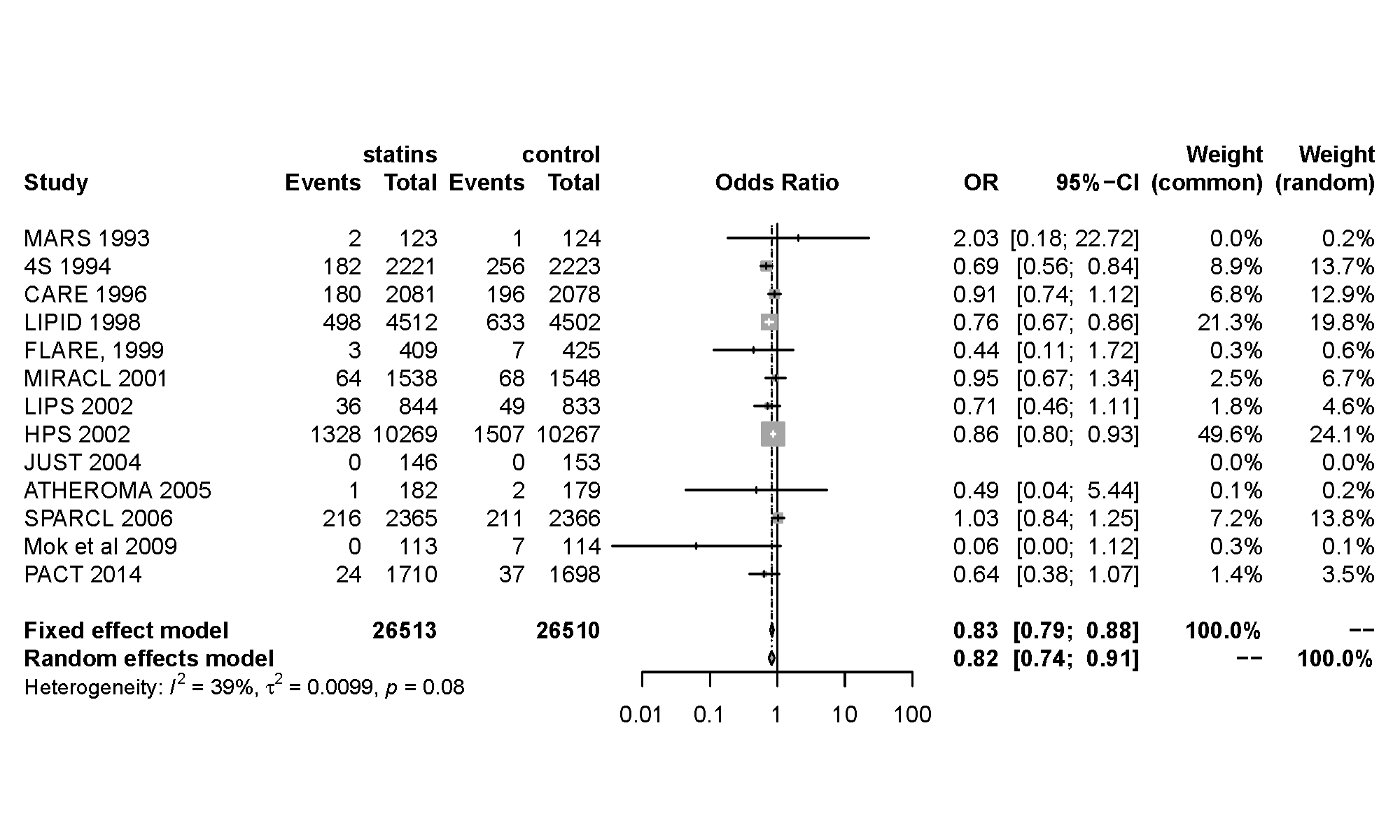


**Figure S2. Leave-one-out influence analyses for pair-wise meta-analyses**


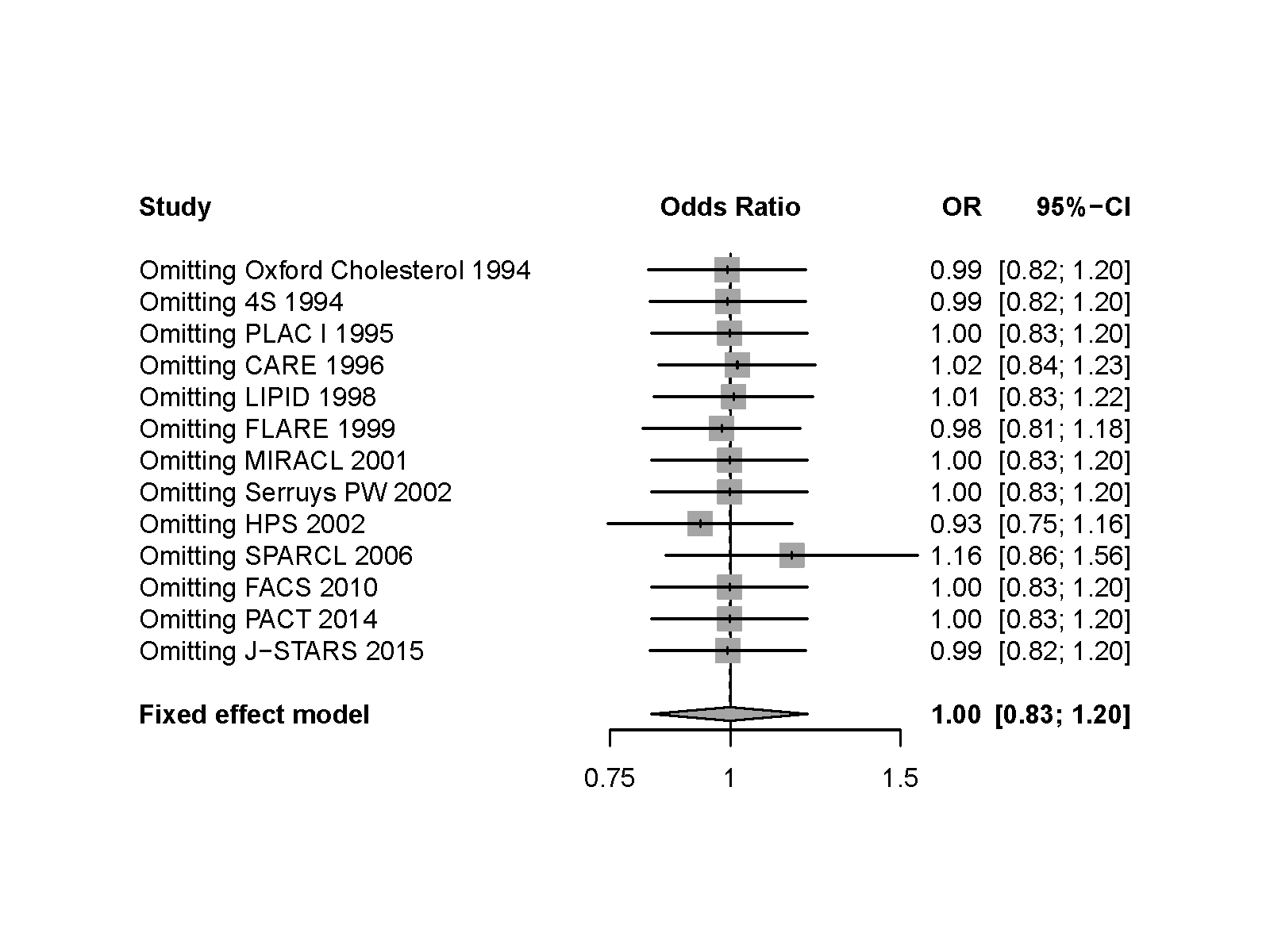


A

Muscle condition

B

Transaminase elevations


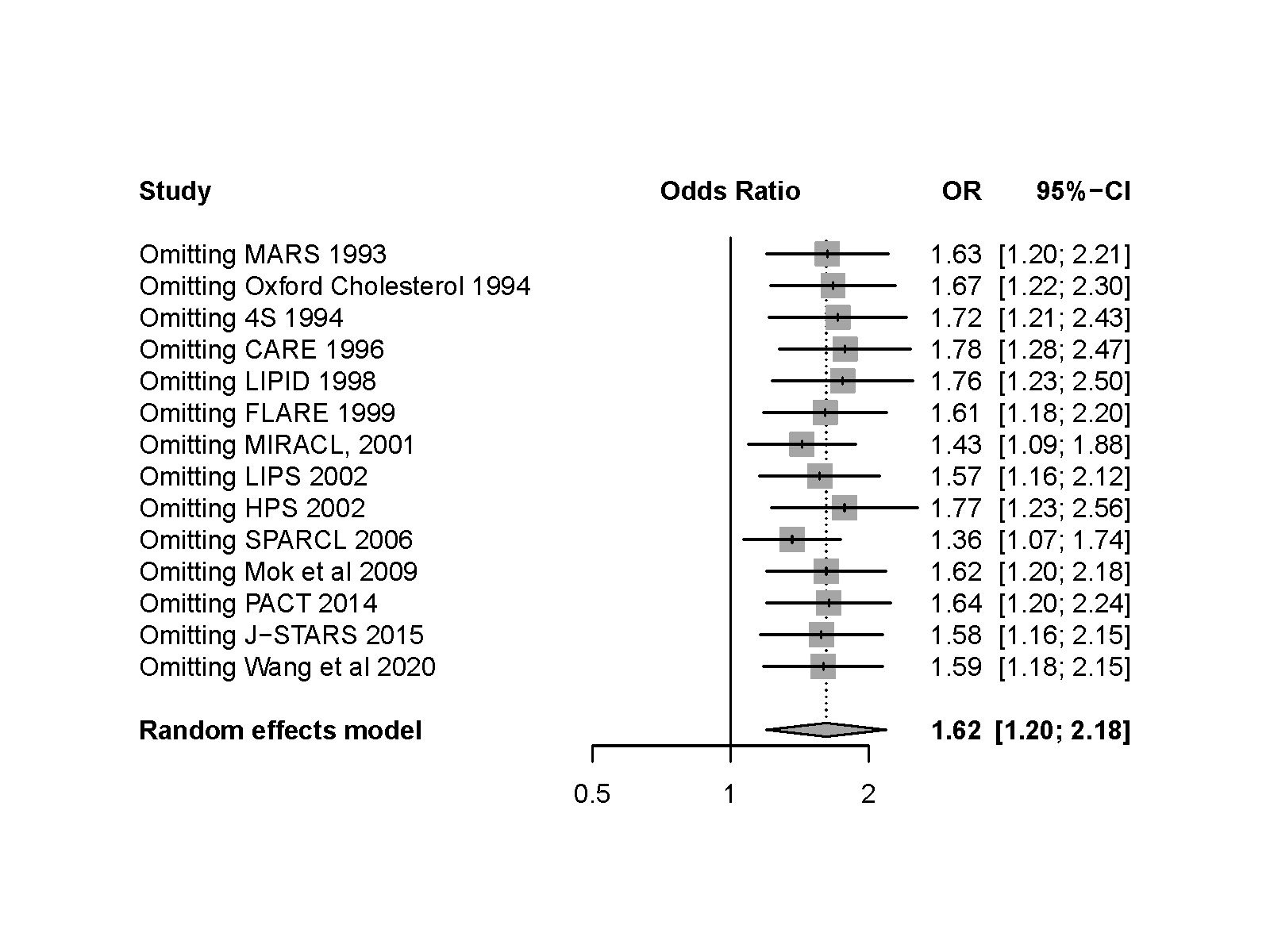


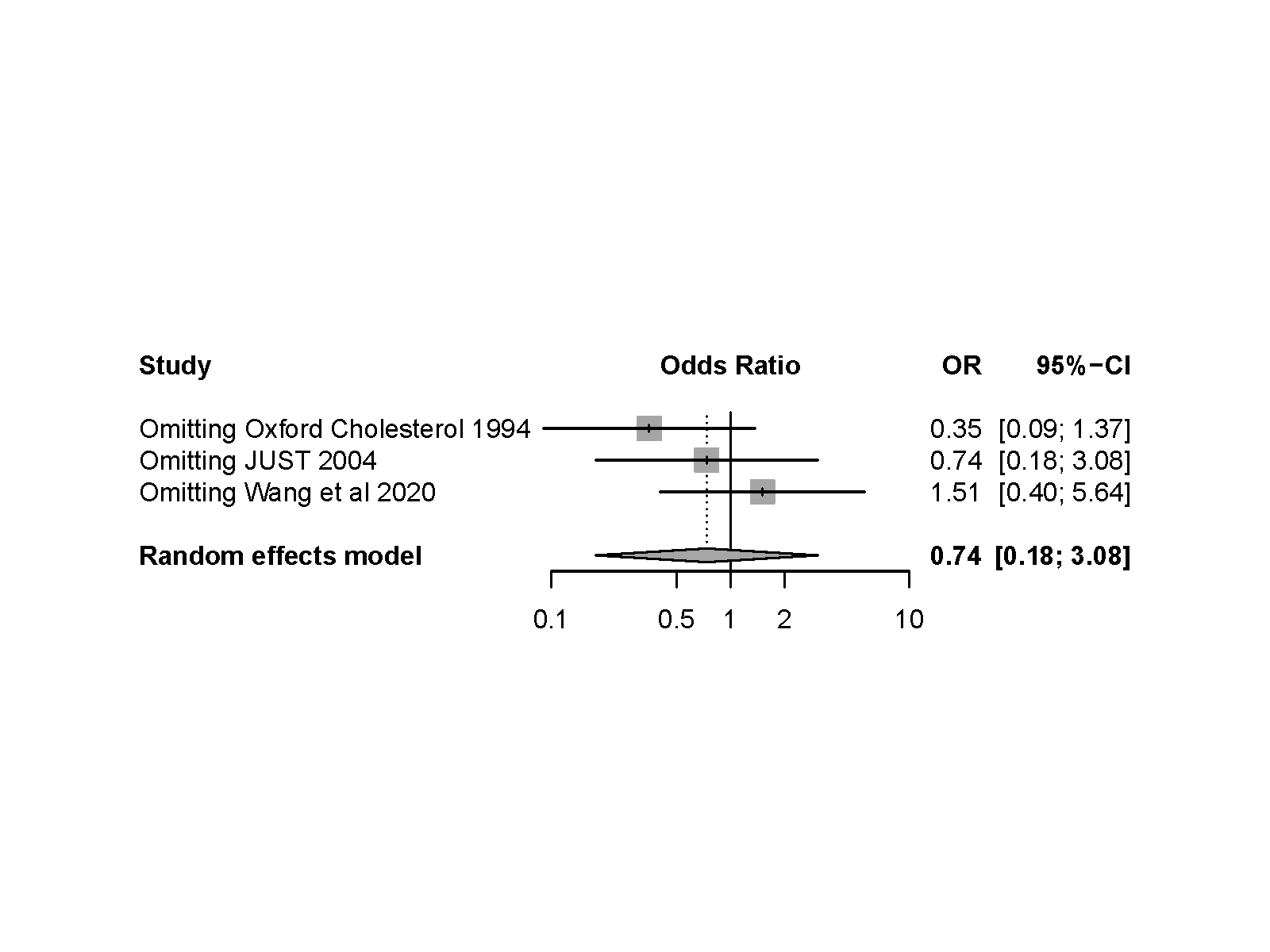


C

Renal Insufficiency

D

Gastrointestinal discomfort


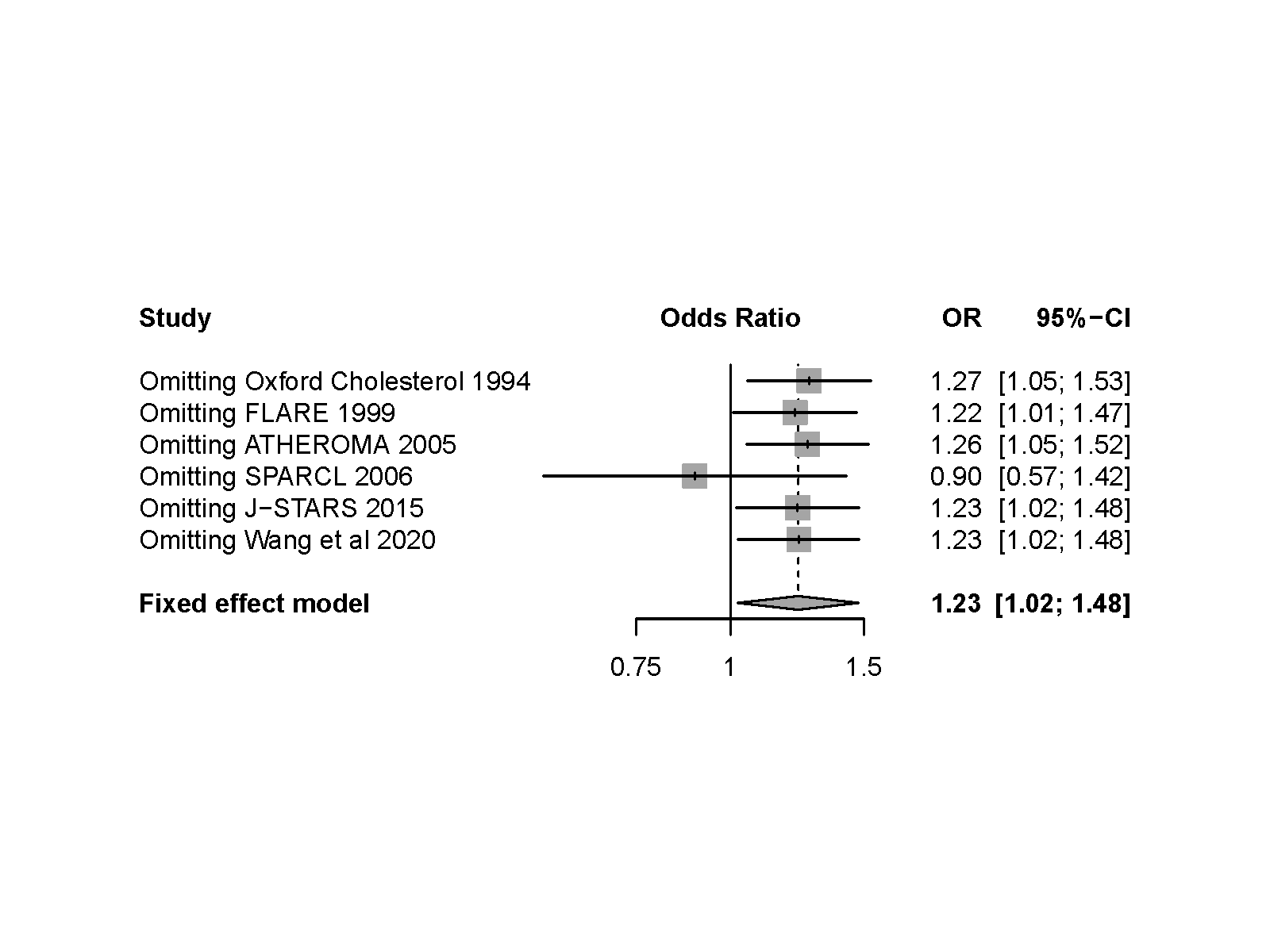


E

Cancer


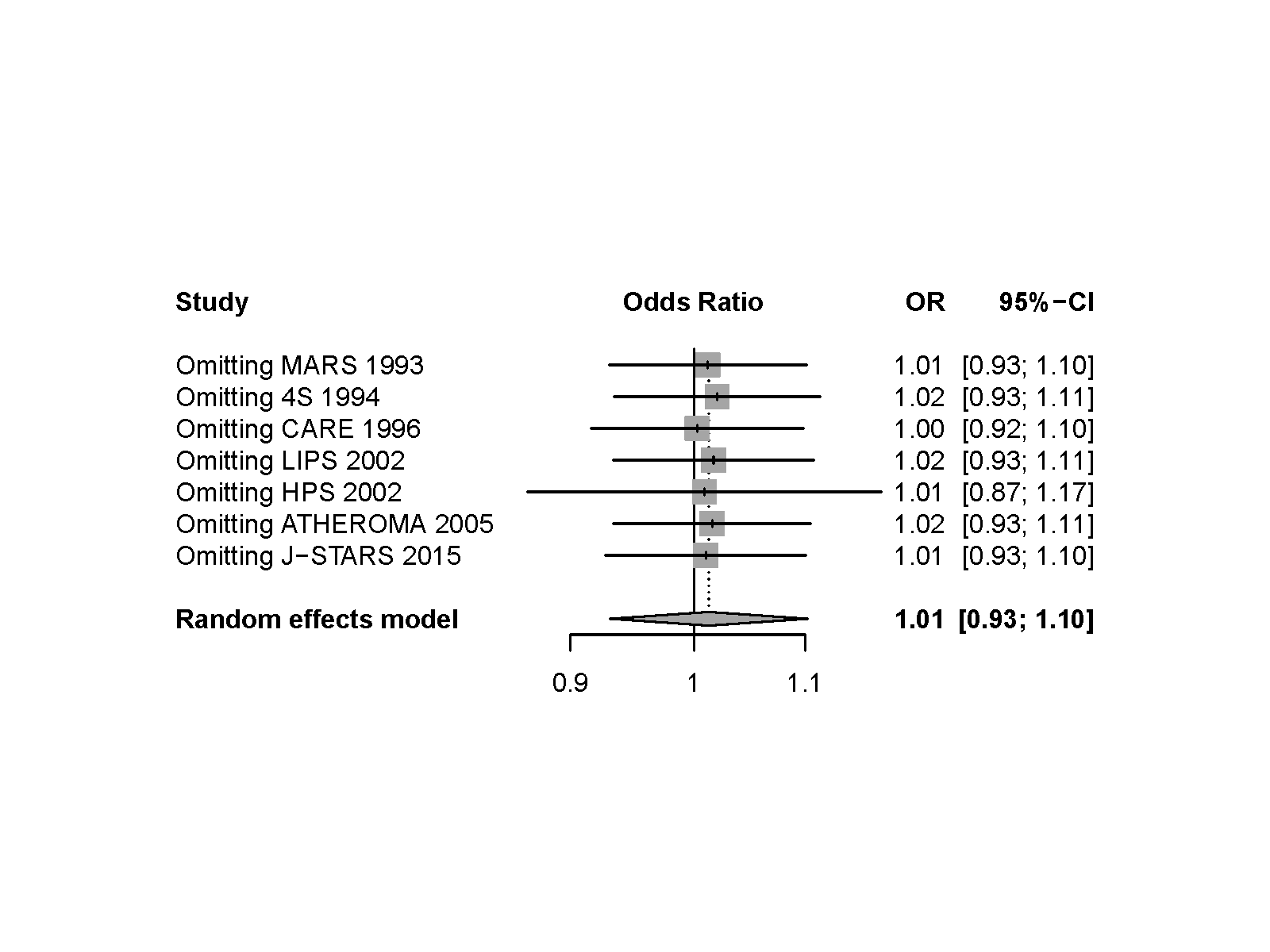


F

Myocardial Infarction


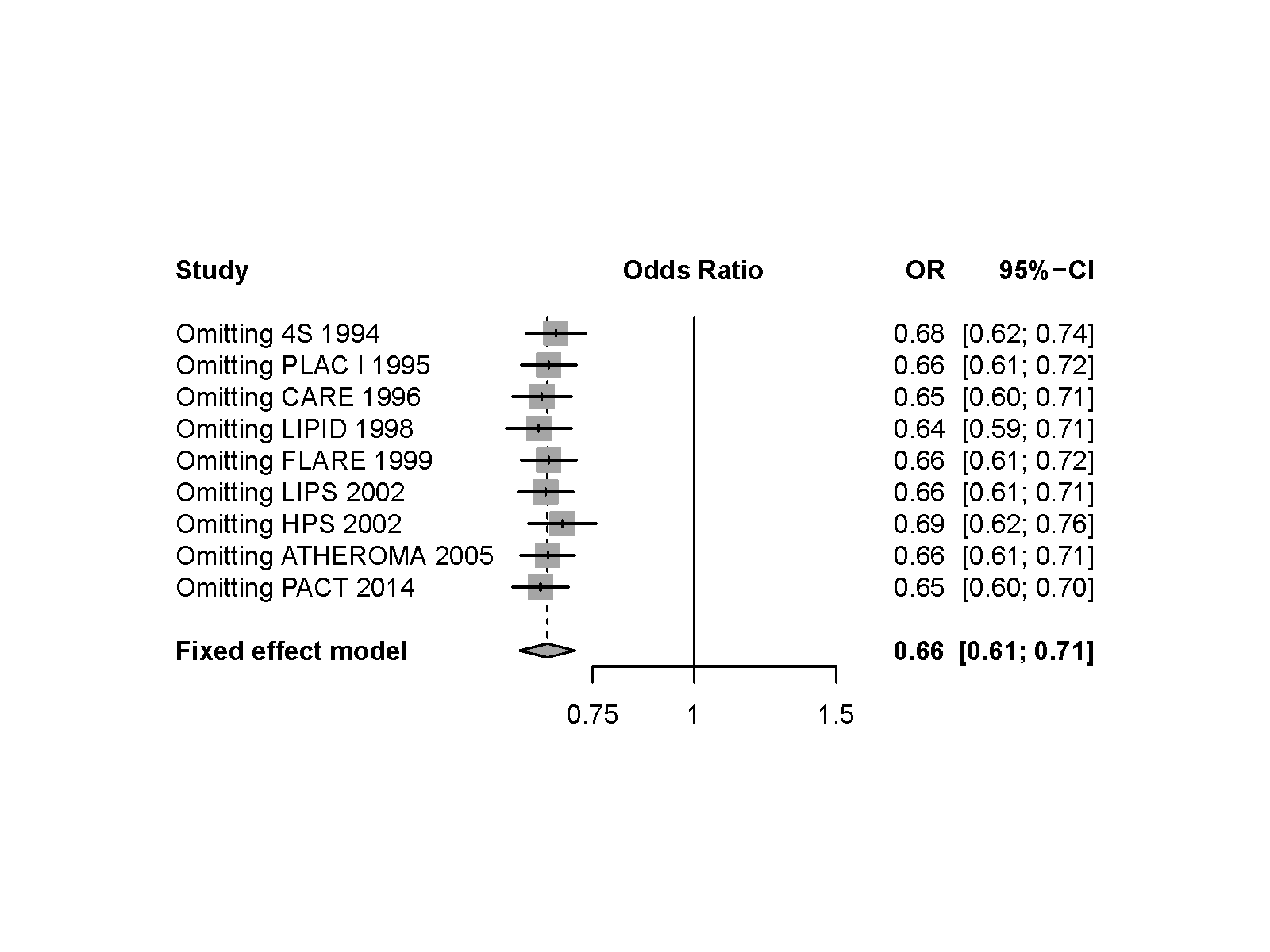


G

Stroke


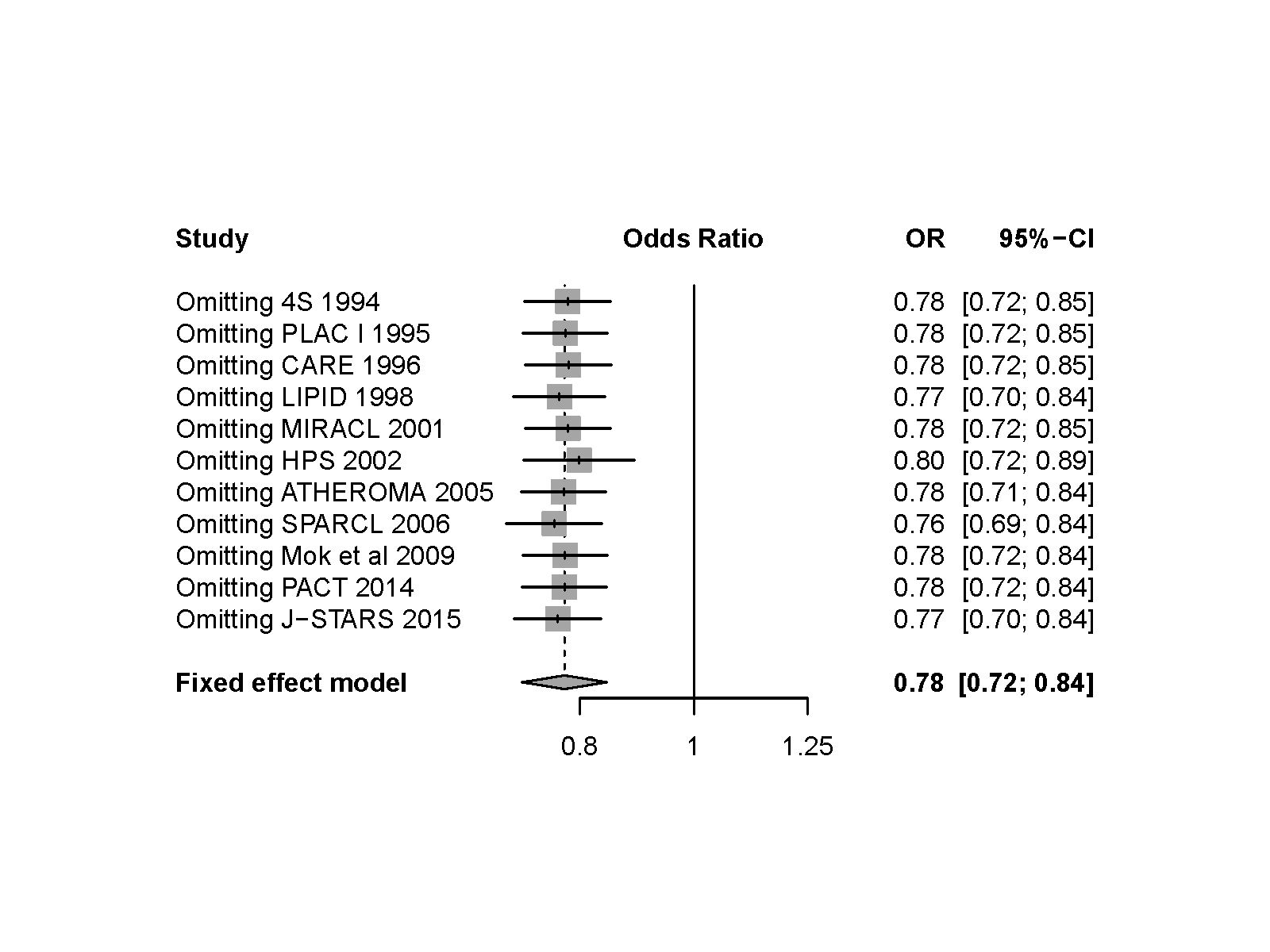


H

Death from cardiovascular diseases


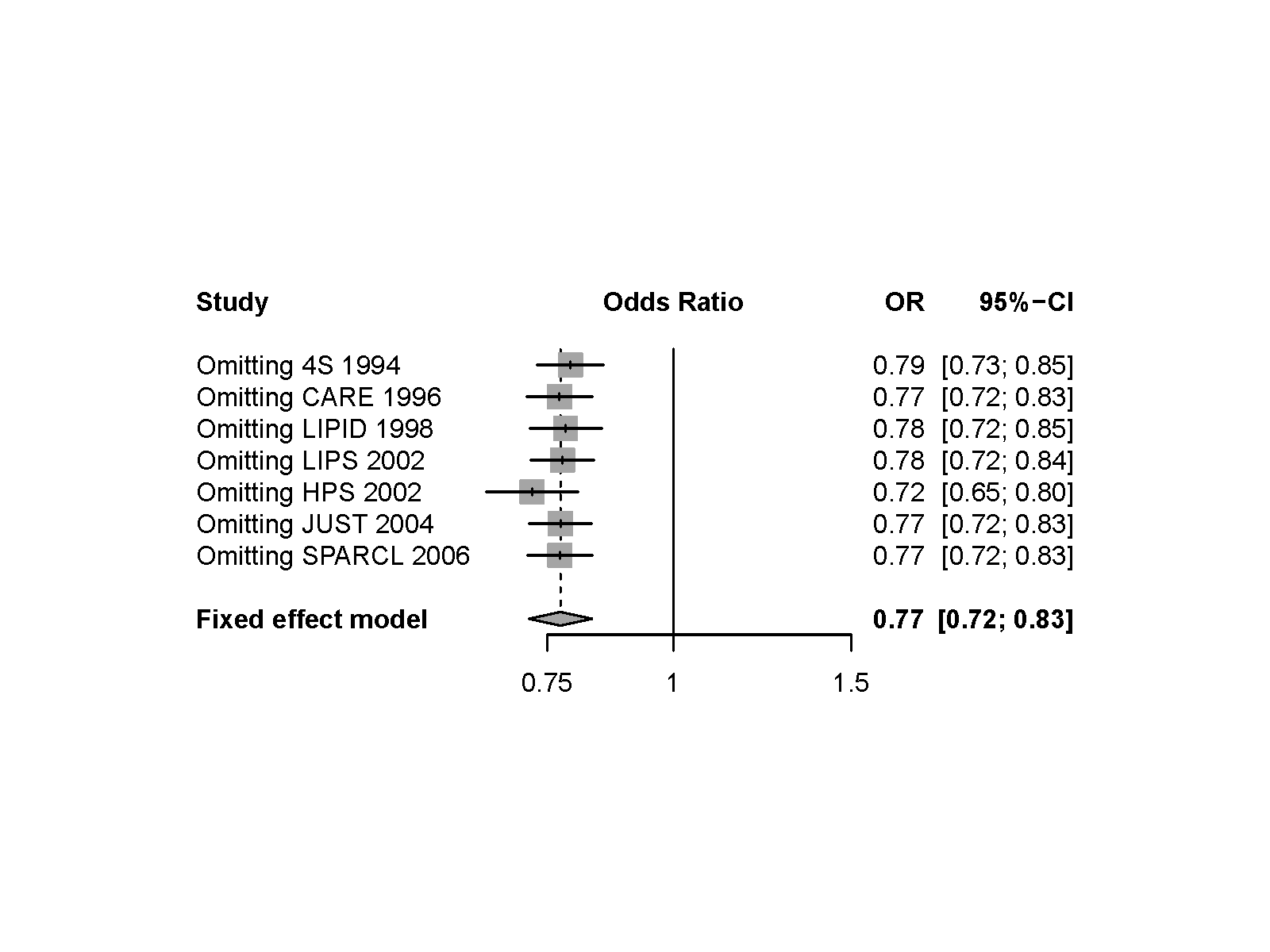


I

All cause death


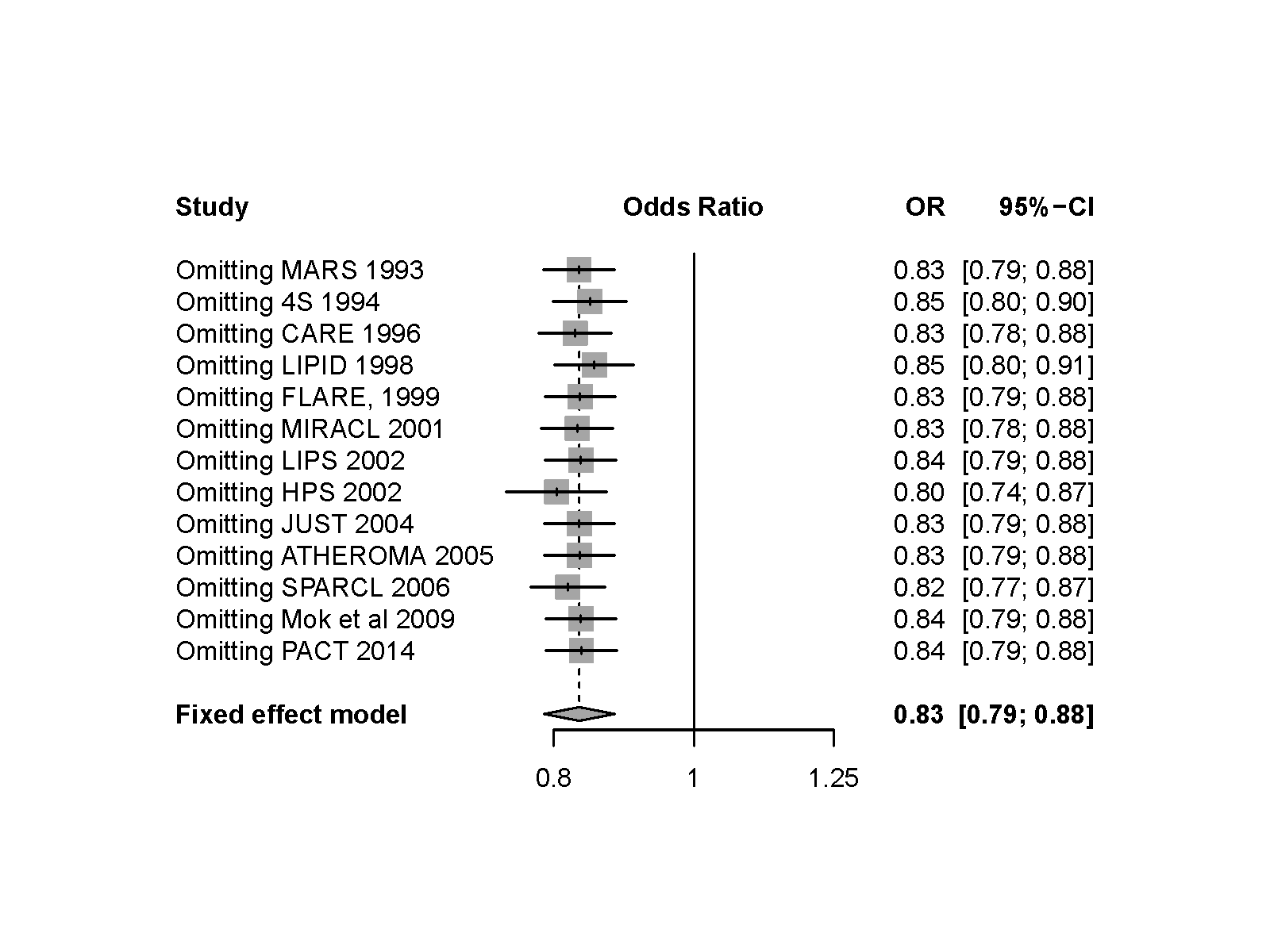


**Figure S3. Funnel plots of publication bias in pair-wise meta-analyses**

A

Muscle condition

Test of funnel plot asymmetry: P = 0.716


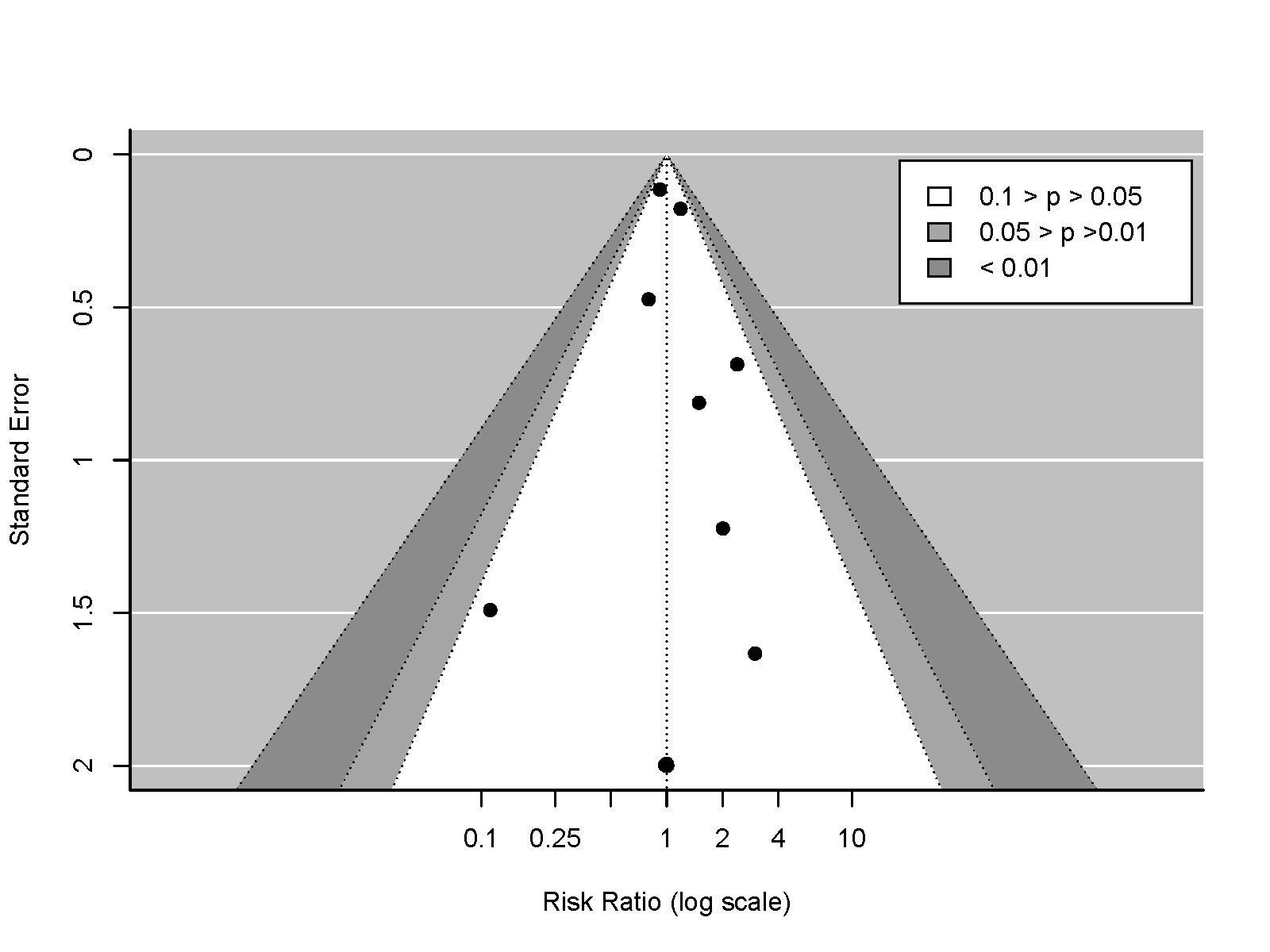


B

Transaminase elevations

Test of funnel plot asymmetry: P = 0.341


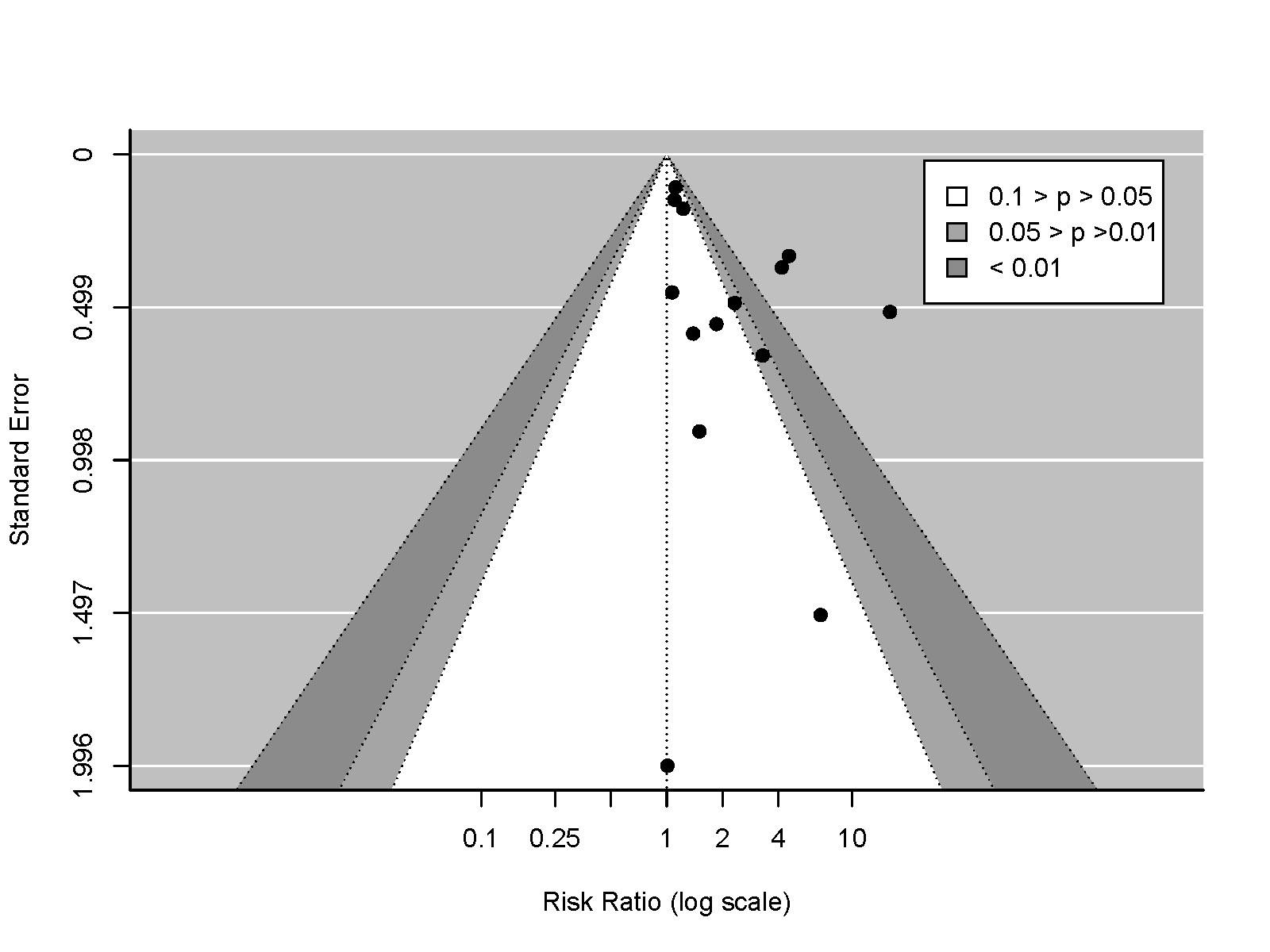


C

Renal Insufficiency

Test of funnel plot asymmetry: P = 0.887


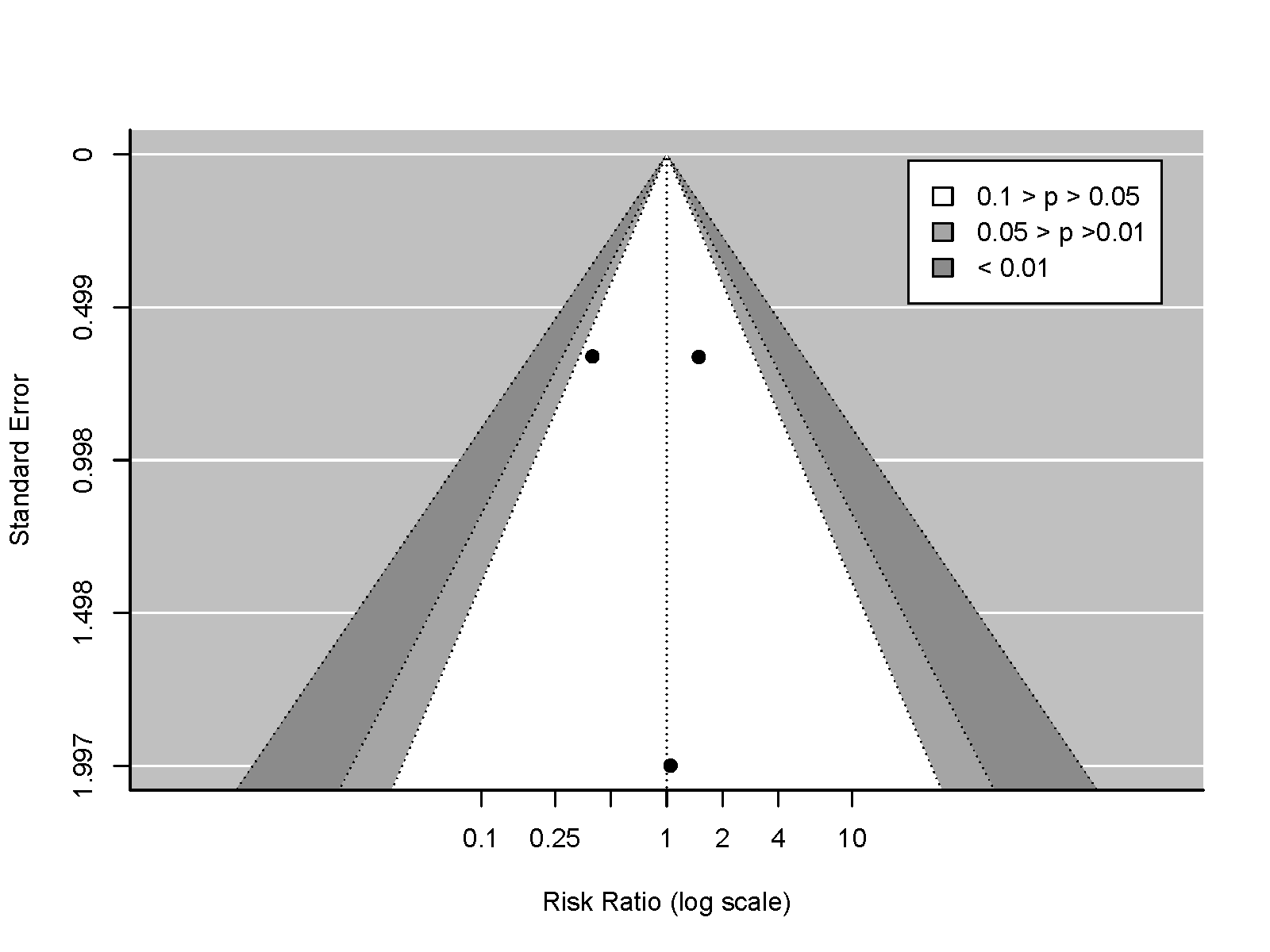


D

Gastrointestinal discomfort

Test of funnel plot asymmetry: P = 0.162


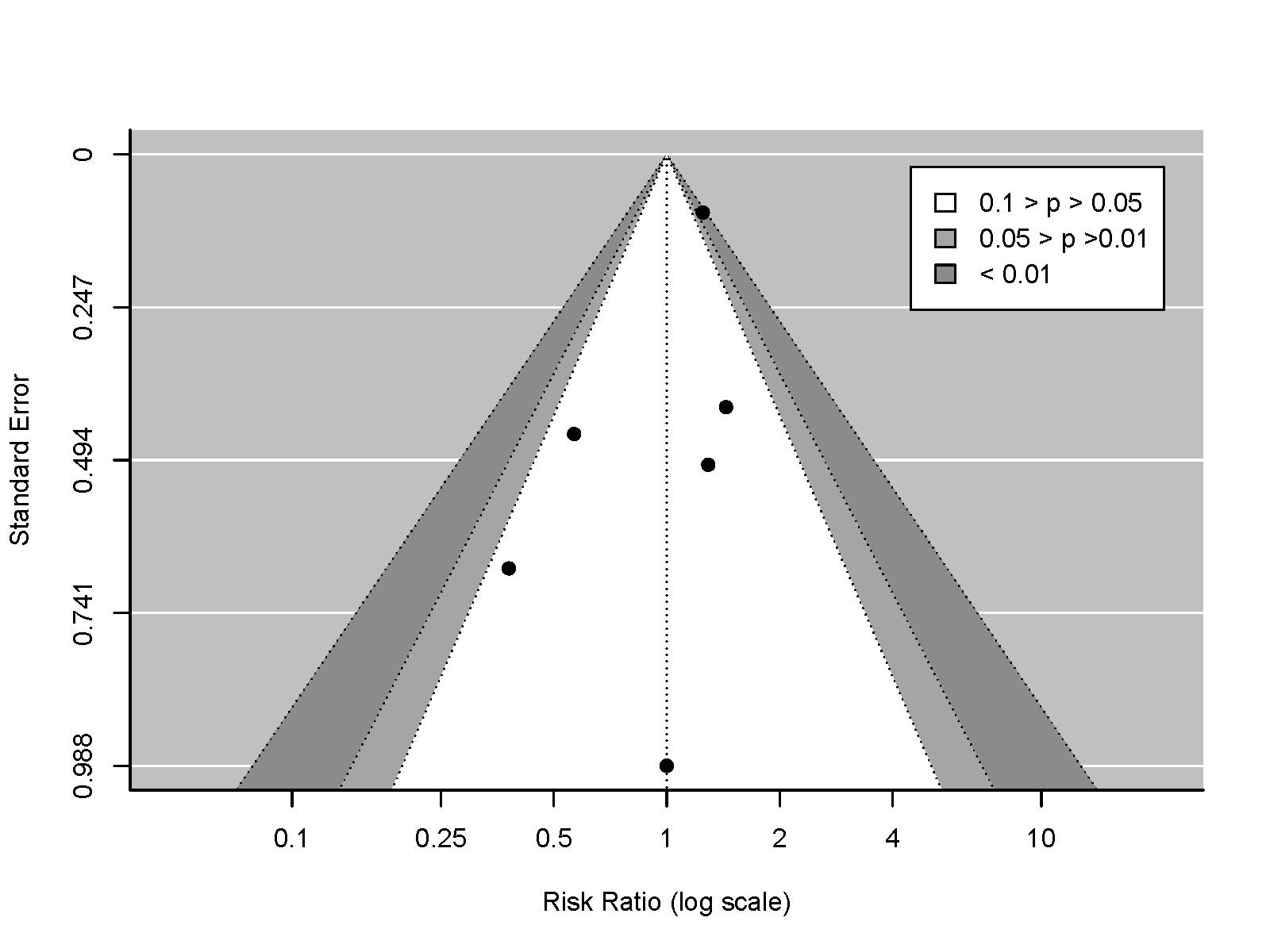


E

Cancer

Test of funnel plot asymmetry: P = 0.668


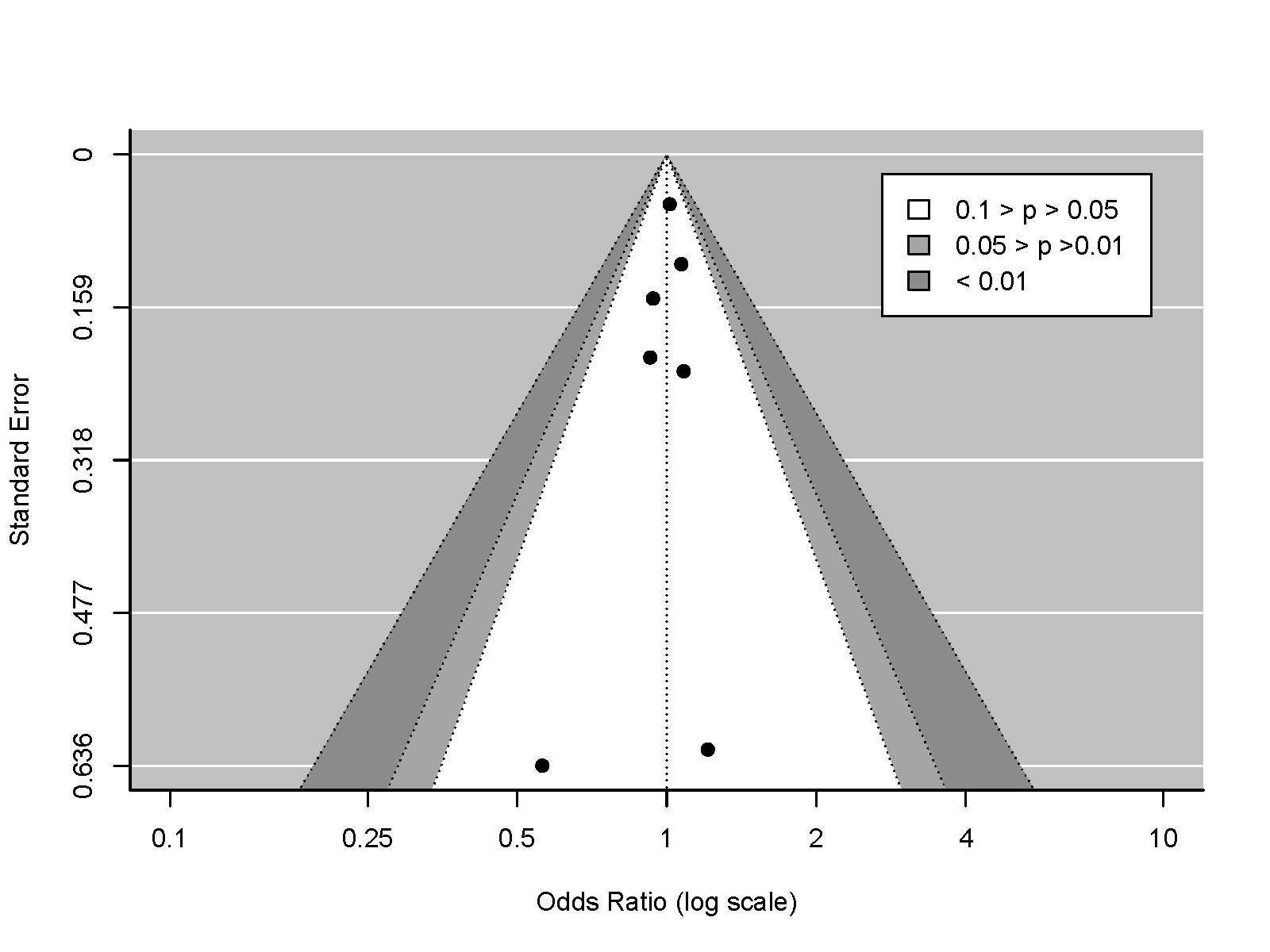


F

Myocardial Infarction

Test of funnel plot asymmetry: P = 0.568


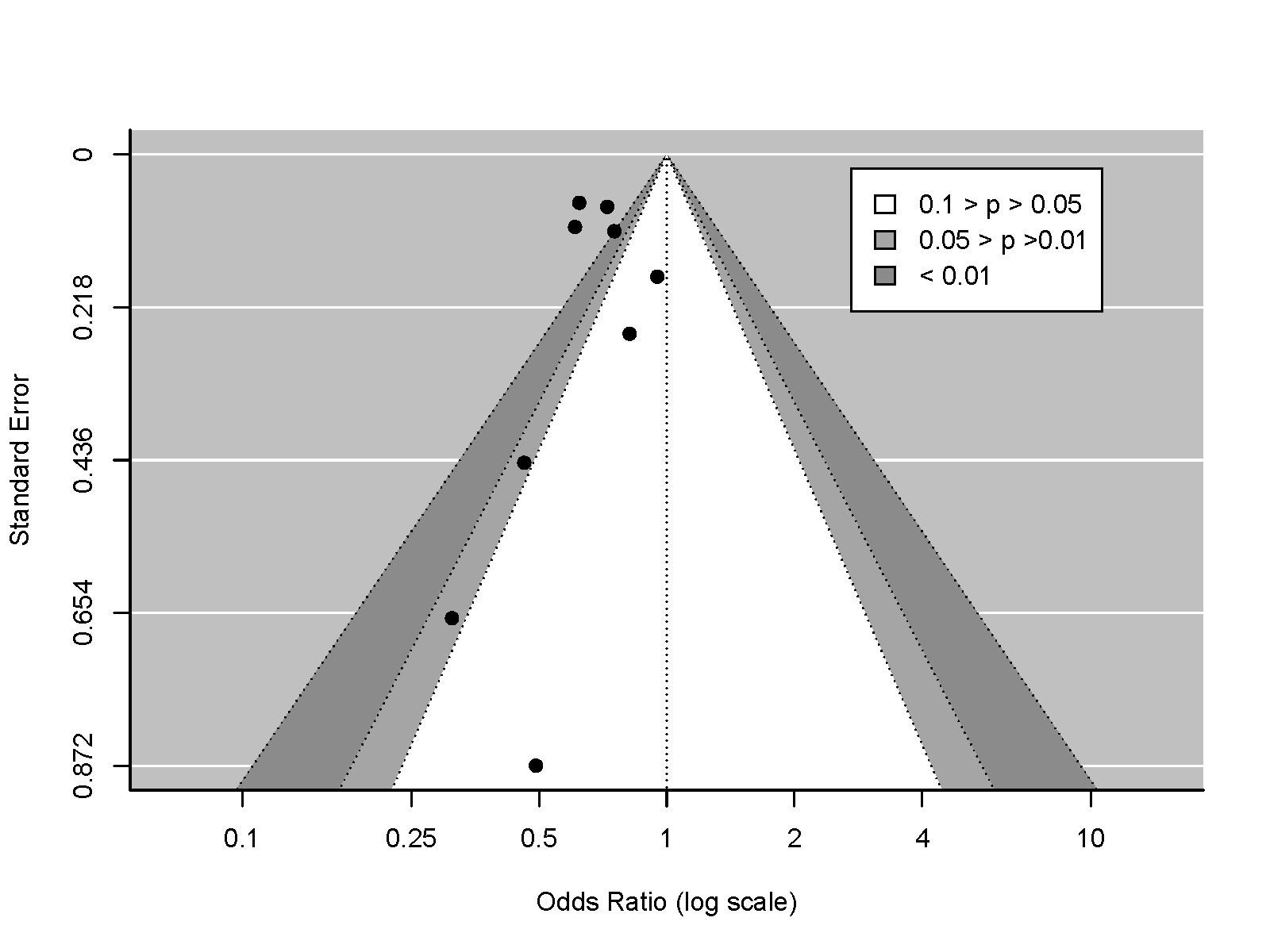


G

Stroke

Test of funnel plot asymmetry: P = 0.489


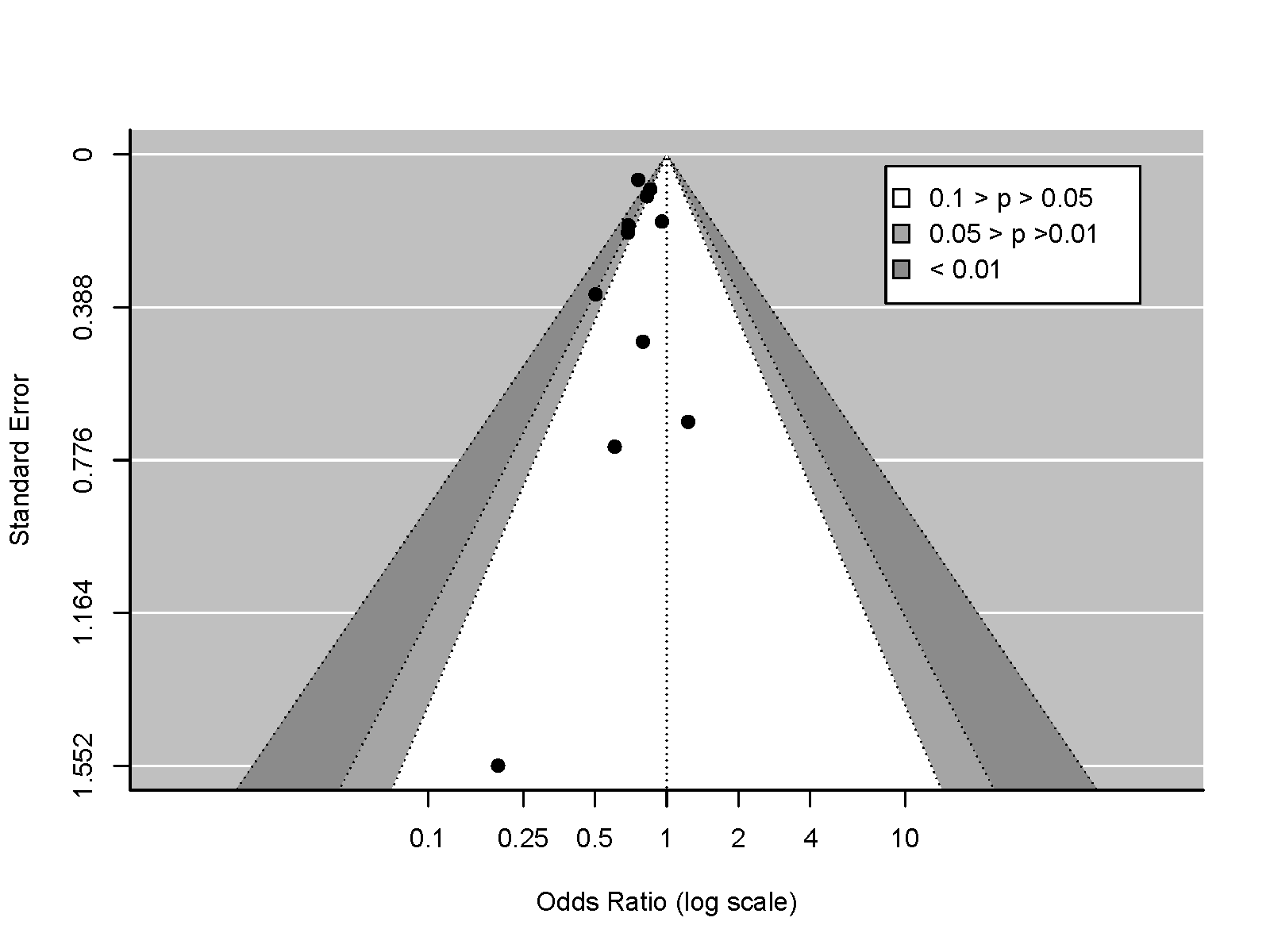


H

Death from cardiovascular diseases

Test of funnel plot asymmetry: P = 0.263


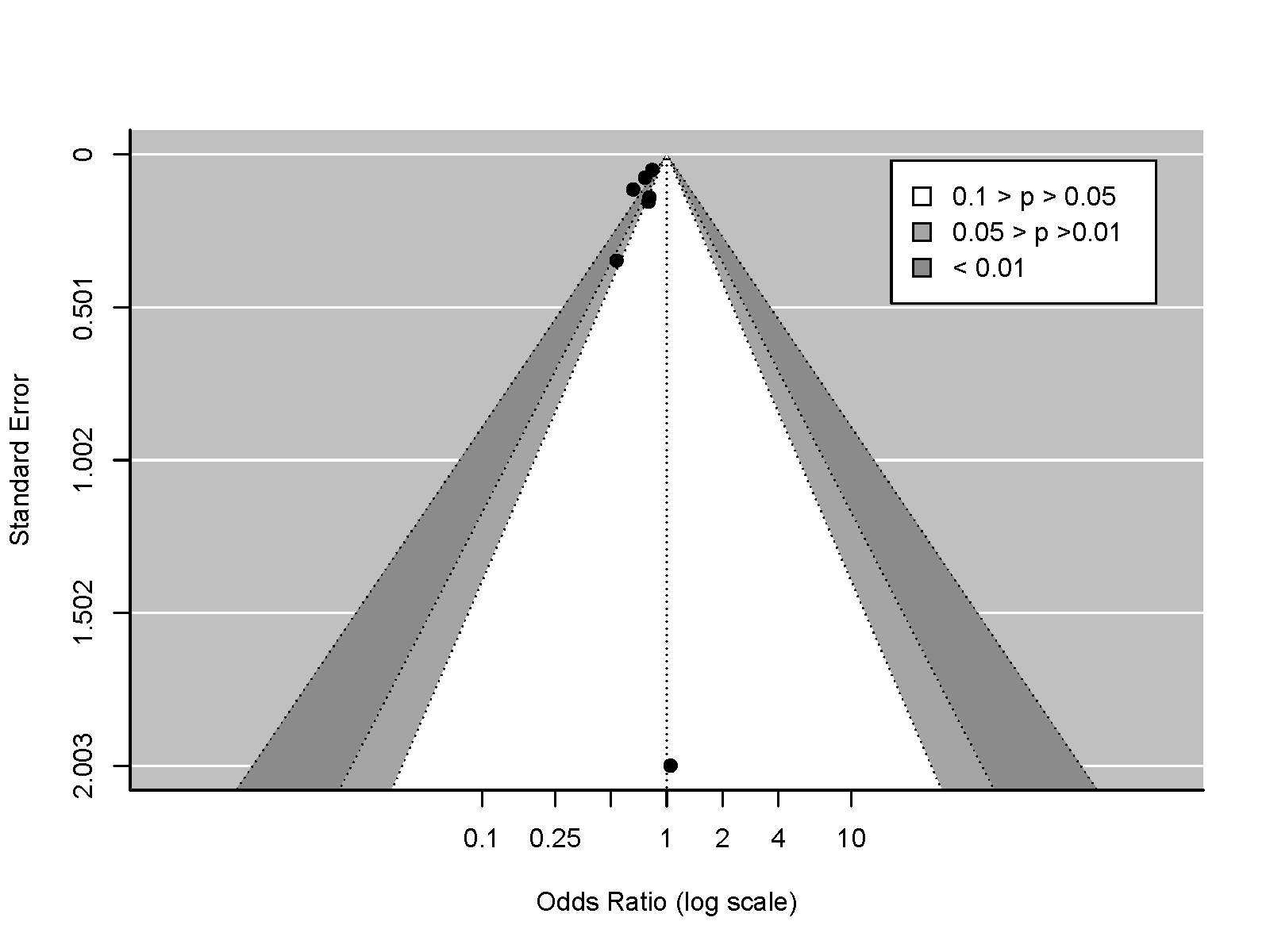


I

All cause death

Test of funnel plot asymmetry: P = 0.243


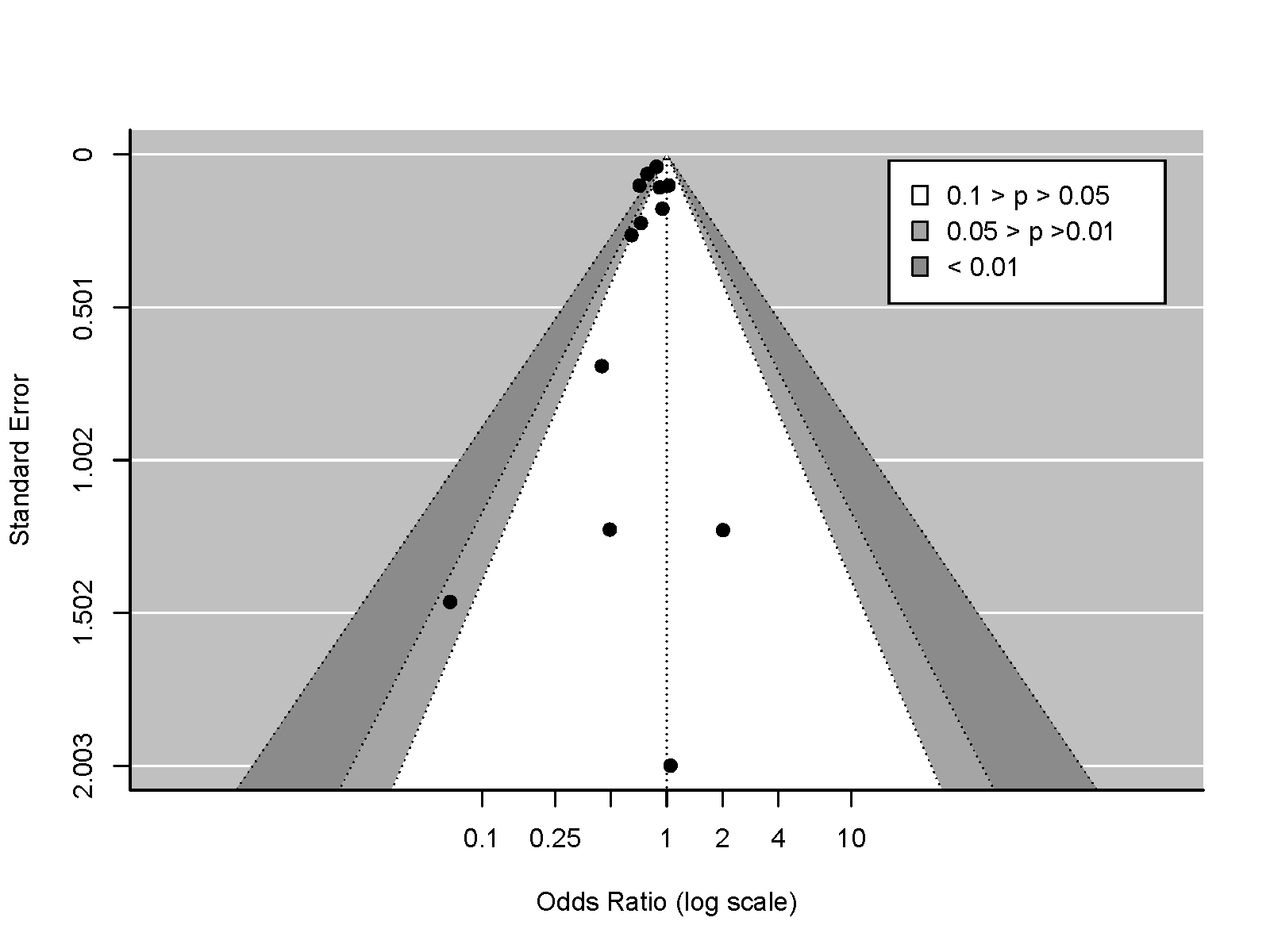


**Table S7. Sensitivity analyses for pair-wise meta-analyses**

| Outcomes | Excluding Nondouble-blind Studies | Excluding Asian populations | Excluding specific studies or individuals ^c^ | Random-effects model |
| --- | --- | --- | --- | --- |
| Muscle condition | 0.99 (0.82 to 1.19) | 0.99 (0.82 to 1.20) | / | 1.02 (0.82 to 1.28) |
| Transaminase elevations | 1.60 (1.16 to 2.23) | 1.55 (1.14 to 2.12) | 1.89 (1.19 to 2.99)^d^ | 1.62 (1.20 to 2.18) |
| Renal insufficiency | /^a^ | 1.51 (0.40 to 5.64)^b^ | / | 0.74 (0.18 to 3.08) |
| Gastrointestinal discomfort | 1.31 (1.08 to 1.60) | 1.27 (1.05 to 1.53) | / | 1.07 (0.73 to 1.57) |
| Cancer | 1.01 (0.93 to 1.10) | 1.01 (0.93 to 1.10) | / | 1.01 (0.93 to 1.10) |
| Myocardial Infarction | 0.66 (0.61 to 0.71) | 0.66 (0.61 to 0.71) | / | 0.67 (0.60 to 0.75) |
| Stroke | 0.77 (0.70 to 0.83) | 0.77 (0.70 to 0.83) | / | 0.78 (0.72 to 0.84) |
| Death from cardiovascular diseases | 0.77 (0.72 to 0.83) | 0.77 (0.72 to 0.83) | / | 0.76 (0.69 to 0.84) |
| All-cause death | 0.83 (0.79 to 0.88) | 0.83 (0.79 to 0.88) | / | 0.82 (0.74 to 0.91) |

^a^ All of the 3 pair-wise studies were not double-blind

^b^ Only one study (keech A, 1994) was included

^c^ We excluded the studies whose indicators did not reach the specified ULN, or the individuals whose indicators in the studies did not reach the specified ULN

^d^ We excluded studies with transaminase elevation < 3 times the ULN, or individuals with transaminase elevation < 3 times the ULN in the study

**Figure S4. Networks of treatment comparisons in network meta-analyses**

A

Transaminase elevations


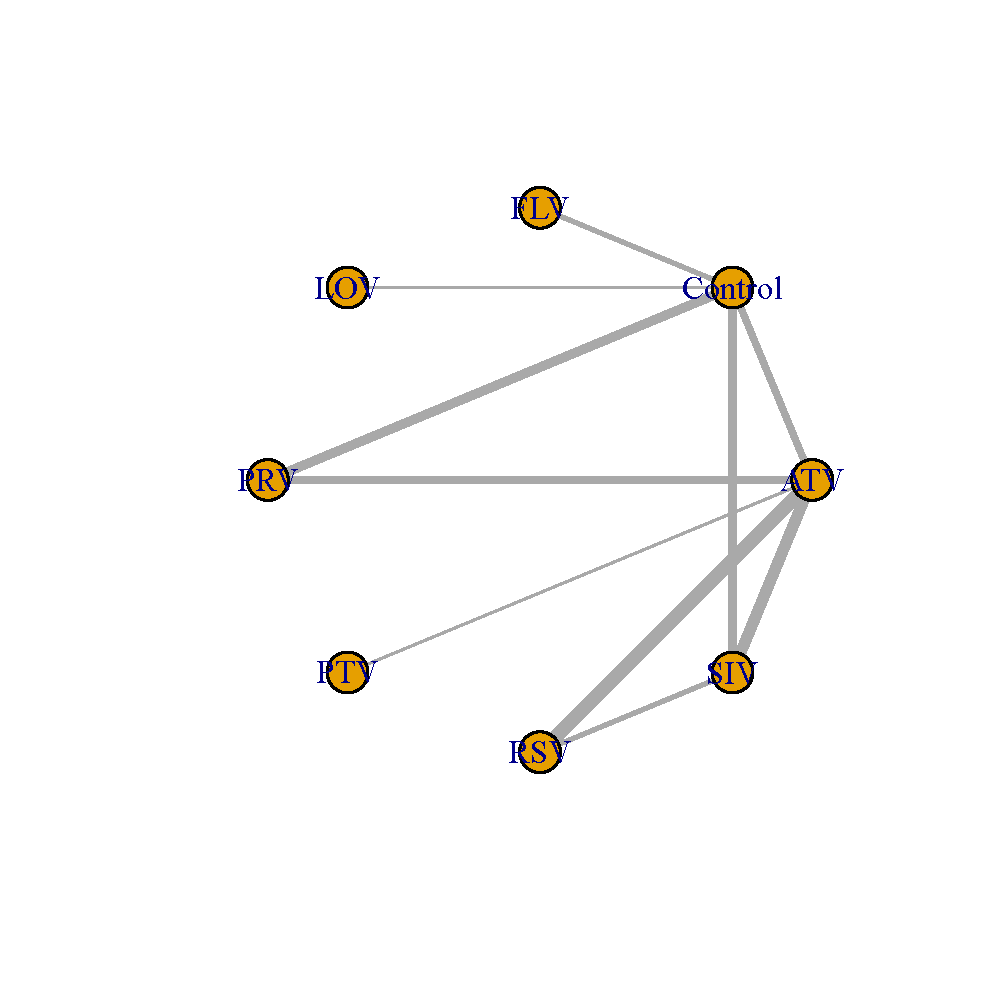


B

Renal Insufficiency


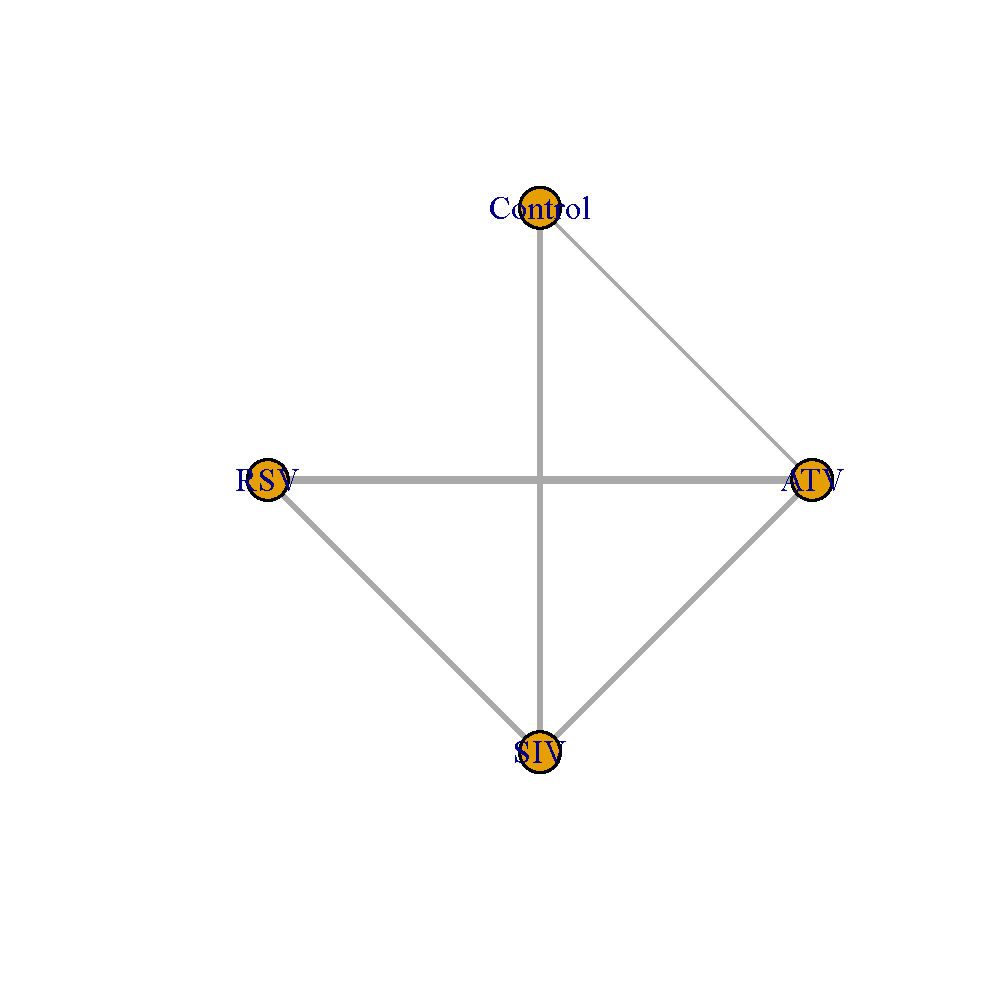


C

Gastrointestinal discomfort

**
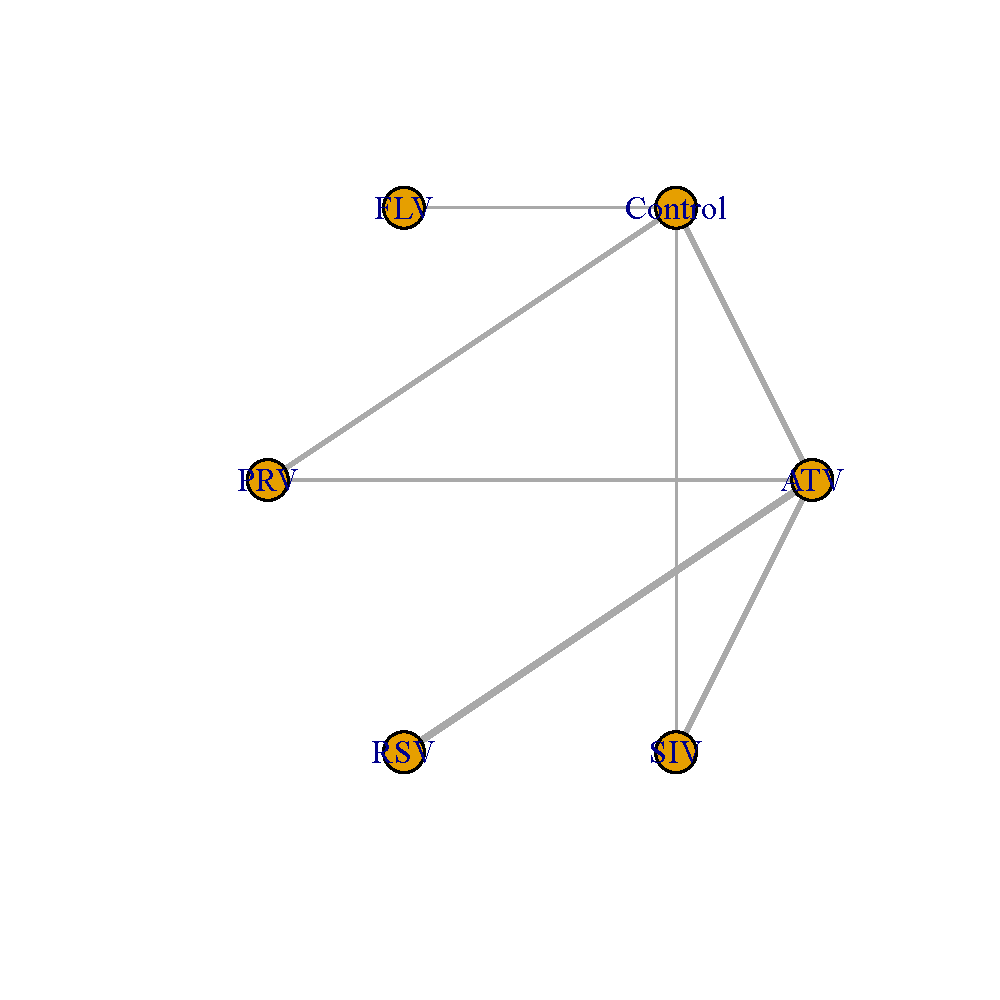
**

D

Cancer

**
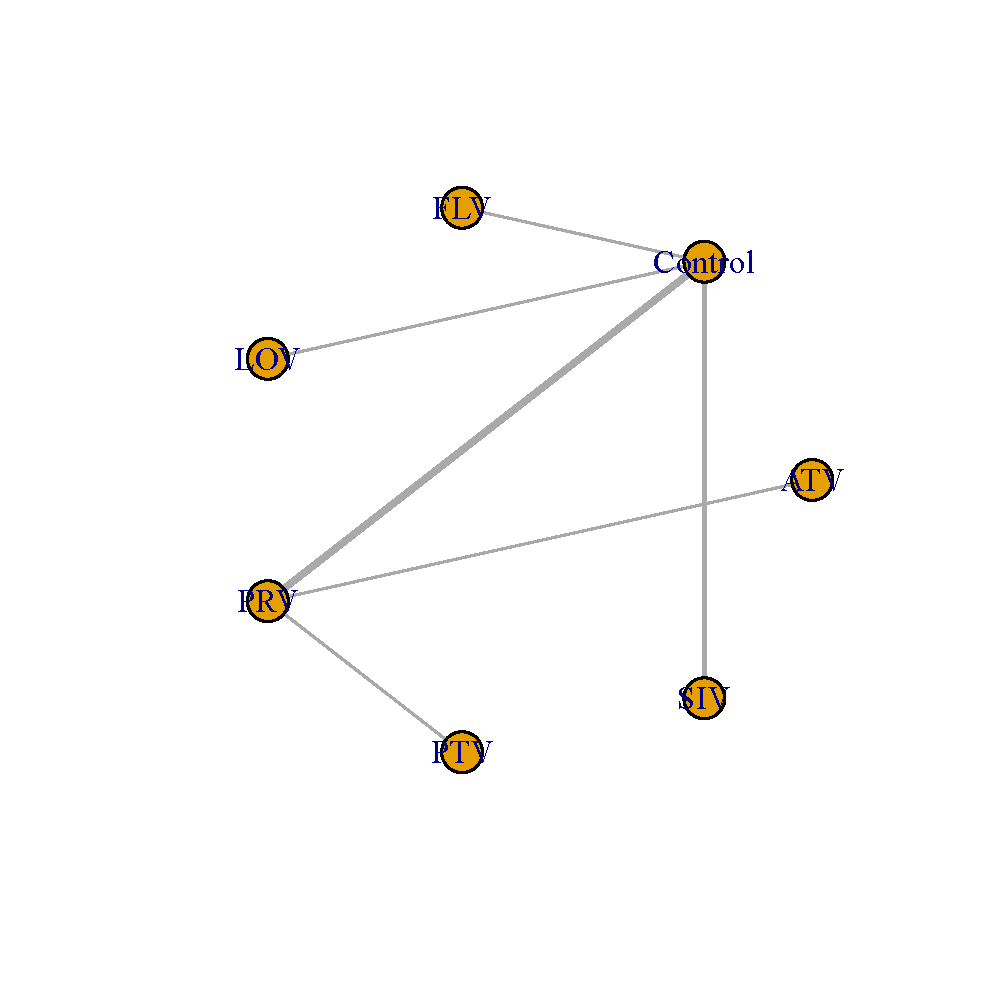
**

**Table S8. Node-splitting analyses of inconsistency between direct and indirect evidence in network meta-analyses**

| **Transaminase elevations** | | | | | |
| --- | --- | --- | --- | --- | --- |
| **Treatment** | **Comparator** | **Direct** | **Indirect** | **Network** | **P** |
| Atorvastatin | Control | 0.18 (0.06, 0.50) | 0.30 (0.12, 0.72) | 0.25 (0.14, 0.46) | 0.47 |
| Atorvastatin | Pravastatin | 0.25 (0.09, 0.63) | 0.33 (0.12, 1.09) | 0.30 (0.15, 0.56) | 0.67 |
| Atorvastatin | Rosuvastatin | 0.71 (0.27, 1.52) | 0.08 (0.00, 1.19) | 0.57 (0.22, 1.22) | 0.14 |
| Atorvastatin | Simvastatin | 0.48 (0.18, 1.38) | 0.30 (0.10, 0.91) | 0.90 (0.20, 0.76) | 0.52 |
| Pravastatin | Control | 1.25 (0.59, 2.72) | 0.95 (0.25, 3.00) | 1.16 (0.63, 2.16) | 0.68 |
| Simvastatin | Control | 1.16 (0.49, 2.66) | 2.61 (0.87,9.97) | 1.46 (0.82, 3.00) | 0.24 |
| Rosuvastatin | Simvastatin | 1.34 (0.22, 9.03) | 0.51 (0.18, 1.97) | 0.65 (0.25, 2.01) | 0.40 |
| **Renal insufficiency** | | | | | |
| **Treatment** | **Comparator** | **Direct** | **Indirect** | **Network** | **P** |
| Atorvastatin | Control | 3.00 (0.57, 20.09) | 0.33 (0.03, 2.72) | 1.28 (0.33, 4.95) | 0.10 |
| Atorvastatin | Rosuvastatin | 0.90 (0.25, 3.00) | 5.47 (0.37, 99.48) | 1.12 (0.33, 3.32) | 0.21 |
| Atorvastatin | Simvastatin | 0.44 (0.04, 3.32) | 1.19 (0.22, 6.69) | 0.89 (0.25, 3.00) | 0.45 |
| Simvastatin | Control | 1.55 (0.30, 11.02) | 0.17 (0.01, 1.60) | 0.69 (0.17, 2.66) | 0.11 |
| Rosuvastatin | Simvastatin | 0.64 (0.20, 1.63) | 9.97 (0.42, 221.40) | 0.79 (0.27, 2.20) | 0.09 |
| **Gastrointestinal discomfort** | | | | | |
| **Treatment** | **Comparator** | **Direct** | **Indirect** | **Network** | **P** |
| Atorvastatin | Control | 0.79 (0.20, 3.67) | 1.02 (0.20, 7.39) | 0.84 (0.37, 2.84) | 0.79 |
| Atorvastatin | Pravastatin | 1.75 (0.22, 14.88) | 0.56 (0.10, 3.00) | 0.81 (0.25, 2.00) | 0.33 |
| Atorvastatin | Simvastatin | 0.33 (0.10, 1.45) | 0.51 (0.06, 6.05) | 0.37 (0.15, 1.11) | 0.68 |
| Pravastatin | Control | 0.73 (0.18, 2.66) | 2.36 (0.22, 27.11) | 0.95 (0.30, 2.72) | 0.33 |
| Simvastatin | Control | 0.55 (0.08, 3.37) | 0.37 (0.06, 2.14) | 0.41 (0.14, 1.34) | 0.66 |

Since direct and indirect comparisons of cancer did not yield a closed loop, the node-splitting analysis of inconsistency was not performed.

**Table S9. Results of direct** **comparisons of different interventions for muscle condition**

| **Comparation** | **No of studies** | **Odds ratio (95%CI)** | **P value** |
| --- | --- | --- | --- |
| Compared to controls | | | |
| Pravastatin | 5 | 0.90 (0.38 to 2.14) | 0.81 |
| Atorvastatin | 2 | 0.91 (0.72 to 1.15) | 0.43 |
| Fluvastatin | 3 | 2.45 (0.63 to 9.54) | 0.20 |
| Simvastatin | 3 | 1.22 (0.87 to 1.71) | 0.25 |
| Compared to atorvastatin | | | |
| Simvastatin | 4 | 0.70 (0.55 to 0.90) | 0.006 |
| Rosuvastatin | 6 | 1.75 (1.17 to 2.61) | 0.006 |
| Pravastatin | 3 | 0.88 (0.65 to 1.19) | 0.40 |
| Pitavastatin | 1 | 0.34 (0.01 to 8.30) | 0.50 |
| Compared to pitavastatin | | | |
| Pravastatin | 1 | 2.03 (0.18 to 22.83) | 0.57 |

**Table S10. Comparative adverse effects between different statin types from random-effect network meta-analyses**

| **Transaminase elevations** | | | | | | | | | | | | | | | | | | |
| --- | --- | --- | --- | --- | --- | --- | --- | --- | --- | --- | --- | --- | --- | --- | --- | --- | --- | --- |
| **Atorvastatin** | 0.25 (0.13, 0.46) | | | 0.64 (0.16, 2.62) | | | 0.41 (0.04, 5.24) | | | 0.29 (0.14, 0.56) | | 1.01 (0.20, 5.20) | | | 0.57 (0.23, 1.22) | | | 0.36 (0.20, 0.76) |
| 4.05 (2.18, 7.60) | **Control** | | | 2.58 (0.77, 9.22) | | | 1.64 (0.16, 19.75) | | | 1.16 (0.63, 2.15) | | 4.08 (0.72, 23.66) | | | 2.30 (0.79, 5.83) | | | 1.45 (0.82, 3.10) |
| 1.56 (0.38, 6.10) | 0.39 (0.11, 1.29) | | | **Fluvastatin** | | | 0.63 (0.05, 10.02) | | | 0.45 (0.11, 1.73) | | 1.57 (0.18, 13.38) | | | 0.89 (0.17, 4.00) | | | 0.57 (0.15, 2.40) |
| 2.46 (0.19, 26.74) | 0.61 (0.05, 6.10) | | | 1.58 (0.10, 21.44) | | | **Lovastatin** | | | 0.71 (0.05, 7.63) | | 2.47 (0.12, 44.81) | | | 1.39 (0.09, 16.21) | | | 0.90 (0.07, 10.27) |
| 3.49 (1.77, 6.92) | 0.86 (0.46, 1.59) | | | 2.23 (0.58, 9.12) | | | 1.42 (0.13, 18.29) | | | **Pravastatin** | | 3.51 (0.61, 20.82) | | | 1.98 (0.64, 5.25) | | | 1.25 (0.60, 3.12) |
| 0.99 (0.19, 5.03) | 0.25 (0.04, 1.38) | | | 0.64 (0.07, 5.41) | | | 0.41 (0.02, 8.32) | | | 0.28 (0.05, 1.64) | | **Pitavastatin** | | | 0.56 (0.08, 3.27) | | | 0.36 (0.07, 2.24) |
| 1.76 (0.82, 4.35) | 0.44 (0.17, 1.27) | | | 1.13 (0.25, 6.04) | | | 0.72 (0.06, 11.07) | | | 0.50 (0.19, 1.55) | | 1.77 (0.31, 12.06) | | | **Rosuvastatin** | | | 0.64 (0.25, 2.03) |
| 2.77 (1.31, 5.09) | 0.69 (0.32, 1.22) | | | 1.77 (0.42, 6.89) | | | 1.12 (0.10, 14.08) | | | 0.80 (0.32, 1.66) | | 2.80 (0.45, 15.31) | | | 1.55 (0.49, 4.01) | | | **Simvastatin** |
| **Renal insufficiency** | | | | | | | | | | | | | | | | | | |
| **Atorvastatin** | | | | 1.28 (0.32, 5.09) | | | | | | 1.11 (0.35, 3.45) | | | | | 0.90 (0.25, 3.01) | | | |
| 0.78 (0.20, 3.14) | | | | **Control** | | | | | | 0.87 (0.19, 3.89) | | | | | 0.70 (0.17, 2.75) | | | |
| 0.90 (0.29, 2.86) | | | | 1.14 (0.26, 5.34) | | | | | | **Rosuvastatin** | | | | | 0.81 (0.29, 2.20) | | | |
| 1.11 (0.33, 3.40) | | | | 1.43 (0.36, 5.75) | | | | | | 1.24 (0.45, 3.48) | | | | | **Simvastatin** | | | |
| **Gastrointestinal discomfort** | | | | | | | | | | | | | | | | | | |
| **Atorvastatin** | | | 0.83 (0.37, 2.47) | | | 1.26 (0.22, 9.25) | | | 0.83 (0.25, 3.06) | | | | 0.77 (0.19, 2.51) | | | 0.35 (0.15, 1.09) | | |
| 1.20 (0.41, 2.71) | | | **Control** | | | 1.50 (0.30, 7.53) | | | 0.97 (0.32, 2.79) | | | | 0.92 (0.15, 3.67) | | | 0.42 (0.14, 1.33) | | |
| 0.79 (0.11, 4.58) | | | 0.67 (0.13, 3.37) | | | **Fluvastatin** | | | 0.65 (0.09, 4.40) | | | | 0.61 (0.05, 4.78) | | | 0.28 (0.04, 2.08) | | |
| 1.21 (0.33, 3.96) | | | 1.03 (0.36, 3.17) | | | 1.54 (0.23, 11.12) | | | **Pravastatin** | | | | 0.94 (0.13, 4.77) | | | 0.44 (0.11, 1.96) | | |
| 1.29 (0.40, 5.26) | | | 1.09 (0.27, 6.85) | | | 1.64 (0.21, 20.17) | | | 1.06 (0.21, 7.69) | | | | **Rosuvastatin** | | | 0.46 (0.11, 3.01) | | |
| 2.83 (0.92, 6.55) | | | 2.38 (0.75, 7.21) | | | 3.56 (0.48, 25.11) | | | 2.30 (0.51, 9.28) | | | | 2.16 (0.33, 8.78) | | | **Simvastatin** | | |
| **Cancer** | | | | | | | | | | | | | | | | | | |
| **Atorvastatin** | | 1.51e+05 (2.15, 1.49e+15) | | | 1.39e+05 (1.87, 1.39e+15) | | | 1.93e+05 (2.29, 2.08e+15) | | | 1.55e+05 (2.20, 1.50e+15) | | | 1.49e+11 (126.9, 1.72e+24) | | | 1.50e+05 (2.09, 1.47e+15) | |
| 6.61e-06 (6.71e-16, 0.47) | | **Control** | | | 0.92 (0.42, 2.01) | | | 1.23 (0.30, 5.16) | | | 1.04 (0.59, 1.56) | | | 1.30e+05 (1.73, 1.09e+16) | | | 1.00 (0.58, 1.63) | |
| 7.22e-06 (7.18e-16, 0.54) | | 1.09 (0.50, 2.38) | | | **Fluvastatin** | | | 1.34 (0.27, 6.84) | | | 1.13 (0.41, 2.61) | | | 1.42e+05 (1.83, 1.20e+16) | | | 1.08 (0.42, 2.69) | |
| 5.17e-06 (4.81e-16, 0.44) | | 0.81 (0.19, 3.32) | | | 0.74 (0.15, 3.74) | | | **Lovastatin** | | | 0.84 (0.17, 3.63) | | | 9.92e+04 (1.20, 1.11e+16) | | | 0.81 (0.18, 3.51) | |
| 6.46e-06 (6.67e-16, 0.46) | | 0.96 (0.64, 1.71) | | | 0.89 (0.38, 2.43) | | | 1.20 (0.28, 5.61) | | | **Pravastatin** | | | 1.29e+05 (1.71, 1.05e+16) | | | 0.95 (0.51, 2.09) | |
| 6.73e-12 (5.82e-25, 0.01) | | 7.65e-06 (9.21e-17, 0.58) | | | 7.06e-06 (8.35e-17, 0.55) | | | 1.01e-05 (8.99e-17, 0.83) | | | 7.76e-06 (9.50e-17, 0.58) | | | **Pitavastatin** | | | 7.58e-06 (9.05e-17, 0.58) | |
| 6.68e-06 (6.80e-16, 0.48) | | 1.00 (0.61, 1.72) | | | 0.93 (0.37, 2.40) | | | 1.24 (0.28, 5.70) | | | 1.05 (0.48, 1.98) | | | 1.32e+05 (1.71, 1.11e+16) | | | **Simvastatin** | |

**Table S11. Rank probabilities of different intervention in network meta-analyses**

| **Transaminase elevations** | | | | | | | | | | | | | | | | | | | | |
| --- | --- | --- | --- | --- | --- | --- | --- | --- | --- | --- | --- | --- | --- | --- | --- | --- | --- | --- | --- | --- |
|  | **Rank 1** | | **Rank 2** | | | **Rank 3** | | | | **Rank 4** | | **Rank 5** | | | **Rank 6** | | | **Rank 7** | | **Rank 8** |
| **Atorvastatin** | 0.000 | | 0.000 | | | 0.000 | | | | 0.004 | | 0.050 | | | 0.227 | | | 0.452 | | 0.267 |
| **Control** | 0.381 | | 0.406 | | | 0.164 | | | | 0.041 | | 0.008 | | | 0.001 | | | 0.000 | | 0.000 |
| **Fluvastatin** | 0.035 | | 0.041 | | | 0.069 | | | | 0.139 | | 0.215 | | | 0.205 | | | 0.156 | | 0.140 |
| **Lovastatin** | 0.305 | | 0.066 | | | 0.075 | | | | 0.108 | | 0.107 | | | 0.092 | | | 0.087 | | 0.160 |
| **Pravastatin** | 0.176 | | 0.303 | | | 0.295 | | | | 0.157 | | 0.054 | | | 0.013 | | | 0.002 | | 0.000 |
| **Pitavastatin** | 0.033 | | 0.025 | | | 0.032 | | | | 0.062 | | 0.108 | | | 0.148 | | | 0.189 | | 0.403 |
| **Rosuvastatin** | 0.028 | | 0.039 | | | 0.075 | | | | 0.179 | | 0.293 | | | 0.255 | | | 0.102 | | 0.029 |
| **Simvastatin** | 0.043 | | 0.121 | | | 0.289 | | | | 0.311 | | 0.166 | | | 0.057 | | | 0.012 | | 0.001 |
| **Renal insufficiency** | | | | | | | | | | | | | | | | | | | | |
|  | **Rank 1** | | | | | | **Rank 2** | | | | | | **Rank 3** | | | | | | **Rank 4** | |
| **Atorvastatin** | 0.296 | | | | | | 0.241 | | | | | | 0.273 | | | | | | 0.190 | |
| **Control** | 0.171 | | | | | | 0.183 | | | | | | 0.203 | | | | | | 0.443 | |
| **Rosuvastatin** | 0.149 | | | | | | 0.277 | | | | | | 0.298 | | | | | | 0.276 | |
| **Simvastatin** | 0.384 | | | | | | 0.299 | | | | | | 0.226 | | | | | | 0.091 | |
| **Gastrointestinal discomfort** | | | | | | | | | | | | | | | | | | | | |
|  | **Rank 1** | | | | **Rank 2** | | | | **Rank 3** | | | **Rank 4** | | | | **Rank 5** | | | **Rank 6** | |
| **Atorvastatin** | 0.004 | | | | 0.055 | | | | 0.172 | | | 0.277 | | | | 0.329 | | | 0.162 | |
| **Control** | 0.012 | | | | 0.165 | | | | 0.316 | | | 0.282 | | | | 0.179 | | | 0.046 | |
| **Fluvastatin** | 0.052 | | | | 0.097 | | | | 0.098 | | | 0.108 | | | | 0.147 | | | 0.497 | |
| **Pravastatin** | 0.064 | | | | 0.241 | | | | 0.208 | | | 0.175 | | | | 0.181 | | | 0.132 | |
| **Rosuvastatin** | 0.132 | | | | 0.275 | | | | 0.153 | | | 0.133 | | | | 0.150 | | | 0.157 | |
| **Simvastatin** | 0.736 | | | | 0.168 | | | | 0.053 | | | 0.025 | | | | 0.013 | | | 0.006 | |
| **Cancer** | | | | | | | | | | | | | | | | | | | | |
|  | | **Rank 1** | | **Rank 2** | | | | **Rank 3** | | | **Rank 4** | | | **Rank 5** | | | **Rank 6** | | | **Rank 7** |
| **Atorvastatin** | | 0.983 | | 0.004 | | | | 0.002 | | | 0.001 | | | 0.003 | | | 0.007 | | | 0.000 |
| **Control** | | 0.001 | | 0.070 | | | | 0.274 | | | 0.379 | | | 0.224 | | | 0.050 | | | 0.000 |
| **Fluvastatin** | | 0.006 | | 0.378 | | | | 0.198 | | | 0.111 | | | 0.168 | | | 0.137 | | | 0.002 |
| **Lovastatin** | | 0.006 | | 0.264 | | | | 0.085 | | | 0.049 | | | 0.080 | | | 0.505 | | | 0.012 |
| **Pravastatin** | | 0.001 | | 0.122 | | | | 0.185 | | | 0.204 | | | 0.300 | | | 0.186 | | | 0.001 |
| **Pitavastatin** | | 0.000 | | 0.004 | | | | 0.002 | | | 0.001 | | | 0.001 | | | 0.008 | | | 0.983 |
| **Simvastatin** | | 0.003 | | 0.157 | | | | 0.255 | | | 0.254 | | | 0.223 | | | 0.106 | | | 0.001 |

**Figure S5.** Cumulative ranking plot of different types of statins in network meta-analysis


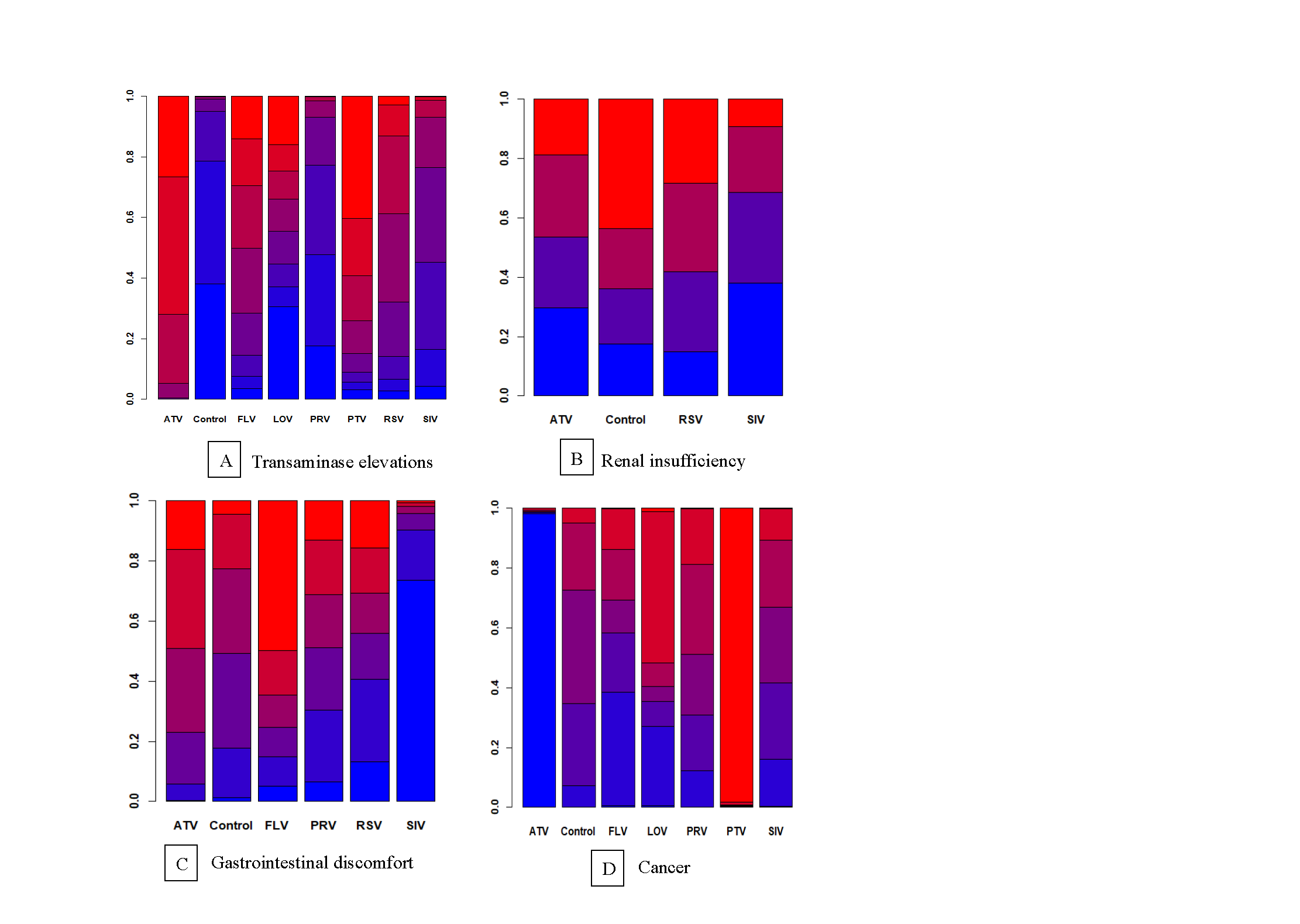


**Table S12.** The SUCRA results in the dose-response meta-analyses

| **Transaminase elevations** | | |
| --- | --- | --- |
| **Treatment** | **Emax** | **ED50** |
| Atorvastatin | 1.80 | 2.49 |
| Fluvastatin | 3.19 | 3.33 |
| Lovastatin | 4.11 | 4.14 |
| Pravastatin | 4.18 | 3.54 |
| Pitavastatin | 3.87 | 4.00 |
| Rosuvastatin | 3.77 | 3.89 |
| Simvastatin | 3.09 | 2.62 |
| **Renal insufficiency** | | |
| **Treatment** | **Emax** | **ED50** |
| Atorvastatin | 1.38 | 1.38 |
| Rosuvastatin | 1.37 | 1.28 |
| Simvastatin | 1.25 | 1.34 |
| **Gastrointestinal discomfort** | | |
| **Treatment** | **Emax** | **ED50** |
| Atorvastatin | 1.64 | 2.22 |
| Fluvastatin | 2.07 | 2.43 |
| Pravastatin | 1.73 | 2.29 |
| Rosuvastatin | 2.64 | 2.44 |
| Simvastatin | 3.92 | 2.62 |
| **Cancer** | | |
| **Treatment** | **Emax** | **ED50** |
| Atorvastatin | 3.81 | 3.58 |
| Fluvastatin | 3.32 | 2.77 |
| Lovastatin | 2.66 | 2.99 |
| Pravastatin | 2.39 | 2.66 |
| Pitavastatin | 2.10 | 3.12 |
| Simvastatin | 3.22 | 2.48 |

**Figure S6.** Cumulative ranking plot of Emax and ED50 parameters for different statins

A

Muscle condition


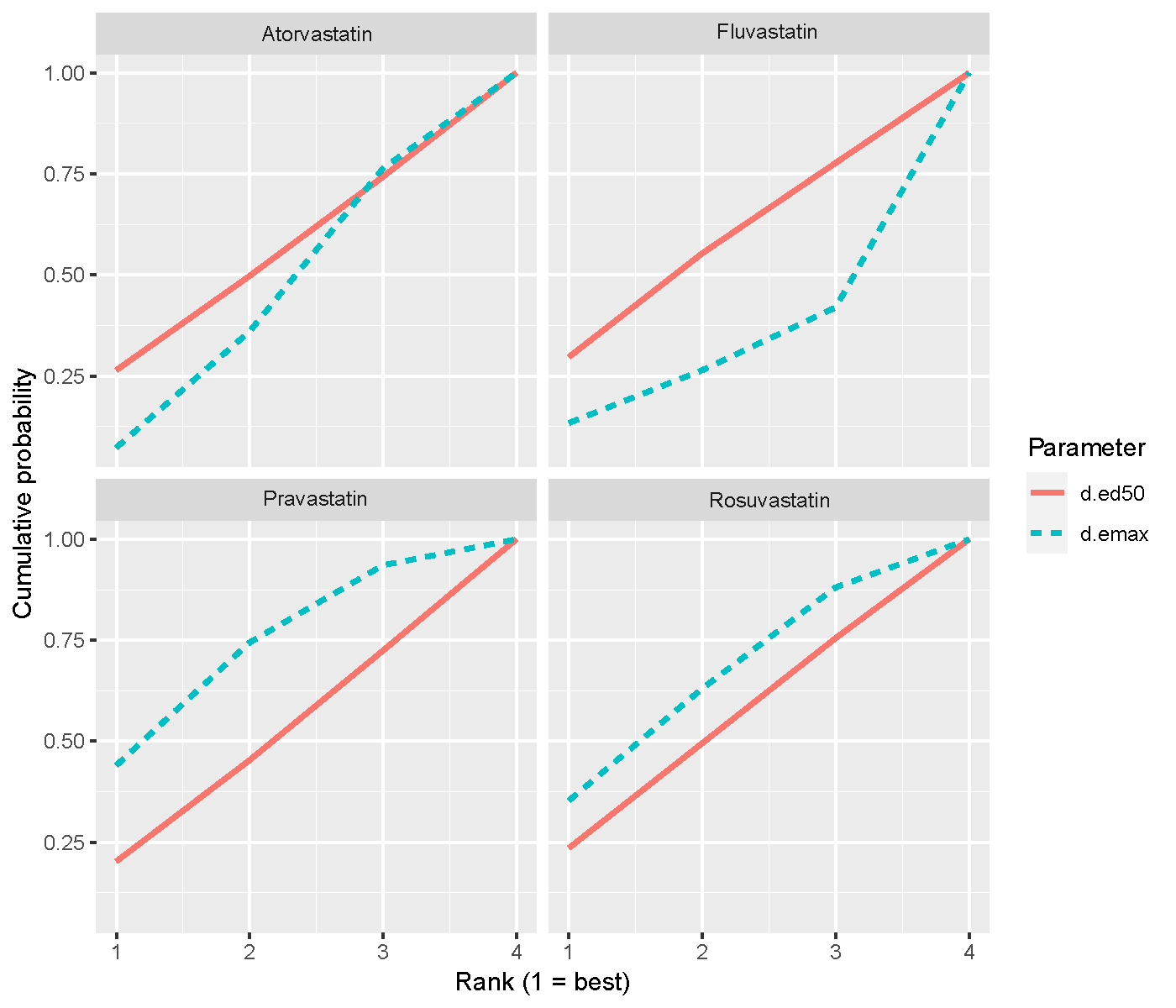


B

Transaminase elevations


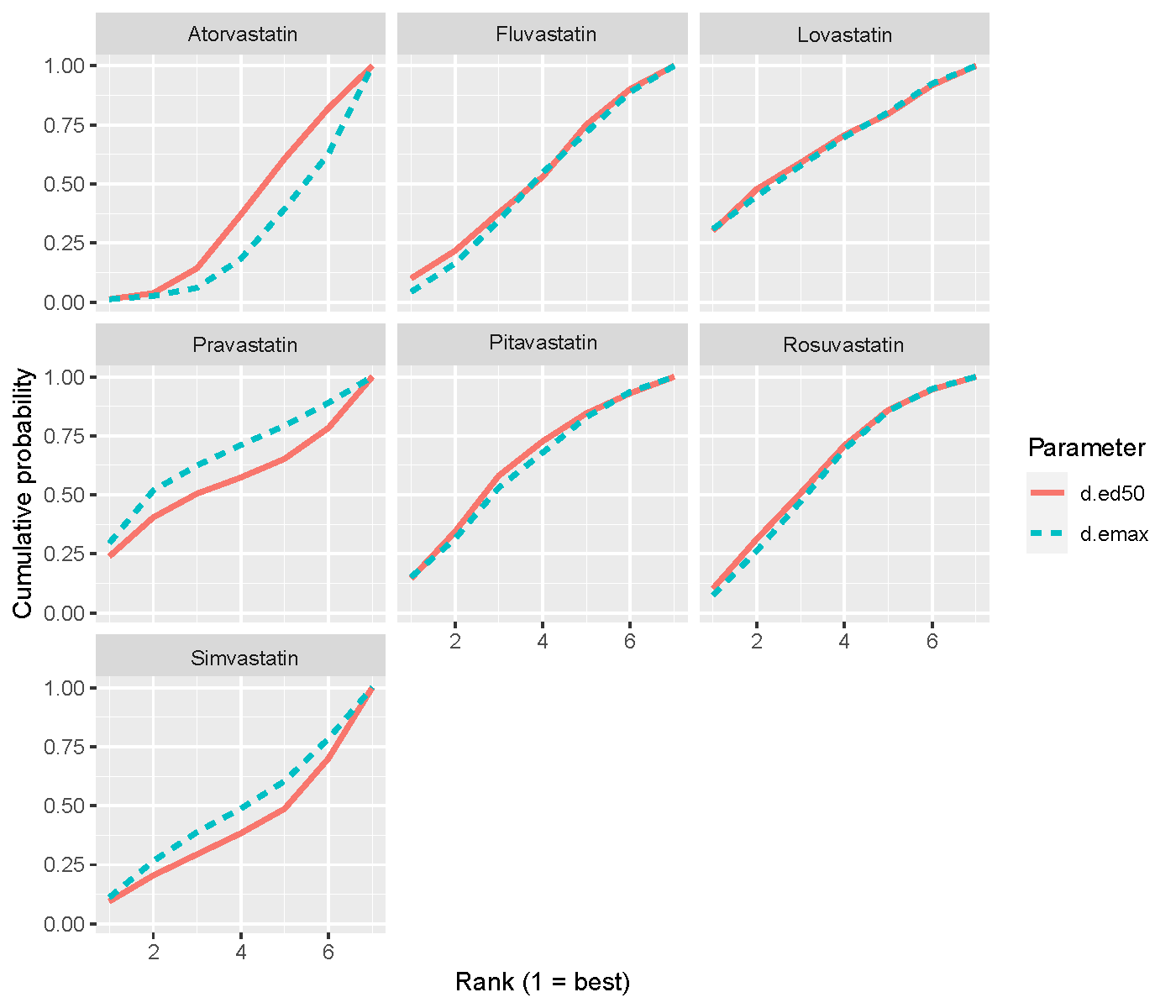


C

Renal Insufficiency


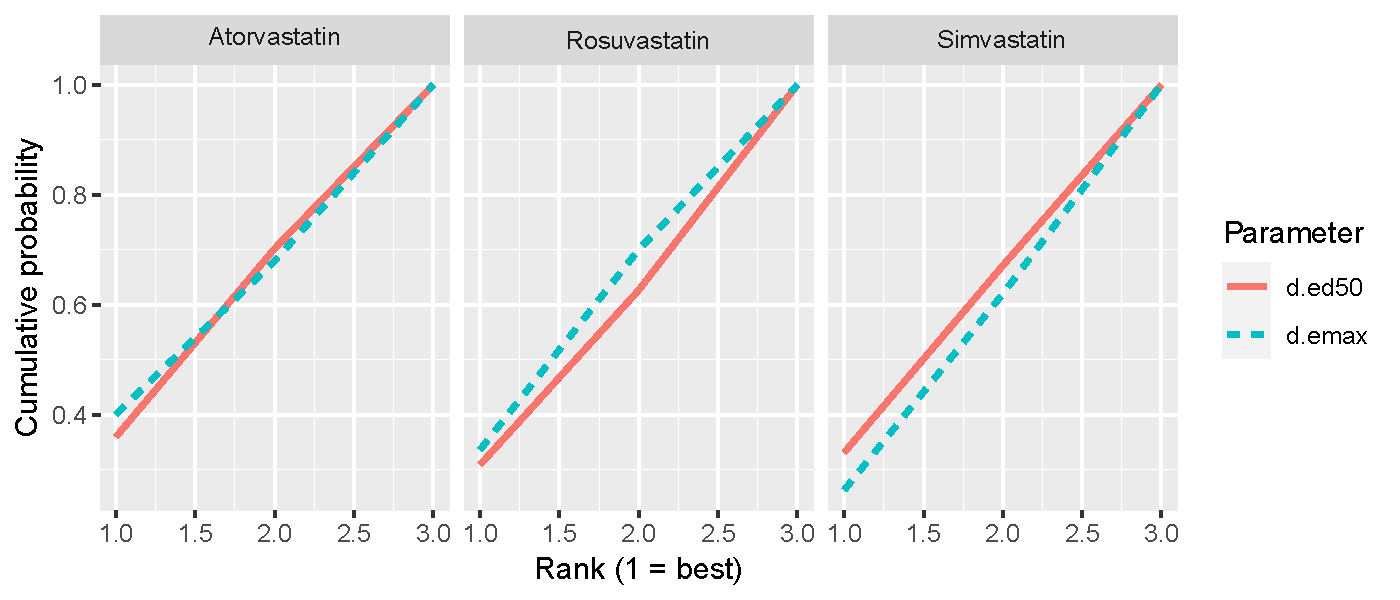


D

Gastrointestinal discomfort

**
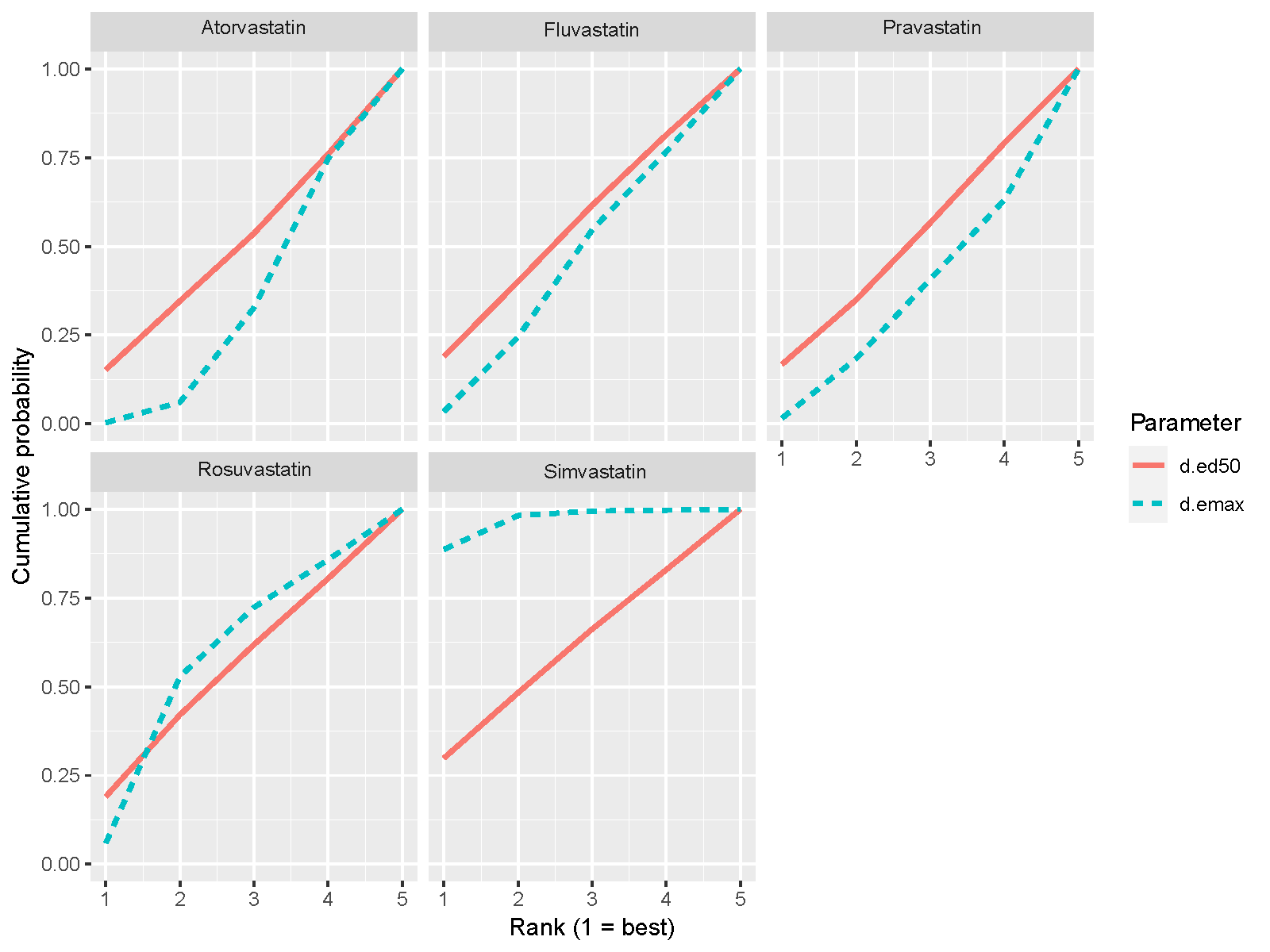
**

E

Cancer

**
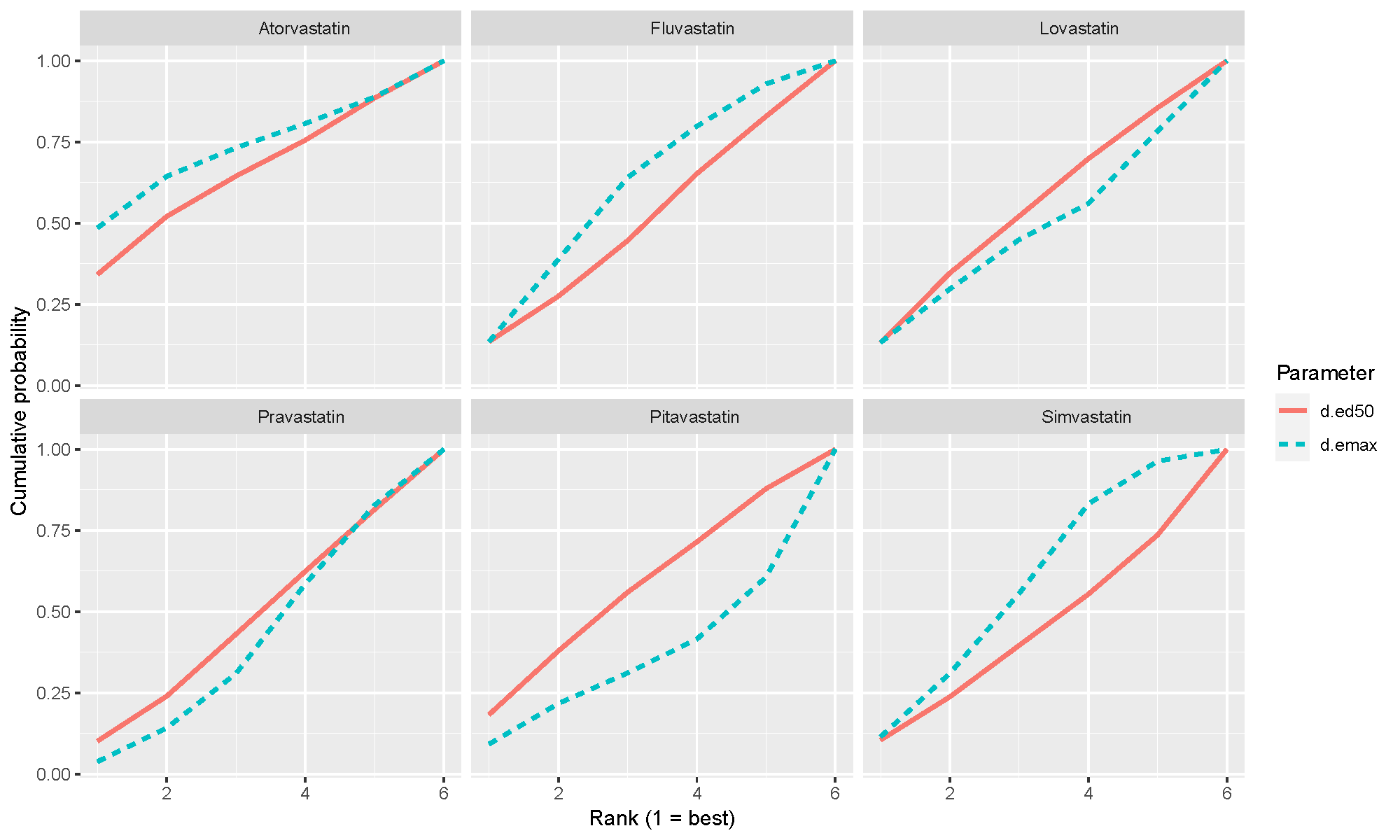
**

**Figure S7. Emax dose-response curves with dose-specific adverse effects of individual statins**

A

Muscle condition


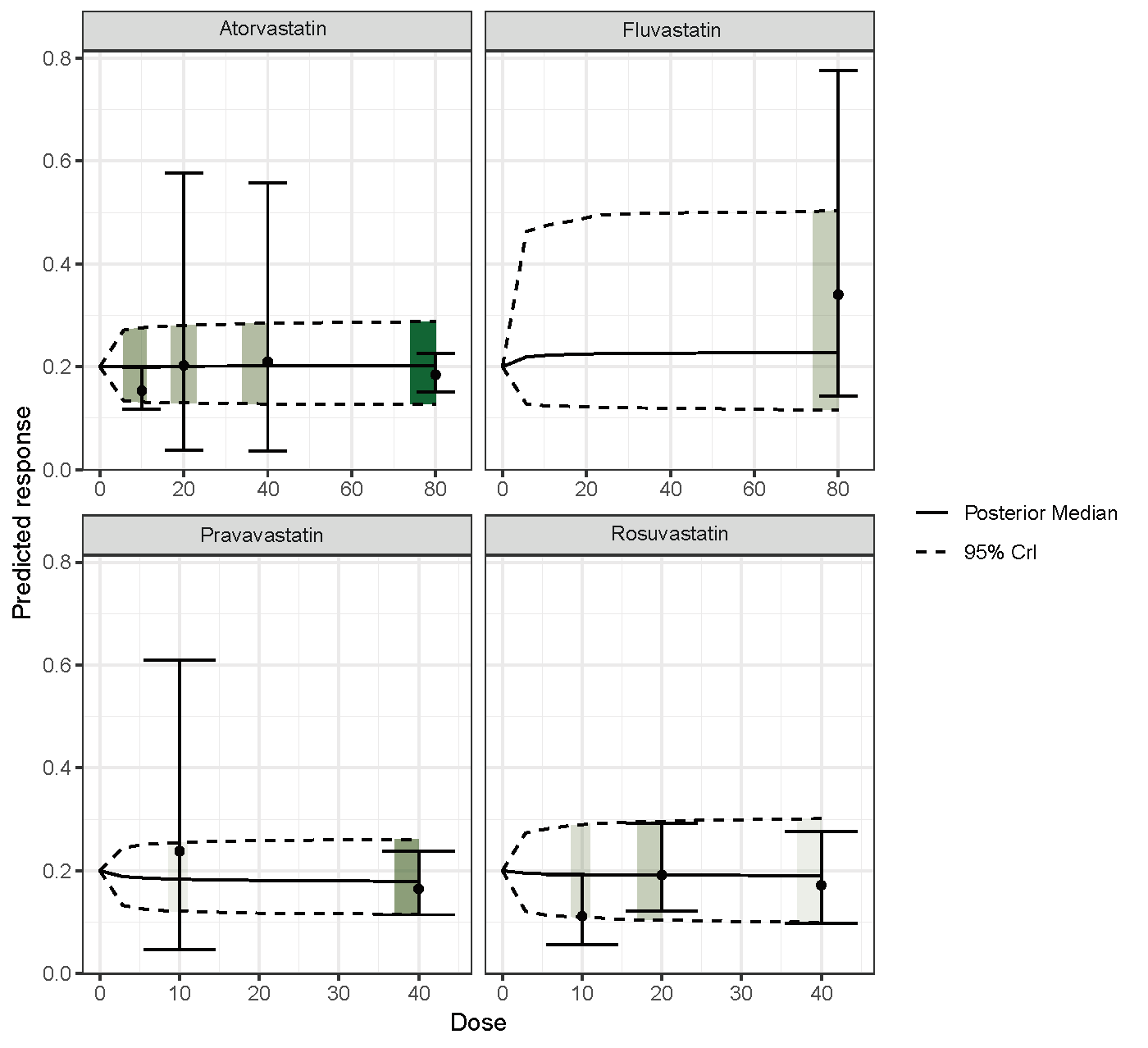


B

Transaminase elevations


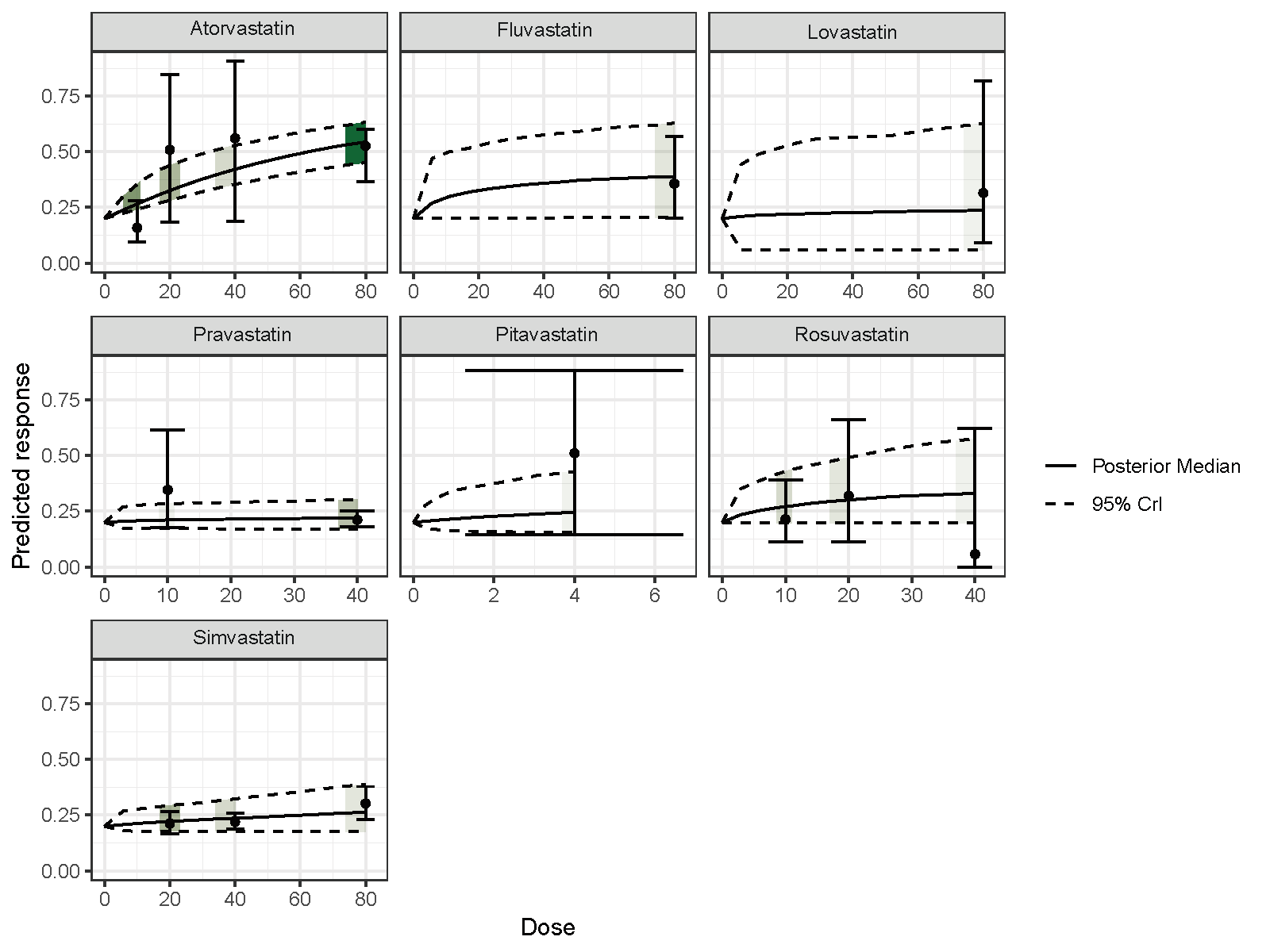


C

Renal Insufficiency


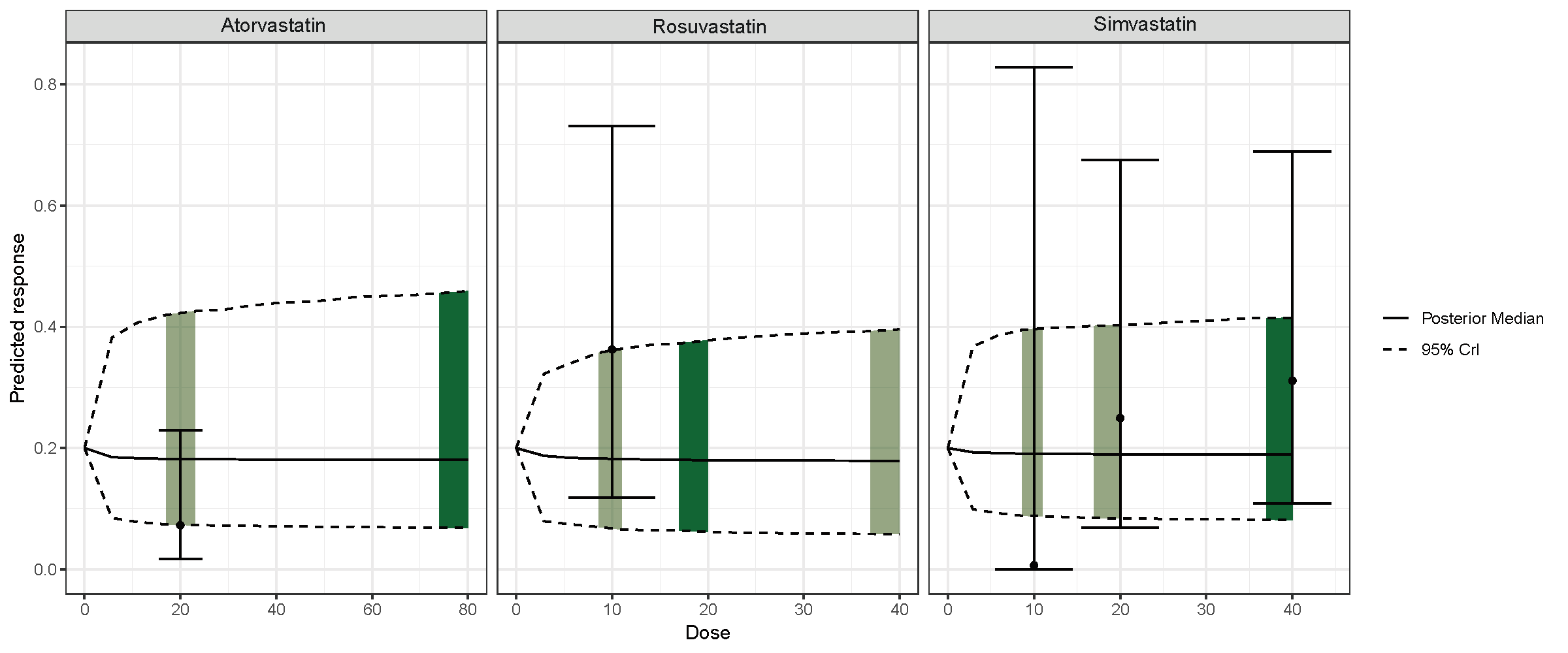


D

Gastrointestinal discomfort

**
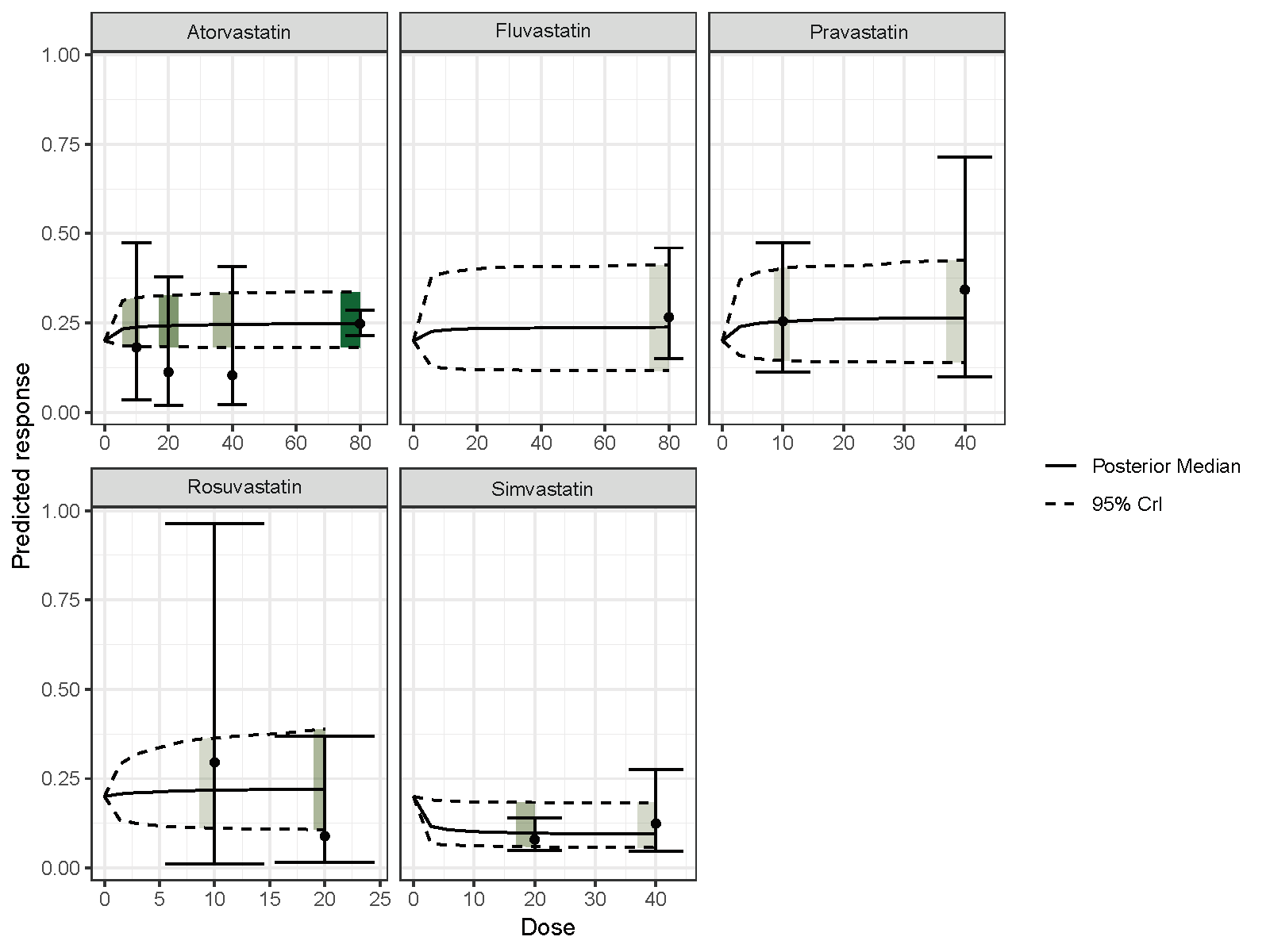
**

E

Cancer

**
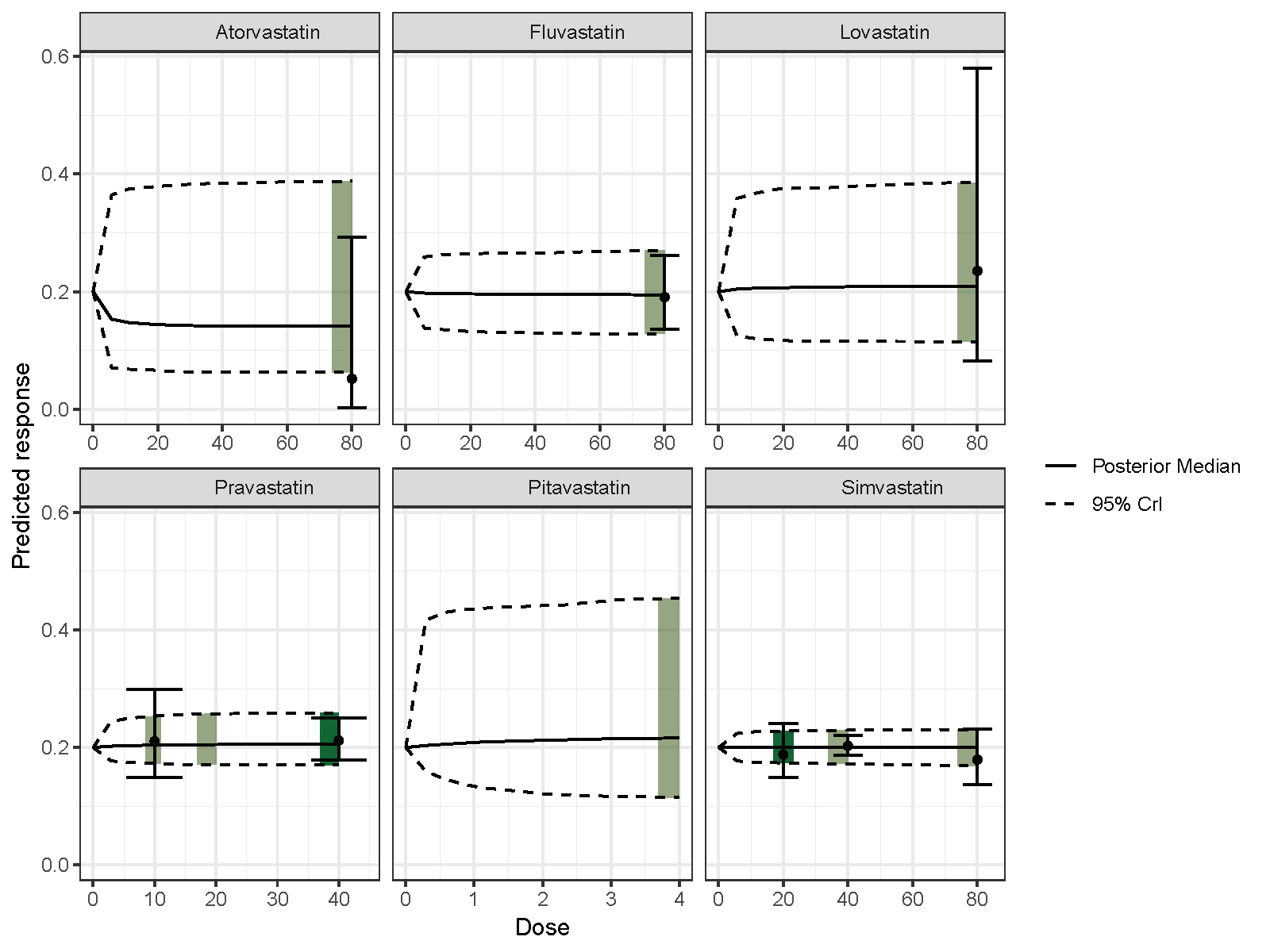
**
